# Opportunities for nanomaterials in more sustainable aviation

**DOI:** 10.1186/s11671-024-04087-5

**Published:** 2024-12-18

**Authors:** Afshin Pendashteh, Anastasiia Mikhalchan, Tamara Blanco Varela, Juan J. Vilatela

**Affiliations:** 1 IMDEA Materials Institute, C/Eric Kandel 2, 28906 Getafe, Madrid, Spain; 2 AIRBUS Operation, P/John Lennon, S/N, 28906 Getafe, Madrid, Spain

**Keywords:** Nanotube, Nanowire, Nanocomposite, Energy, Aircraft, Battery

## Abstract

New materials for electrical conductors, energy storage, thermal management, and structural elements are required for increased electrification and non-fossil fuel use in transport. Appropriately assembled as macrostructures, nanomaterials can fill these gaps. Here, we critically review the materials science challenges to bridge the scale between the nanomaterials and the large-area components required for applications. We introduce a helpful classification based on three main macroscopic formats (fillers in a matrix, random sheets or aligned fibres) of high-aspect ratio nanoparticles, and the corresponding range of bulk properties from the commodity polymer to the high-performance fibre range. We review progress over two decades on macroscopic solids of nanomaterials (CNTs, graphene, nanowires, etc*.*), providing a framework to rationalise the transfer of their molecular-scale properties to the scale of engineering components and discussing strategies that overcome the envelope of current aerospace materials. Macroscopic materials in the form of organised networks of high aspect ratio nanomaterials have higher energy density than regular electrodes, superior mechanical properties to the best carbon fibres, and electrical and thermal conductivity above metals. Discussion on extended electrical properties focuses on nanocarbon-based materials (e.g., doped or metal-hybridised) as power or protective conductors and on conductive nanoinks for integrated conductors. Nanocomposite electrodes are enablers of hybrid/electric propulsion by eliminating electrical transport limitations, stabilising emerging high energy density battery electrodes, through high-power pseudocapacitive nanostructured networks, or downsizing Pt-free catalysts in flying fuel cells. Thermal management required in electrified aircraft calls for nanofluids and loop heat pipes of nanoporous conductors. Semi-industrial interlaminar reinforcement using nanomaterials addresses present structural components. Estimated improvements for mid-range aircraft include > 1 tonne weight reduction, eliminating hundreds of CO_2_ tonnes released per year and supporting hybrid/electric propulsion by 2035.

## Introduction

As one of the most technically demanding industries, aerospace innovation has always gone hand in hand with developing advanced materials. Previous civil aircraft programmes were interlinked with broader technological endeavours for the development of superalloys and structural laminate composites. A pressing environmental emergency in the context of ever-increasing mobility has unleashed a new aviation development cycle, primarily focused on electrification and the use of non-fossil fuels. It presents challenges for the transport industry centred around the storage, conversion, and transfer of energy, as well as new conceptions of the interrelation between materials, systems, and multifunctionality.

Aviation history shows that new challenges require new materials and new fabrication processes. This paper analyses the potential of nanomaterials to be key building blocks for the next generation sustainable aircraft. It provides an overview of the major trends for the introduction of nanomaterials in commercial aircraft within 15 years. It is not intended as an exhaustive review of specific properties or particular materials; instead, we use case studies to illustrate promising developments and technologies in line with industrial trends and identify the most critical challenges for the near future.

The first part of the paper *introduces the sustainability targets for commercial aviation* in the next 20 years and the industrial vision to achieve them. Of the several initiatives to tackle these challenges, we focus on increased electrification, known in the industry as the More Electric Aircraft (MEA), and on non-fossil alternative fuel propulsion, e.g., H_2_, leading to carbon–neutral emission aircraft. With a set of general requirements from these initiatives, the paper then discusses why nanostructured materials have specific properties that could match them in the near future.

In subsequent sections, we *outline the materials that can help address the main challenges* in the aircraft industry by taking advantage of nanotechnology. Specifically, we focus on nanostructured carbon-based materials, such as carbon nanotubes and graphene, which owe graphitic carbonaceous nature coupled with superior theoretical mechanical, electrical, and thermal properties [1, 2]. The review not only summarises the dominant properties driving the most critical applications in the MEA industry (electrical and thermal conductors, energy storage and conversion, structural reinforcement) but draws a clear parallel between the relevant materials specifications in aircraft and the innovative nanocarbon materials capable of fitting the gaps and shifting the performance beyond the current state-of-the-art figures.

Finally, the paper *presents our perception of engineering gaps*, including raw nanomaterials production capacity, processing and integration methods, and the challenges of evaluating materials in emerging components and products under constant evolution. We discuss the practical challenges of maximising their bulk properties, which are mainly guided by the degree of nanocarbons alignment and compaction in the macroscopic formats. The review includes a list of “hot areas for future studies” expected to be critical for future aerospace technologies. We conclude by providing a roadmap for implementing nanomaterials in commercial aircraft that reflects the key ideas discussed in this review.

## Sustainability targets

### Current status of the aerospace sector and post-pandemic projections

The world has faced an unprecedented pandemic that originated with COVID-19, resulting in the most significant aviation industry crisis. The dramatic drop in air transport (mainly passenger, but also freight), the excessive dependence on refined petroleum manufacturing, imposed containment measures, and public policies aimed at aircraft safety raised concerns about the efficient use of environmental, financial, and public resources [3, 4]. World air traffic has almost recovered after three years; according to Official Airline Guide (OAG) reports, global total seats by week have increased continuously since the second quarter of 2019 and are now only 5.6% below 2019 levels [5]. Consequently, aircraft production is ramping up again in line with customer demands. There is, however, a backdrop of sharply rising inflation, increasing energy prices, the cost of additional health and safety-related measures, and shortages of labour, raw materials, electronic components, and more. These factors demonstrate the so-far underestimated importance of the sustainability of the whole aviation value chain and low-carbon transition strategies. Despite volatility and uncertainty in the aerospace sector, the aeronautical industry has publicly refrained from ensuring that sustainability and a circular economy leading to a sustainable recovery remain top priorities in the next two decades [6]. However, given the COVID-19 pandemic, it has become clear that strategic decisions and green industrial policies should foster the resilience of the aviation industry. For instance, future investments in cleaner and more electric aircraft can contribute to the long-run viability and resilience of more energy-efficient transport to global risks [7]. While the major stream of current research in the sustainability sector concerns CO_2_ emissions changes, there is a clear need for feasible solutions. Evolving technologies and materials, especially those where nanostructure forms a basis for a disruptive combination of properties and multifunctionality, can accelerate the shift towards low-carbon aircraft and net-zero CO_2_ emissions target.

### Aviation environmental footprint—the targets

Nowadays, aviation contributes to approximately 2–3% of human-made CO_2_ emissions. However, this could increase by more than twofold in 2050 if the pre-pandemic air traffic growth continues [8]. For many years, this industry has sought to reduce net CO_2_ despite sustained growth of 4% in demand. Significant environmental footprint reductions have already been achieved in this sector over the last 50 years, including reductions of 80% in CO_2_ emissions per seat kilometre, 90% in NO_x_ emissions and 75% in noise [9]. Aeroplanes are quieter and lighter than ever before. This is largely through the introduction of new high-performance materials, such as structural composite materials and new high-performance alloys.

Some of the environmental targets of the aviation industry are encompassed in its Carbon Offsetting and Reduction Scheme for International Aviation (CORSIA), a public–private programme for the gradual implementation of control measures. Its original targets were to reduce CO_2_ emissions by 50% by 2050 (versus 2005) and limit the growth or stay flat in net CO_2_ emissions from 2020 onwards (carbon–neutral growth) [10]. In 2016, Governments adopted CORSIA to stabilise net CO_2_ emissions from international aviation starting from 2021.

In October 2021, at the 77th IATA Annual General Meeting in Boston, USA, the global air transport industry adopted a long-term climate goal of net zero carbon emissions by 2050 with signatories to a joint declaration representing the world’s airlines, airports, air traffic management, and the makers of aircraft and engines. This pledge brings air transport in line with the objectives of the Paris Agreement to limit global warming to 1.5 °C [11]. Finally, in October 2022 in Montréal—at the 41st International Civil Aviation Organisation (ICAO) Assembly, the Member States adopted a collective long-term global aspirational goal (LTAG) of net-zero carbon emissions by 2050, following the same commitment adopted in 2021 by the global air transport industry [12]. The Air Transport Action Group (ATAG) explored how the aviation sector may be able to meet net-zero CO_2_ emissions by 2050. Three different scenarios have been defined to reach this target, including “Scenario 3: aspirational and aggressive technology perspective”, targeting a combination of electric, hybrid or (green) hydrogen-powered aircraft for different segments, combined with 90% fuel replacement [13].

### Trends in more sustainable aviation

Meeting the challenging aviation environmental and CO_2_ targets requires the introduction of radically new technologies in aircraft that address the main sources of emissions. Life Cycle Assessment of commercial aircraft shows that over 98% of CO_2_ aeroplane emissions are released from aircraft operation, while the remaining 2% of CO_2_ emissions are produced during aircraft manufacturing [14]. Therefore, the first opportunities for CO_2_ footprint reduction are during aircraft flight. Efforts to reduce such emissions are divided into four major technological strategies in order of increasing difficulty and impact [15]:

- Continue to develop aircraft, engine design and technology in a relentless pursuit of improvements in standard fuel efficiency and reduced CO_2_ emissions. For the last 40 years, aircraft and engine technology has reduced CO_2_ emissions by a yearly average of over 1% per passenger mile. This has been the result of significant research in advanced materials, aerodynamic efficiency, digital design and manufacturing methods, turbo-machinery developments, and aircraft systems optimisation. Weight reductions from the introduction of carbon fibre, for example, are credited with reducing 27,000 tonnes of CO_2_ per regional aircraft over ten years [16].
- The commercialisation of sustainable aviation fuels at competitive prices. Around 185,000 commercial flights have already proven that today’s aircraft are ready to use them. More sustainable aviation fuels (SAF) will have to be significantly scaled up to meet future demands. Aircraft climate impact can be reduced up to 80% with SAF, which works with the current aircraft propulsion system and is thus the short-term step measure adopted by aircraft manufacturers to decarbonise the aviation sector. There are a variety of SAF technologies based on different feedstock sources. Biomass and several wastes and residues are the most common sources of feedstock for SAF, which include used fat, oil, and grease, for example, hydro-processed esters and fatty acids (HEFA), which generally consists of used cooking oil and other waste fats, municipal waste or agricultural and forestry waste and residues. Alternative sources synthesised with green hydrogen can produce SAF through power-to-liquid (PtL) technology, known as eFuels. Sources can include captured carbon (for example, from Direct Air Carbon Capture technology) and waste gases from industrial processes.
- Other factors, such as efficient air traffic management and aircraft routing that minimise fuel consumption, also play a vital role.
- Development of electric or hybrid electric propulsion technology in combination with sustainable aviation fuel and accelerating technologies to produce lighter and more electric aircraft. This strategy includes function electrification, electric and hybrid-electric propulsion, digitalisation and artificial intelligence, improved manufacturing technologies and new materials, management of drag and distributing propulsion in new ways.

**Fig. 1:**
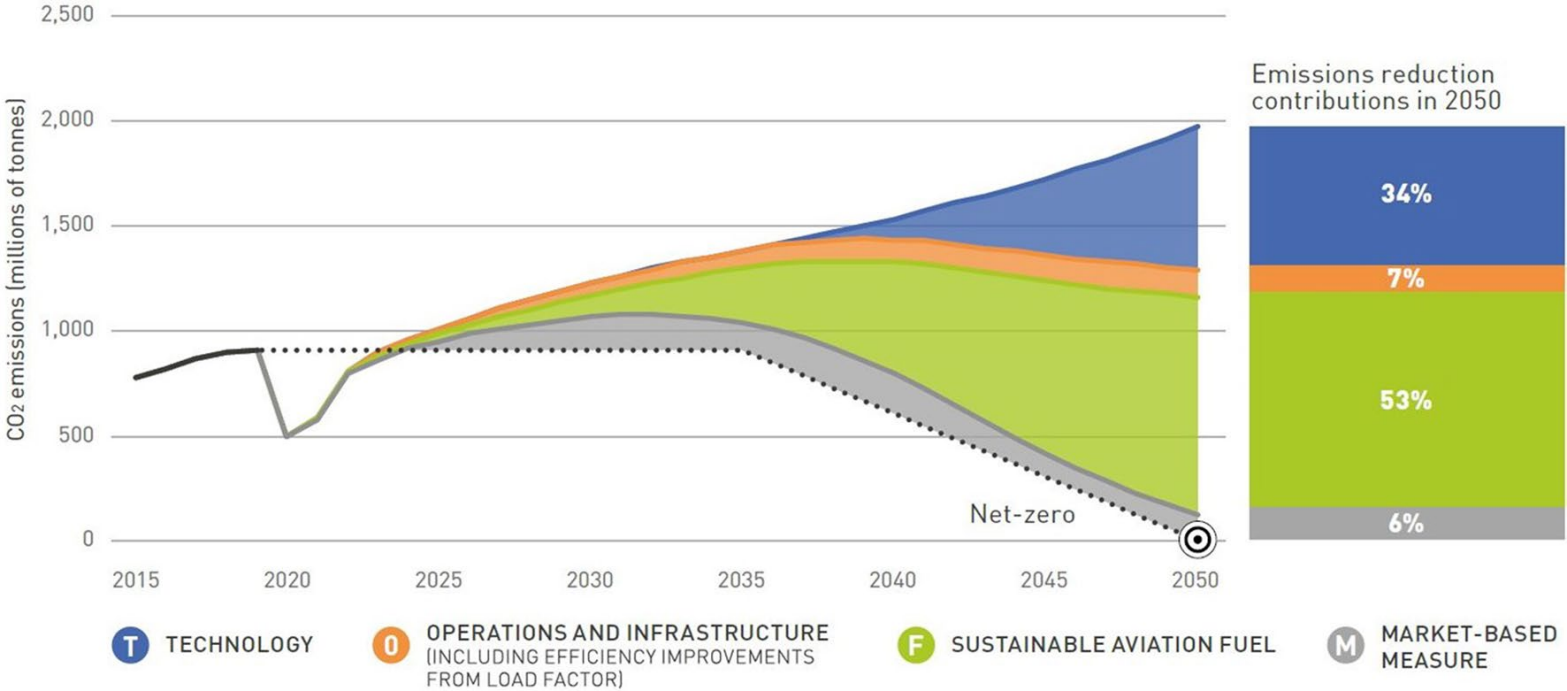
ATAG way point report—Scenario 3: aspirational and aggressive technology perspective [13]. Copyright 2021 The Air Transport Action Group (ATAG)

**Fig. 2:**
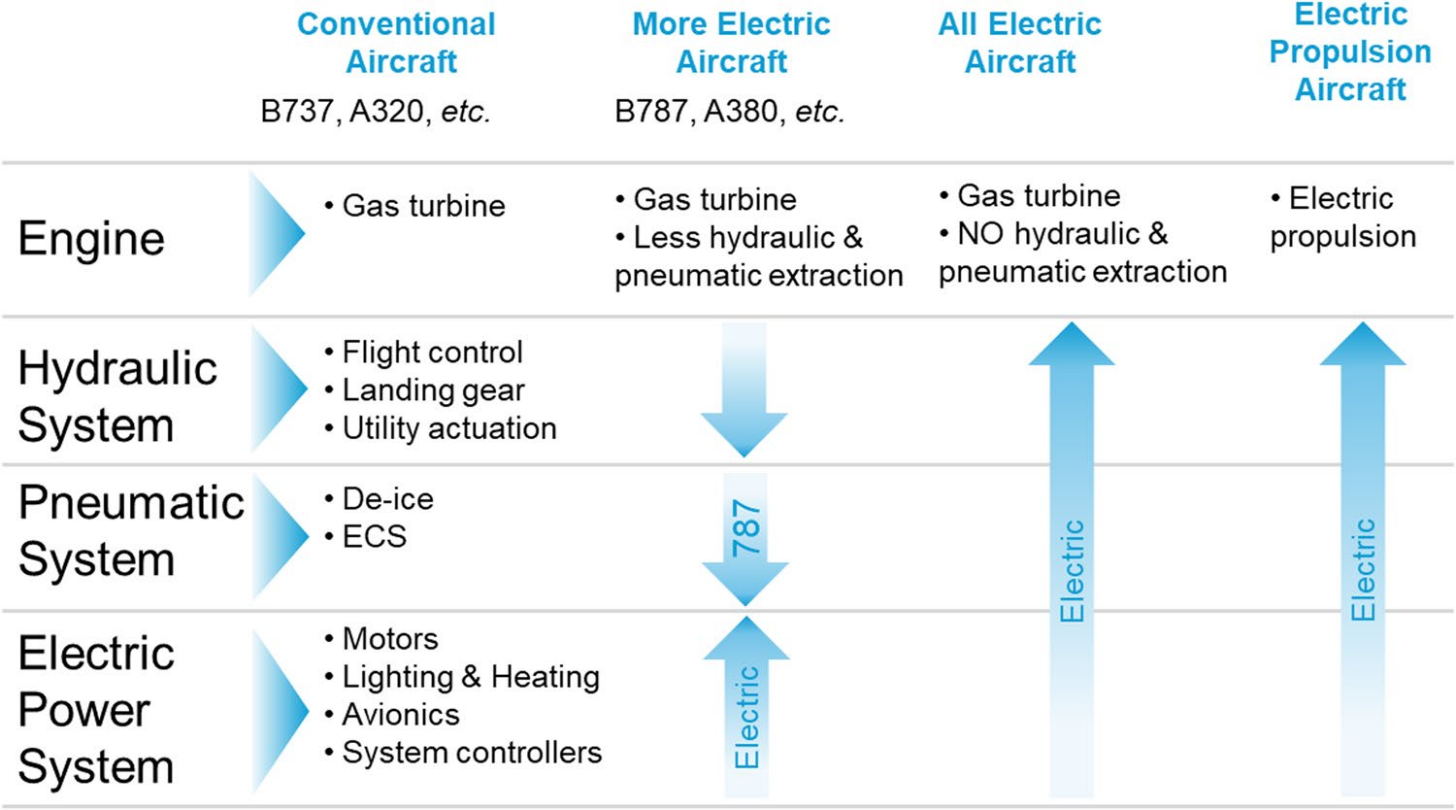
Progression of electric technology for commercial transport aircraft—NASA’s Vision [17]. Copyright 2015 NASA, Work of the US Gov

The transition towards more electric aircraft has been gradual and is generally planned as separated into phases, starting with the electrification of systems (MEA) and progressively electrifying non-electrical functions towards reaching hybrid electric or fully electric propulsion (Figs. 1, 2).

**Fig. 3:**
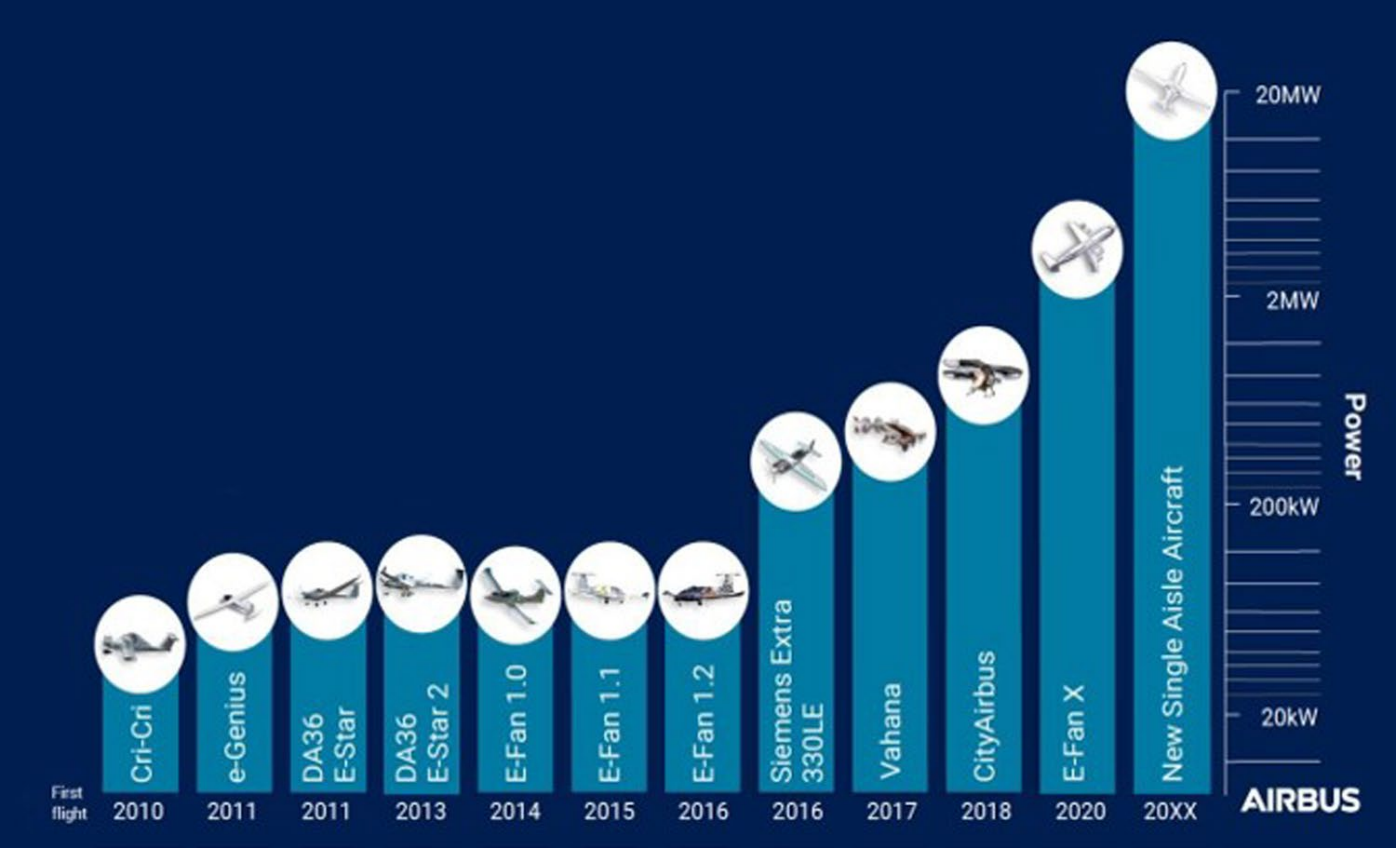
Propulsion power evolution for aviation electrification route—Airbus vision. Copyright 2024 Airbus

Electric and hybrid-electric propulsion is rapidly revolutionising mobility technologies in aviation. As an example, the electrification progression of Airbus aircraft is summarised in Fig. 3. It shows early electric propulsion prototypes setting the basis for future commercialisation of all-electric urban air mobility vehicles and, eventually, small commercial aircraft [9, 18]. This progress can be assessed by considering the evolution of electric propulsive power in commercial aircraft. Figure 3 shows an increase of nearly two orders of magnitude over the last five years, from around 50 kW in a 2-seater concept aircraft in 2015 to over 2 MW in a short-haul regional aircraft in 2020 (E-FanX demonstrator), although still short of the 20 MW required for single-aisle aircraft.

To reduce climate impact and reach the targets of CO_2_ emissions by 2050, the aviation industry will have to introduce further technological levers and breakthroughs, including new clean propulsion energy, such as H_2_ propulsion [19] since the battery technology evolution is not progressing at the required speed for full aviation propulsion electrification. Hydrogen propulsion has significant, so far underestimated, potential to reduce the climate impact of aviation and contribute to its decarbonisation and, thus, to be a major part of the future aviation propulsion technology mix. An intermediate mid-term target is the introduction of an H_2_-powered short-range aircraft by 2035. If H_2_-powered aircraft are deployed in segments where they are the most cost-efficient means of decarbonisation (commuter, regional, short-range, and medium-range aircraft), they could account for 40% of all aircraft by 2050. With synfuel and/or biofuels powering the other 60% aircraft, aviation’s climate impact would then fall by an equivalent of about 2.7 gigatonnes of CO_2_ eq. [20].

The main associated research and engineering activities focus on four key areas: liquid H_2_ (LH_2_) fuel and propulsion components, aircraft systems, infrastructure/ecosystem ramp-up, and the regulatory framework [20]. Airbus’ ambitions for decarbonisation are crystallised in three zero-emission commercial aircraft (ZEROe) that could enter into service by 2035 [21]. These concepts represent different technological and aerodynamic approaches to achieving zero-emission flight, but all rely on liquid hydrogen as a primary power source by direct burn and/or by feeding fuel cells to power electrical motors.

The significant aircraft redesign and introduction of fuel cell propulsion carry multiple challenges in terms of the development of new materials for structural and energy-related functions, some of which are reviewed in Sect. 5. We note, however, that commercialisation and certification of aircraft can take more than ten years and substantial fleet replacement another ten years. It is expected that in 2035, nearly 100% of operating commercial aircraft will not be hybrid-electric yet. Therefore, it is imperative to also seek opportunities to improve today’s conventional propulsion aircraft to reduce emissions over the following decades.

## New (nano)materials for new challenges

### Why nanomaterials?

The new propulsion schemes and increasing electrification of aircraft are more than routine changes in the aviation development cycle. By affecting the core design of aircraft and introducing energy management as a key variable, these trends require the adoption of materials foreign to conventional aircraft and, in some cases, the development of new materials altogether. Put simply, new aviation challenges require new materials.

**Fig. 4:**
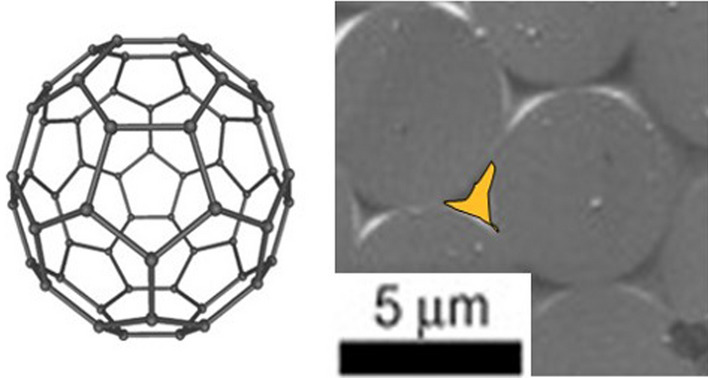
Comparison of the size of a nanoparticle (C60 molecule) and the “small” gap between closed-packed CF filaments in a structural composite. Copyright 2024 IMDEA Materials

Nanomaterials are set to hold a special place in this quest. They are building blocks with at least one dimension below 100 nm, comparable to the size of a macromolecule. To put this into perspective, in the “small” gap between closed-packed CF filaments in a structural composite there is enough space for around one million nanometric particles (Fig. 4).

Their size, properties and manufacturing routes are often very different from those of traditional monolithic materials, suggesting that they can fill gaps in properties available with current industrial materials. Here, we focus on nanomaterials that can potentially contribute to reducing aviation emissions, either through a reduction in the weight of aeronautical components or by enabling less C-intensive propulsion schemes. Their rich optoelectronics properties make nanomaterials equally promising in applications such as avionics, passenger safety comfort, etc*.*, but which are outside the scope of this review.

The potential of nanomaterials to enable access to properties outside the envelope of materials currently used in civil aviation stems both from the inherent properties of nanomaterials as individual “molecules” and from the possibility of assembling them into non-conventional macroscopic architectures.

### Molecular-scale properties

**Fig. 5:**
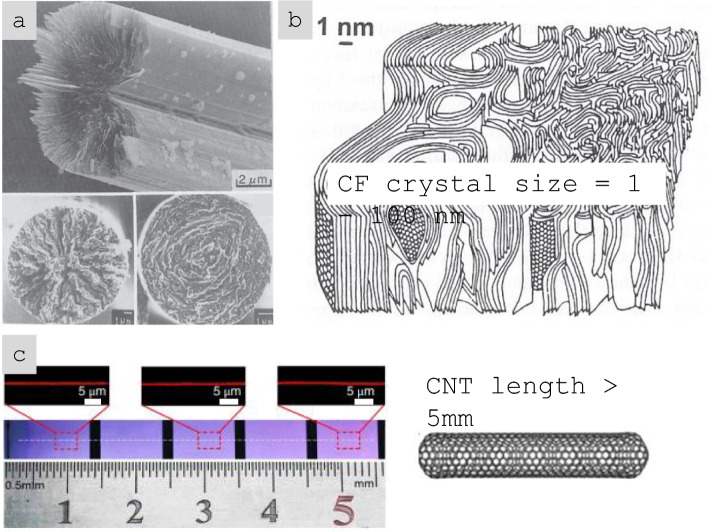
Comparison of crystalline domains in **a**, **b** CF with **c** the length of a CNT (a single, continuous layer of graphene) extending over centimetres [41, 42]. **a**, **b** Adapted from [41] with permission. Copyright 2005 Springer Nature. **c** Adapted from [42] with permission. Copyright 2018 Springer Nature

In terms of molecular-scale properties, nanomaterials can have exceptionally high crystallinity over extended domain sizes. This can be appreciated by analogy to carbon fibre, a well-known aeronautical material. The size of crystalline domains in CFs is of the order of 1–100 nm, depending on precursor, graphitisation temperature, etc*.* Nanocarbons such as graphene (G) or carbon nanotubes (CNTs) can be seen as molecules or particles with nearly perfect graphitic layers extending to the centimetre range [22] (Fig. 5). This high degree of perfection implies that they exhibit properties approaching those of an ideal single crystal. For graphite, this corresponds to a strength of 100 GPa, modulus of 1TPa and thermal conductivity above 1000 W/mK, which have been measured on individual CNTs [23–25] and graphene [26–28]. Thermal conductivity as high as 5000 W/mK was experimentally observed for an individual SWCNT [29] and single-layer graphene [30], close to the theoretical prediction [31]. Similarly, highly crystalline Boron Nitride nanotubes (BNNTs) exhibit axial Young´s modulus in the range of 0.8–1.2 TPa [32–34]. Although the experimentally measured thermal conductivity of BNNTs and nanosheets (few-layer hexagonal BN) is around 200–500 W/mK [35–38], it may theoretically reach 2000 W/mK for BN nanoribbons with low defect density and longer phonon mean free path of 530 nm [39] and 5000 W/mK for a crystallite size approaching 10 µm [40].

The high crystallinity in nanocarbons also manifests in charge mobility above 50,000 cm^2^V/s [43], which is, for reference, close to two orders of magnitude above Silicon. This makes nanocarbons good electrical conductors, with electrical conductivity approaching 10^8^ S/m, which is significantly above metals. The observation of ballistic transport in individual defect-free metallic SWCNTs suggests that bulk conductivities in the range of superconductors may also be attainable. A high charge mobility and chemical stability make nanocarbons tolerant to exceptionally high current densities, with maximum current densities above the level of Cu recorded on individual CNTs [15].

Reducing the size of the material to the nanoscale has the additional effect of eliminating defects and mechanical failure modes encountered in bulk, for example, leading to inorganic nanomaterials that are damage tolerant. Silicon, for example, can be deformed to 10% as 1D nanowires [44], which contrasts with the brittleness of monolithic Si, which cannot withstand more than 0.1% of tensile deformation [45]. As an anode in a Lithium-ion battery, Si can reach ten times the capacity of graphite, the current choice of LIB anode, but only as small silicon (nano)particles, it can effectively withstand repeated cycles of electrochemical alloying with Lithium. For diameters below about 200 nm, Silicon nanowires (SiNWs) retain their 1D structure after repeated charge/discharge cycles and, thus, a high anode Coulombic efficiency and high capacity retention [46].

Nanomaterials’ third key molecular-scale property is a high surface-to-volume ratio, more commonly parametrised as specific surface area (SSA, in m^2^/g). For comparison, values of SSA for individual CF are 0.2 m^2^/g, compared to 300–2600 m^2^/g for nanocarbons. Materials with a high surface area are relevant for a myriad of applications. A relevant example is capacitive energy storage processes, such as in electrochemical supercapacitors, where an electrode stores energy through the electrosorption of ions (and/or a surface redox reaction) at a solid/electrolyte interface. High SSA is also beneficial in catalytic reactions, such as those occurring in fuel cells. In addition, a high surface area can favour heat transfer to the surrounding medium (depending on interfacial thermal resistance), which is applicable for thermal management.

An effective way to maximise crystallinity while preserving a high SSA and favouring assembly of macroscopic objects is to synthesise nanomaterials of high aspect ratio (s = length/diameter), either as large 2D sheets or very long and thin 1D nanowires/nanotubes. Reducing at least one dimension of a material to the nanoscale also leads to quantum confinement, with implications on electronic structure and transport properties. Overall, for most applications discussed in this review, the materials of interest are high aspect ratio nanomaterials; thus, the focus is predominantly on those.

Table 1 presents selected properties of nanomaterials measured experimentally, compared against those of the best material currently used in civil aircraft. The comparison highlights the fact that nanomaterials have overall properties that are on a different scale. However, their size is also on a different scale, and thus, the methods to assemble nanomaterials into macroscopic structures are critical to determining their macroscopic properties. Features of their macroscopic organisation can, and often do, dominate over their molecular-scale features.

**Table 1:** Comparison of basic properties of materials used in aircraft, and best experimentally measured values for nanomaterial building blocks

Property normalised by density or mass	Best nanomaterial (experimentally measured)	Best currently used in aircraft	Nanomaterial/current solution
Mechanical: strength (GPa/g/cm^3^)	Nanotubes or nanolayers of carbon or boron nitride, 500	CF, advanced alloys, ≈ 3	167
Electrical: conductivity (S/m/g/cm^3^)	AsF_5_–intercalated graphene 27,000 [47]	Al (13,963), Cu (6629)	2–4
Electrical: maximum current density (A/cm^2^/g/cm^3^)	CNT/Cu (*M*_*f*_ ≈ 17%) 1.15⨯ 10^8^ [48]	Cu 5000 [49]	23,000
Thermal: thermal conductivity (WmK/g/cm^3^)	CNT, G > 700	Al 88	14
Energy storage: anode capacity (mAh/g)	Si, 3000	≈100 (Cd)	30
Other: Specific surface area (m^2^/g)	G, up to 2600	unknown	n/a

### Nanomaterials architectures: manufacture-property relations

**Fig. 6:**
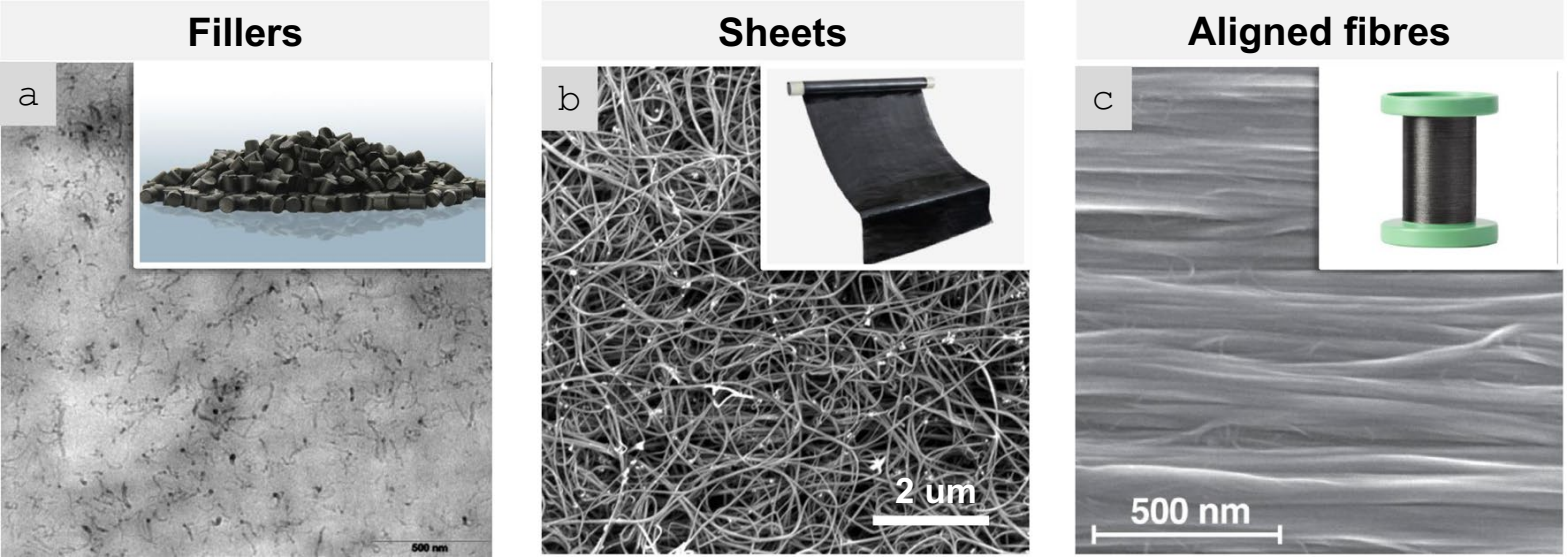
Main macroscopic architectures of nanomaterials relevant to aeronautical components: **a** Randomly dispersed fillers, typically at low volume fraction, in a polymer matrix, simple to manufacture but with low-level properties (examples: TEM micrograph of ~ 1% vol. MWCNT-composite prepared by a masterbatch dilution method [57]; inset to **a** commercial polymer masterbatch with Graphistrengh® MWCNTs by Arkema (Copyright 2024 Arkema). **b** Sheets or fabrics of high aspect ratio nanoparticles (e.g., nanotubes), which could be combined with macroscopic constituents, for example CFRP hybrids, to complement properties or enable new ones (examples: multifilament CNT fabric from the floating-catalyst CVD synthesis [58]; inset to **b** commercial non-woven sheet of CNTs by Tortech Nano Fibers Ltd. (Copyright 2017-2024 Tortech Nano Fibers Ltd.). **c** Fibres of highly aligned nanomaterials, with high volume fraction and high-performance properties (examples: magnified view of the highly-aligned CNT fibre spun from liquid crystalline solution [111]; inset to **c** commercial continuous CNT fibre by DexMat Inc. (Copyright 2018-2024 DexMat Inc.)). **a** Reproduced from [57] with permission. Copyright 2005 Taylor & Francis. **b** Adapted from [58] under the Creative Commons CC-BY license. **c** Adapted from [111] with permission. Copyright 2021 Elsevier

In order to analyse the relation between different manufacturing routes for nanomaterials and resulting bulk properties, macroscopic architectures of nanomaterials may be divided into three types: randomly dispersed fillers, sheets, and highly aligned fibres (Fig. 6). They have progressively higher alignment and higher volume fraction (here, for porous solids we take their volume fraction in air, *i.e.*, their apparent density/theoretical density).

In the first architecture, the nanoparticles are used as particles dispersed in a matrix and either individualised or with a low degree of aggregation, thus leading to a low volume fraction below ∼ 0.1 (10%). An archetypal example is a (nano)composite consisting of a polymer matrix with randomly oriented nanoparticles forming a percolated network (Fig. 6a). This strategy is effective in improving matrix properties with small amounts of filler.

Electrical conductivity, for example, can increase from a dielectric level to a low-range conductor (∼ 1 S/m) with less than 1% vol. of high-aspect ratio conductive nanoparticles. A particularly important example is nanocomposite battery electrodes, such as those composed of a “matrix” of inorganic active particles and a continuous network of aggregated nanocarbon conductors embedded inside [50].

A distinctive aspect of the filler route is that it is, in principle, compatible with industrial manufacturing routes for powder processing, such as extrusion, calendaring, shear-mixing, etc*.* This requires handling the increases in viscosity upon nanomaterial additions by keeping volume fraction close to percolation (~ 1/s), typically at a few % vol.

An alternative architecture is macroscopic sheets or fabrics of high-aspect-ratio nanoparticles aligned out of the plane but with random orientation in the plane, sometimes referred to as buckypapers and demonstrated for multiple 1D and 2D nanomaterials (CNT, G [51], MoS_2_, Si [52], SiC [53], BN, and mixtures [54]). They are analogous to a non-woven fabric or a nanotextile (Fig. 6b), typically with densities below 0.5 g/cm^3^ and volume fractions of 5–40%. They are often conveniently produced by filtration of dispersion of nanoparticles, a technique that enables combinations of different nanoparticles. For this method, the need to form a dispersion typically limits the size of the nanoparticle. However, nanoparticles of tens of micros can be processed using dilute dispersions and size selection through centrifugation [55]. A related format is sheets of 1D nanomaterials aligned perpendicular to the plane at the growth point and then mechanically shear-pressed as sheets [56].

Sheets of nanomaterials often have the right format to position them in selected locations of a hierarchical structure in order to complement the properties of a component. The most common example is nanoparticles introduced in fibre-reinforced polymers (FRPs), either between fibre filaments or in the interlaminar region. The initial focus was on providing interlaminar reinforcement and through-thickness electrical conductivity in laminate composites [59]. Further work has targeted the introduction of nanomaterials in structural composites for damage detection and other sensing applications and even for energy storage and conversion [60] in what is broadly termed structural power composites [61]. A widely explored method is to coat fibre nanoparticles from dispersion, for example, by spray coating or by impregnation of fabrics with a nanomaterial-containing resin [62]. Another strategy consists of growing nanostructured directly on the surface of reinforcing fibres, a strategy already demonstrated with CF, ceramic fibres, and multiple nanomaterials (CNT [63], ZnO [64], SiCNW [65], etc*.*).

The third type of architecture consists of continuous macroscopic fibres made up of highly aligned nanomaterials (Fig. 6c). They resemble staple yarns in their hierarchical structure and general handling [66] and high-performance fibres when made of highly aligned nanomaterials [67, 68]. Their network structure, somewhat similar to a macromolecular solid, provides intrinsic damage tolerance, piezoresistance, and order-of-magnitude higher toughness than their monolithic analogues. On the other hand, assembling nanomaterials as highly aligned fibres maximises stress and charge transfer between nanoparticles, leading to fibre longitudinal properties in the range of high-performance structural fibres and metallic conductors.

### Nanocarbon production capacity

**Fig. 7:**
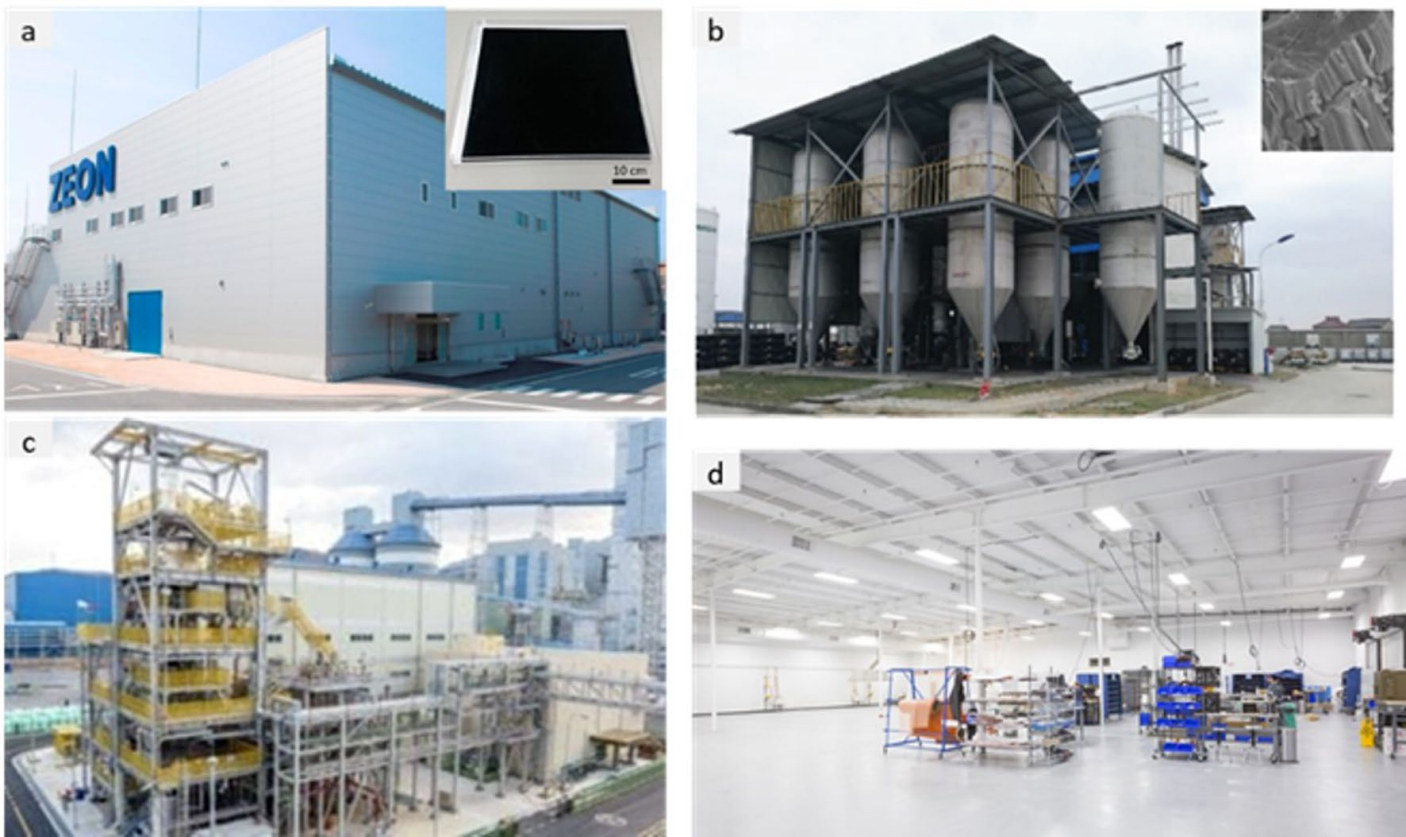
Main macroscopic architectures of nanomaterials relevant to aeronautical components: **a** Zeon Corporation plant in Japan (Copyright 2016 Zeon Corporation; https://sciencex.com/wire-news/225539173/worlds-first-super-growth-carbon-nanotube-mass-production-plant.html); **b** 1000 tonnes per year plant facilities of CNT arrays developed by Tsinghua University (China) [25]; **c** LG Chem’s Yeosu Plant (South Korea) (Copyright 2020 LG Chem; https://www.chemengonline.com/lg-chem-to-expand-carbon-nanotube-production-capacity/); and **d** new plant of Veelo Technologies (USA) (Copyright 2019 Veelo Technologies; https://www.compositesworld.com/articles/plant-tour-veelo-technologies-woodlawn-ohio-us). **b** Adapted from [25] with permission. Copyright 2018 John Wiley and Sons

Of the different high aspect ratio nanomaterials, nanocarbons are produced at the largest volumes and offer the best ground for analysis of the relation between assembly routes, production capacity and bulk properties. The capacity for production of CNTs in 2023 is in the order of ~ 5000 tonnes per year and is expected to grow annually above 10% for the coming years. Figure 7 presents examples of industrial manufacturing facilities. LG Chem (South Korea) will have a total capacity for production of 6.1 kt/yr by 2025 after setting in operation the world’s largest single-line plant (3.2 kt/yr) [69]. JEIO, another company from South Korea, expanded their CNT plant from 120 tonnes to 1000 tonnes per year in 2022 and will scale up to 6000 tonnes by 2026, targeting single-wall CNTs [70]. Korbon (also South Korea) is building up a 300 tonnes/year plant in the USA as part of the supply of SWCNTs for EV batteries to begin mass production in 2025 [71]. Chinese producers, including HXNANO (500 tonnes/year), Shenzhen Sanshun Nano New Materials (600 tonnes in 2017, acquired by Cabot in 2020), Zhongke Times NANO (700 tonnes/year MWCNT, 2 tonnes high purity SWCNT), Shenzhen Nanotech Port (200 tonnes/year) and others have a joint capacity of over 2000 tonnes/year. The largest European producers are Arkema, with a corresponding annual production capacity of 400 tonnes, and Nanocyl (460 tonnes). OCSiAl, originally Russian-founded and based with a production capacity of 60 tonnes in 2022, was authorised for commercialisation in Europe up to 100 tonnes of TUBALL nanotubes annually [72] and is about to open a 60 tonnes/year plant in Serbia in 2024, followed by another one in Luxemburg by 2027 with a production capacity of up to 100 tonnes/year. In 2019, the company opened the second TUBALL centre in Shanghai and, most recently, in partnership with Haiyi and Shenyang East, started production of the CNT dispersions for Chinese battery manufacturers [73].

The data on industrial manufacturing of CNT ensembles, such as CVD vertical arrays or sheets, are extremely limited. However, there is a notable growing trend in the industrial capacity of such structures. Q-Flo (UK) scales up the production of aligned CNTs in the United Kingdom using the floating catalyst CVD process. Veelo Technologies (USA) announced increases in the production of CNT sheets (VeeloHEAT) for integration in aerostructures as nonmetallic material for de-icing and scheduled for early-stage qualification on commercial aircraft in 2020 [74]. Nawa Technologies (France) has acquired technology to grow vertically aligned CNTs based on a two-step process developed at MIT and recently demonstrated the ability to grow vertically aligned CNTs simultaneously on both sides of a 90 cm-width substrate, thus tripling the manufacturing capacity [75]. The company, with production lines located in France and the United States, plans to become “the largest manufacturer of industrial-scaled VACNTs”, focusing on ultra-strong composites and energy storage [76]. According to the press release of 2017, Fujitsu Laboratories aimed to commercialise CNT sheet products with heat-dissipation performance for vehicles starting in 2020 [77]. Zeon Nano Technology Co. Ltd. completed and began the operation of the industrial-scale world’s first mass production plant for CNTs using the super-growth method of growing aligned SWCNT forests continuously, developed in collaboration with the National Institute of Advanced Science and Technology (AIST, Japan). The production plant is aimed at ton-scale production of long, pure, and high surface area aligned SWCNTs [78]. In China, CNano (JiangSu Cnano Technology Co., Ltd.) [79], with an exclusive license of patented technology developed by Tsinghua University (China), have extended the technology through the proprietary innovations for mass production of carbon nanotubes, whereas Tsinghua University recently developed the 1000 tonnes per year plant facilities of manufacturing CNT arrays [25].

A similar trend is observed in graphene production as a raw additive and its integration into semi-finished products. The production capacities of graphene powders increased significantly compared to the 2014 level [80]: Angstron Materials (USA, up to 1200 tonnes/year in 2015), Sixth Element Materials (China, 1000 tonnes/year in 2020), Ningbo Morsh (China, 500 tonnes/year in 2015), Deyang Carbonene Tech (China, 300 tonnes/year 2020). NanoXplore Inc. (Montreal, Canada) completed the commissioning of a fully automated production plant with 4000 tonnes/year capacity [81] with interests in battery initiatives and supplying graphene powders for the North American automotive market [82]. In 2018, China was considered to have ~ 75% of the global production capacity for graphene [83], with the ability to produce 2000 tonnes/year of graphene powders and 3.5 million m^2^ per year of graphene films [84], and its capacity is further expanding. With a total investment of > 500 million Euros in building the new graphene industrial park [85], the Hengli Sheng Tai Graphene Technology Co. Ltd was expected to reach 5000 tonnes per year by 2020 [86]. Large technological companies have stimulated graphene production for batteries and electronic applications. Huawei (China) recently launched a new investment in heat dissipation management in addition to the company’s existing products based on graphene [87]. General Graphene company (USA) commissioned the latest production line with a capacity exceeding 100,000 m^2^ of CVD graphene per year [88]. LG Electronics (South Korea) developed their own roll-to-roll CVD graphene technology for large-area and high-quality graphene production almost a decade ago, now claims to be a worldwide supplier of monolayer graphene with a production capacity of about 30,000 m^2^/year [89, 90]. The Samsung Group (South Korea), one of the leading companies in graphene-related patents worldwide [91], announced it is close to using graphene in batteries for their smartphones with five times faster charging speed than standard lithium-ion ones [92, 93], based on their recent development of the CVD growth of the hierarchical three-dimensional graphene-silica assembly with the silicon oxide nanoparticles (“graphene-balls”) [94].

Aligned CNT fibres are currently at the semi-industrial manufacturing stage and undergoing scale-up, with limited data on production capacity. As a rough estimate, CNT fibre manufacturing capacity has been under 0.1 tonnes per year, although the FCCVD process is envisaged to be suitable for scale up to about 150 tonnes/year [95]. A decade ago, NanoComp Technologies (USA) announced that their production of yarns and non-woven textiles would reach about 4 tonnes by 2013 [96]. In 2018, it was acquired by Huntsman Corporation (USA) [97], which continues to further scale up manufacturing capabilities of high-strength CNT yarn/tapes [98]. In 2023, Huntsman Corporation commenced construction of the 30 tonnes/year MIRALON® carbon nanotube materials plant using FCCVD synthesis [99], and Dexmat (USA) has reported a 20 × production capacity increase to meet the market demand for Galvorn® CNT materials produced by LC spinning [100].

In China, Suzhou Innovation Nanocarbon Co. Ltd. is a leading manufacturer of continuous CNT materials by FCCVD, yet its annual production capacity is unknown [1]. In 2018, Shenzhen Cone Technology Co. Ltd. started operating a plant to produce 30,000 tonnes of CNTs in the form of paste [101], and one year later, implemented the second generation mass production equipment for manufacturing CNT fibres and textiles based on the aligned CVD-arrays at the “hundred-tonnes-level” [102]. There is no data on industrial manufacturing of continuous graphene fibres.

Table 2 provides the main features of the different architectures for introducing carbon nanotube-based materials in engineering components. It includes relevant properties (discussed below), the different processing routes to integrate nanomaterials, and estimates of the established production capacity of nanomaterials for the different architectures based on the (limited) industrial information available. As a first aspect, it highlights the importance of nanoparticle assembly on macroscopic properties. The different assembly routes lead to the electrical conductivity values ranging from 10^–6^—10^2^ S/m for fillers, to 1–10^2^ S/m in hybrids with CF and 10^3^–10^5^ S/m for sheets of different alignment, to 10^5^—10^7^ S/m for aligned fibres. Another corollary from this analysis is the significant mismatch between production capacity and bulk property level. Significant capacity exists only for fillers. Furthermore, although sheets of hybrids and fibres can be produced from powder nanomaterials, such as CNTs or G, they typically require much higher purity and crystallinity than the scaled-up filler grade.

**Table 2:** Examples of high-aspect ratio carbon nanotube-based materials used in different architectures, with associated processing routes and component characteristics*

Architecture	World production (tonnes/year)	Method to process into macroscopic object	Volume fraction in component	Notable macroscopic property
Fillers	Industrial, ≈ 3000–5000^a^	Dispersed in matrix (extrusion, calendering, shear-mixing)	Close to percolation, around 1/s Typically, < 1%	Electrical conductivity of polymer nanocomposite 10^–6^—10^2^ S/m
Sheets	Semi-industrial to Industrial, Less than 10^b^	Filtration from dispersion; Direct CVD on fibre filaments; Drawing from CVD arrays; Direct spinning at synthesis	Above percolation threshold (< 5% in total), (10–40% local)	In-plane electrical conductivity 10^3^–10^5^ S/m; Through thickness electrical conductivity 10^–2^—10^0^ S/m; Increase in interlaminar reinforcement
Aligned fibres	Semi-industrial, Less than 0.01^c^	Direct spinning at synthesis; Spinning from dispersions/solutions; Spinning from CVD arrays	Close to closed-packing, Typically, > 30%	Electrical conductivity 10^5^—10^7^ S/m; Tensile strength 2–6 N/tex; Tensile fracture energy 30–100 J/g

*Properties may differ for scaled up materials for which capacity is estimated

^a^Information from publicly announced capacity for European, US and Asian suppliers

^b^Estimated for a semi-industrial facility for CVD growth of aligned CNTs

^c^Estimated for a semi-industrial facility for FCCVD growth of aligned CNTs

## Electrical conductors

### Progress on nanostructured conductors

**Fig. 8:**
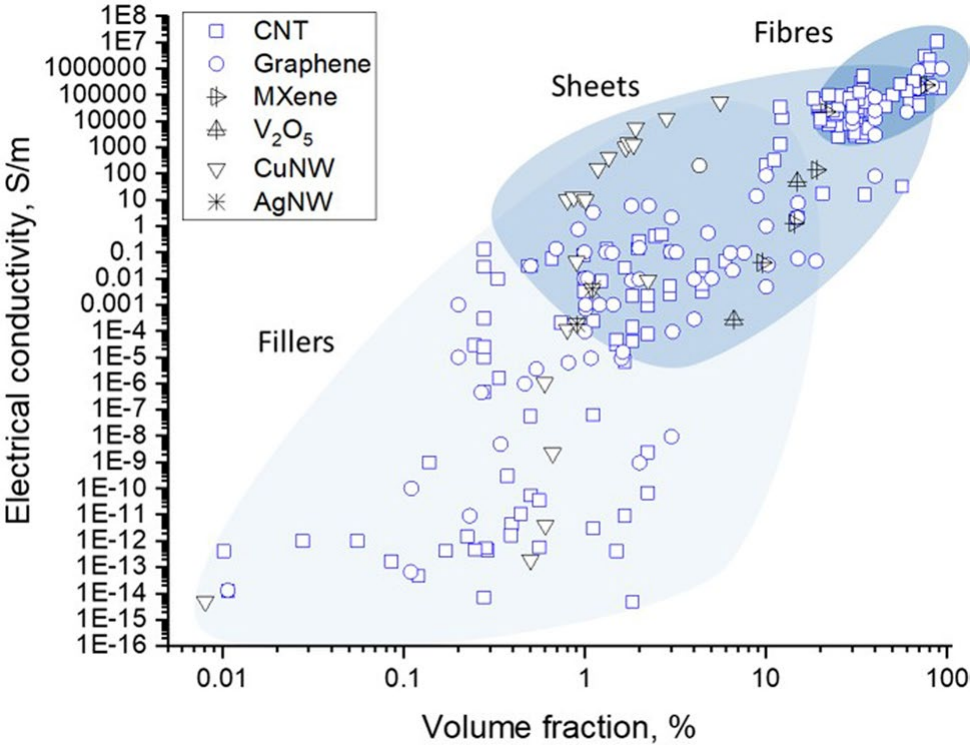
Electrical conductivity of nanocomposites with randomly dispersed nanoparticles, and their macroscopic ensembles (sheets and fibres). Copyright 2024 IMDEA Materials

In nanocomposites with nanosized conductors, bulk electrical conductivity develops through the formation of a continuous network of conductors in contact spanning from one end of the sample to the other. Figure 8 shows an example of literature data for different nanomaterials. It shows that more than ten orders of magnitude for electrical conductivity are attainable, spanning the range from conductive polymer nanocomposites to metals, depending primarily on the final volume fraction and thus dependent on the form of integration.

For isotropic networks of fillers, the dependence of bulk electrical conductivity (σ) on volume fraction (V_f_) can be accurately described by percolation theory, according to:

1 $$\sigma = {\sigma }_{0}({V}_{f}-{V}_{fc}{)}^{t}$$

where σ_0_ is the limiting conductivity, t is an exponent between 1–3 depending on the dimensionality of the filler, and V_fc_ is the critical volume fraction for percolation. V_fc_ is of the order of 1/s, highlighting the interest in high-aspect-ratio 1D nanomaterials such as CNT, metallic NWs, and few-layer 2D nanomaterials. With nanocarbons, nanocomposite conductivities of the order of 1 S/m may be achieved at volume fractions below 10%, which may be sufficient for some applications such as inks, conductive polymer matrices and nanocomposite electrodes. It is also interesting to explore the upper limit for such composites. (Note that for ${V}_{f}\gg {V}_{fc}$,$\sigma \approx {\sigma }_{0}{V}_{v}^{t}\approx 0.1{\sigma }_{0}$). Different nanocarbons show limiting conductivities around 10^2^ S/m. Nanocomposites with metallic NWs may lead to higher values of σ_0_. However, an accurate estimate is lacking because most embodiments are in the form of transparent planar networks of very low V_f_. Nevertheless, 3D random networks of MNWs would have a significant contribution from NW junction resistance [103] and thus fall orders of magnitude below the conductivity levels of bulk metals. The implication is that for high-performance conducting applications, different forms of nanomaterial organisation are required.

For macroscopic sheets or fabrics of nanocarbons with different degrees of in-plane alignment conductivities are typically in the range of 10^3^–10^5^ S/m [104–109]. The conductivity in the out-of-plane (through-thickness) direction is a few orders of magnitude lower; values of 0.06 S/m [108] and above 1 S/m [110] are reported for non-woven CNT fabrics produced by FCCVD.

A separate class of nanostructured conductors are fibres of highly aligned nanocarbons analogous to CNT wires. When appropriately synthesised, assembled and doped, nanocarbons are attractive conductors because of their combined electrical figures of merit, low density, and high thermal/chemical stability. Their longitudinal conductivity is typically around 10^5^ S/m and as high as 10^7^ S/m after the addition of other compounds. Conductors based on CNT fibres are particularly relevant for the transport sector. Together with superconductors, they are the only existing types of materials that can potentially match the overall demand for conducting wires for aerospace in the following decades [17]. Given their importance, they are reviewed in more detail below.

**Fig. 9:**
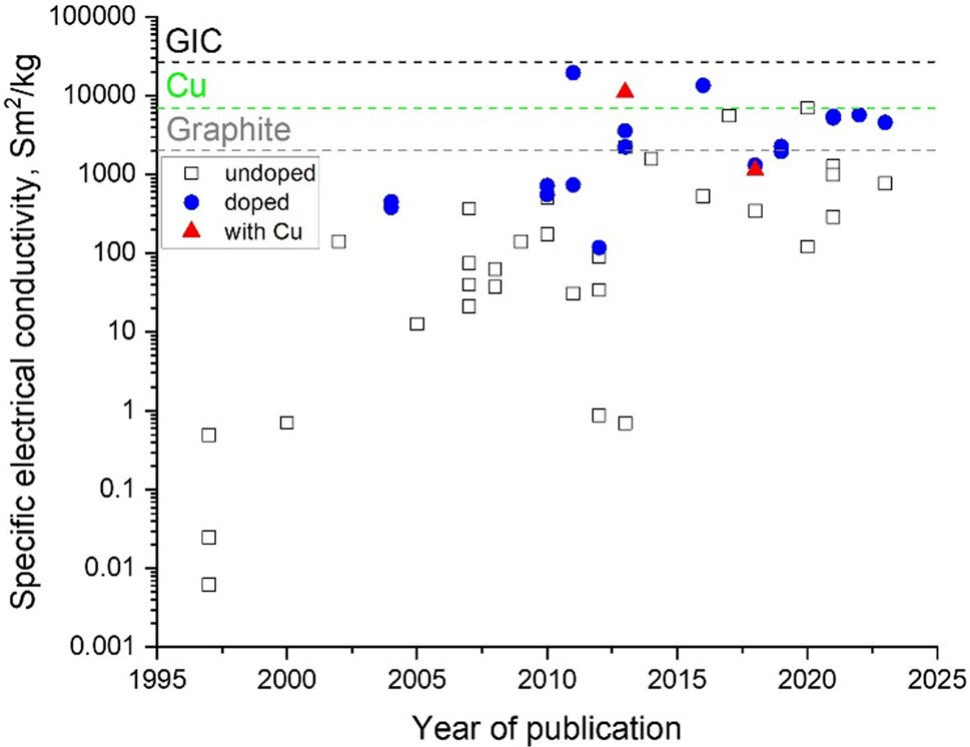
Improvements in specific electrical conductivity of continuous nanocarbon fibres achieved through structural improvements, introduction of dopants and hybridisation with metals. Copyright 2024 IMDEA Materials

Demanding electrical properties require nanocarbons with very high crystallinity and purity assembled into closed-packed structures extending in the form of cables. Long, highly crystalline building blocks reduce scattering from defects that translate into higher electrical mobility, therefore electrical conductivity, while also increasing thermal conductivity and thermal stability, thus enabling higher ampacity. Alignment reduces the contribution from resistance between nanocarbons in the network. As these improvements have been implemented in fibre synthesis processes, they have translated into a constant increase in the electrical conductivity of nanocarbon cables. As shown in Fig. 9, the conductivity of all the different types of nanocarbon fibres increased after controls of the synthesis process. Consistent with this trend, historical data for CNT fibres produced by wet-spinning from liquid crystalline solutions indicate a 26% increase in electrical conductivity per year [111].

**Fig. 10:**
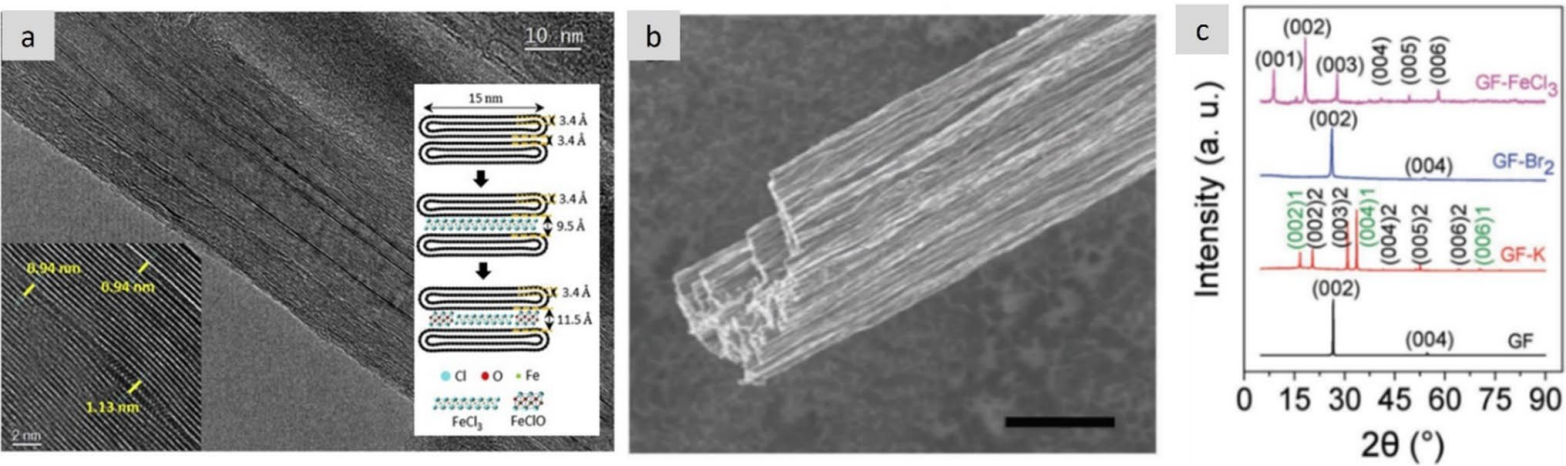
Examples of continuous fibres of intercalated nanocarbons with high electrical conductivity. **a** FeCl3-intercalated bundles of CNT in a macroscopic yarn [116]. **b** Fibres produced from graphene intercalated with different guest compounds [114]. **c** XRD patterns of the pure and doped graphene fibres. **a** Adapted from [116] with permission. Copyright 2021 Elsevier. **b**, **c** Adapted from [114] with permission. Copyright 2016 John Wiley and Sons

The second source of improvements has come from doping methods, whereby an additional material integrated into the nanocarbon increases the electrical conductivity of the supporting nanocarbon network. The dopant is introduced through non-covalent methods, thus introducing charge carriers into the nanocarbons, but without introducing defects that reduce charge mobility. Examples include adsorbed species [112] or intercalant [113], often introduced as a vapour. Doped fibres of either CNTs or graphene with higher specific conductivity than copper have been produced after the introduction of dopants [114]. The methods are scalable, and some are already used in commercial CNT fibre materials.

, There is room for improvement in intercalated materials. Basal plane conductivity of graphite intercalated with AsF_5_ has reached 63 × 10^6^ S/m [47], with a density around 2.3 g/cm^3^, which is just above Cu on a volume basis but an order of magnitude superior on a mass basis (Fig. 9). Nanocarbon conductors offer a molecular structure suitable to realise these values over macroscopic distances (Fig. 10), with the potential to combine intercalation and ballistic transport. A drawback of intercalation/doping methods is the relatively low stability of the intercalant, which can sublime at moderate temperatures or degrade over time [115]; however, recent studies have shown unusually higher stability in intercalated nanocarbons [116–118] compared to traditional graphite intercalation compounds (GIC).

**Fig. 11:**
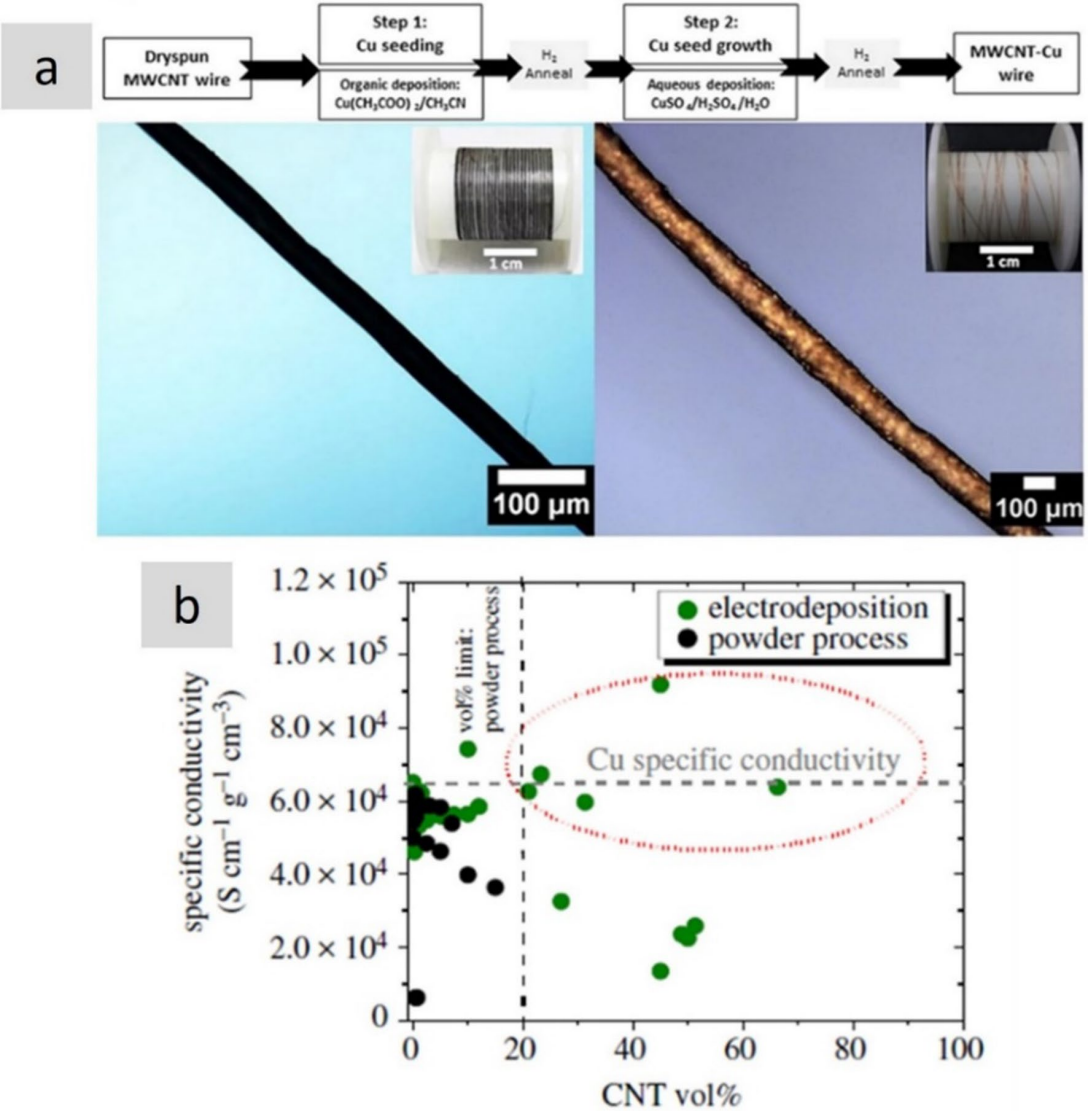
Cu/Nanocarbon fibre hybrids. **a** Example of a hybrid continuous fibre of CNT/Cu with superior specific electrical conductivity and specific ampacity than pure Cu [49] and **b** literature data showing specific conductivity values above Cu for aligned CNTs with electrodeposited Cu [119]. **a** Adapted from [49] under the Creative Commons CC-BY license. **b** Adapted from [119] under the Creative Commons CC-BY license

Another strategy is producing hybrids of nanocarbons and highly-conducting metals [119]. These hybrid materials are typically produced by electroplating the metal onto the nanocarbon scaffold. This technique has been demonstrated to allow for significant control over hybrid morphology and volume fraction (Fig. 11). Specific conductivity values higher than for pure metal have been reported for Cu/CNT fibre materials, for example. Very importantly, these hybrids sustain high current densities beyond a simple rule of mixtures. This effect is attributed to reduced electromigration of the metallic material inside the nanocarbon network, although a charge transfer process between the two phases has also been proposed [120], suggesting that the different contributions to conduction in these hybrids remain unclear and have room for improvement.

### General trends in the use of conductors in civil aircraft

At present, most of the operating empty weight (OEW) of current aircraft is from structural elements, such as fuselage, wings and horizontal and vertical tail planes, which account for about 68%wt. However, the systems and fixed equipment also involve an important weight share, representing around 25%wt. However, whereas structural elements are expected to remain at a nearly approximate weight, electrical conductors used in systems, equipment, propulsion, and other applications will see the most significant increases in future aircraft.

**Fig. 12:**
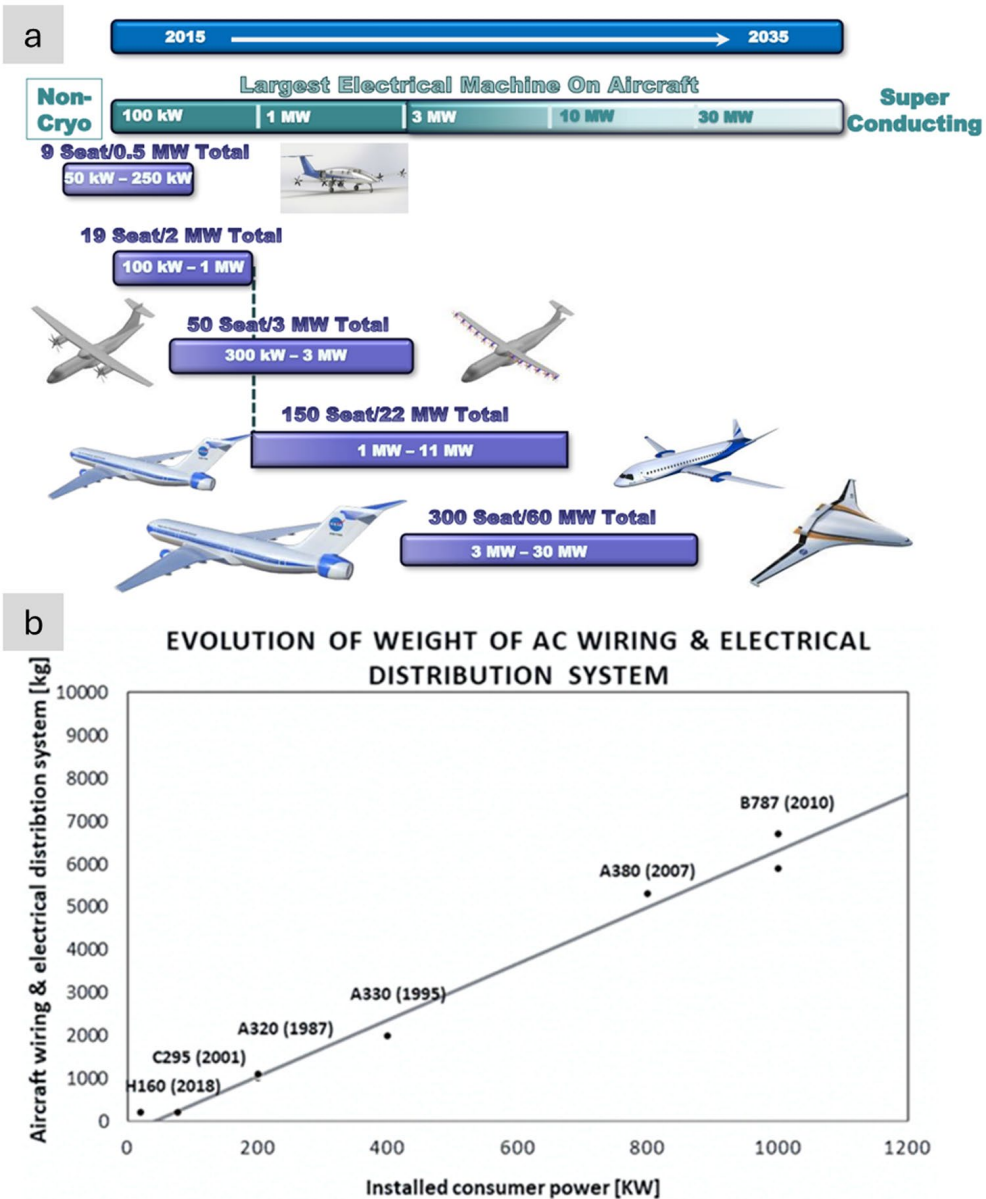
Increasing need for conductors in emerging aircraft. **a** Evolution of required electrical power in civil aircraft [123] and **b** Historical data showing a linear increase in weight from electrical wiring and distribution/interconnection systems with installed consumer power. Copyright 2017 NASA, Work of the US Gov

A wide-body aircraft has an estimated 500 kms of cables, weighing roughly 7.5 tonnes and contributing about 3% of the aircraft’s weight [121]. This weight may seem modest; however, the key is to consider projected requirements and the rapidly increasing need for additional wiring to match the vertiginous increase in electrification. According to estimates by NASA [17] and others [122], the power delivered by the largest electrical machine on aircraft will increase by a factor of 30 in the next 15 years (Fig. 12a). Historical data suggests a nearly linear increase of 6.5 kgs of aircraft wiring per 1 kW of installed consumer power (Fig. 12b), and a concomitant linear increase in recurrent costs. This implies that without introducing new materials, the projected 30 MW of electric power required before 2035 would translate into nearly 200 tonnes of wiring: about the same as five short-range aircraft (e.g., Airbus A320, Boeing 737)! The pressing need for radically new, lighter conductors to realise future aircraft is evident.

**Fig. 13:**
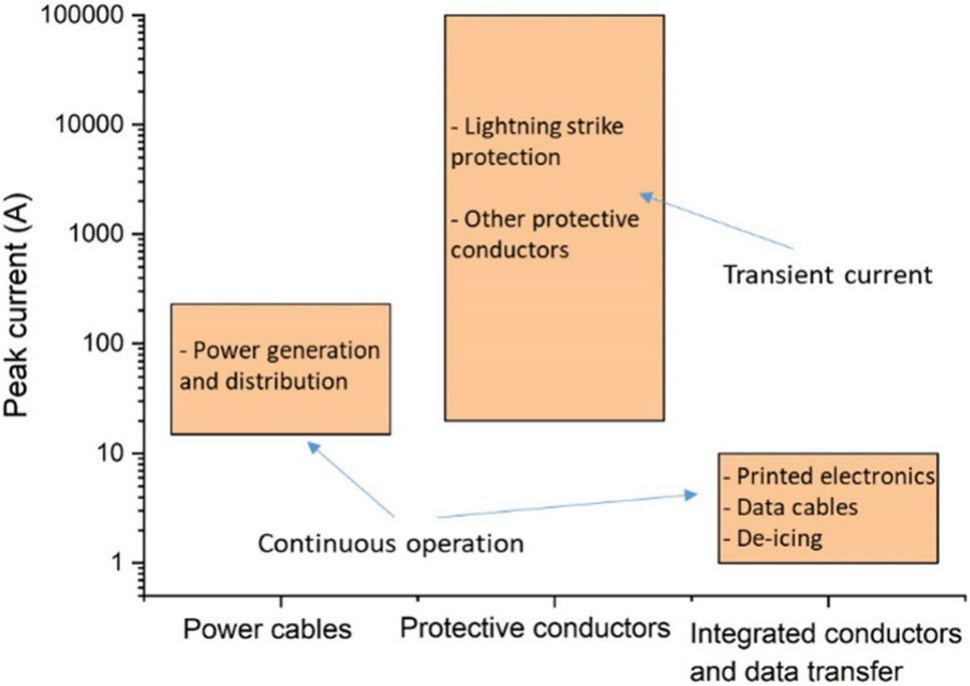
Aircraft conductors grouped in different classes in terms of maximum peak current sustained and other dominant properties: power cables, protective conductors, and integrated conductors and data transfer. Copyright 2024 IMDEA Materials

To guide the discussion, we divide conductors for aerospace into three categories: power cables, protective conductors, integrated conductors, and data transmission. This separation helps identify the main performance indicators of current nanomaterials and the directions for improvement. As entry properties, power cables require high specific conductivity and specific maximum current density (specific ampacity); protective conductors are not designed for continuous current flow and instead need predominantly high specific ampacity; integrated conductors, for example, for printed electronics or embedded cabling require a combination of specific electrical conductivity, high specific electromagnetic absorption, and general mechanical toughness. This qualitative separation of conductors based on maximum currents sustained is shown in Fig. 13. Details of precise performance metrics and an overview of extended materials requirements for each class are discussed in more detail below.

### Power cables

#### Operating conditions and requirements of present and future power cables

The power distribution in modern aircraft follows a complex network with multiple cable routes that have different requirements and functions. They can be broadly divided into different types based on their operating conditions in terms of voltage and current. Aircraft have traditionally used 28 V (DC—Direct Current) for power distribution, but the last-generation aircraft already use high voltage distribution of 270 V (DC). Moreover, the first all-electric aircraft uses voltages of up to about 500 V (DC). It is envisaged that megawatt-class electrified-propulsion systems for single-aisle airliners operate at up to 3000 V (DC). Similarly, at present, power cables work at DC currents of 15- 35A, but realising MEA and electrical propulsion require currents as high as 200 A.

There are multiple property and safety requirements on power cables for aircraft that affect not only the choice of conducting material but also the insulating layer and the overall shape and size of the entire cable. Some examples are included in references [124]. In general, the cable selection and design in an aircraft must consider the following key factors: line voltage drop, operating current, external and internal cable temperature, and minimum short-circuit current, allowing the protection of the device from tripping and connections.

Reducing the line voltage drop requires essentially minimising electrical resistance per unit cross-section or unit weight, *i.e.*, using a material with as high specific conductivity as possible. Similarly, most requirements concerning operating current are related to the stability of the cable at high current densities. With increased current density, the temperature of cables increases rapidly as a consequence of Joule heating.

Significant temperature increases in cables are avoided for multiple reasons. Most evidently, they can cause damage to neighbouring materials. Hence, design provisions ensure that the temperature increase at a cable bundle skin level does not exceed 40ºC. Internally, the cables may be much hotter. The maximum permissible temperature of the insulation is 180ºC for Al conductors and 260ºC for Cu conductors. Thermal requirements for aeronautical cables are almost as high as those for electrical ones. Small changes of 15 °C can force the choice of a much heavier cable with a higher ampacity [125]. Cable morphology is relevant, as it affects heat dissipation. However, at a materials level, essential properties are the electrical and thermal conductivities above room temperature, specific ampacity and thermal coefficient of resistance (including when embedded in a polymer matrix [126]). Ampacity is not an intrinsic physical property; it depends on specific heat capacity, thermal stability, and other parameters. It is challenging to determine ampacity analytically from known material properties, but it is relatively easy to measure experimentally. Therefore, we adopt it as a pseudo property of materials.

Aeronautical cables consist of a metallic conductor and a polymeric insulating sheath. The conducting core is made of Cu, Al, or a Cu/Al alloy. The core is plated with a 1-micron thick layer, most often of Ni, but alternatively of Ag or Sn. Its main purpose is corrosion resistance, although Ni coatings can also provide thermal protection. (A comparison of the properties of metallic power cables and cables of nanostructured materials is presented in Table 3).

**Table 3:** Figures of merit for power cables of CNT fibres, Cu or Al [127]

Constituent material	A1 (A mm^3/2^)—volume normalised	A2 (A (mm/g)^3/4^)—mass normalised
CNT fibre	3	756
Cu	80	3293
Al	59.3	6000

#### Nanostructured power cable prototypes

**Fig. 14:**
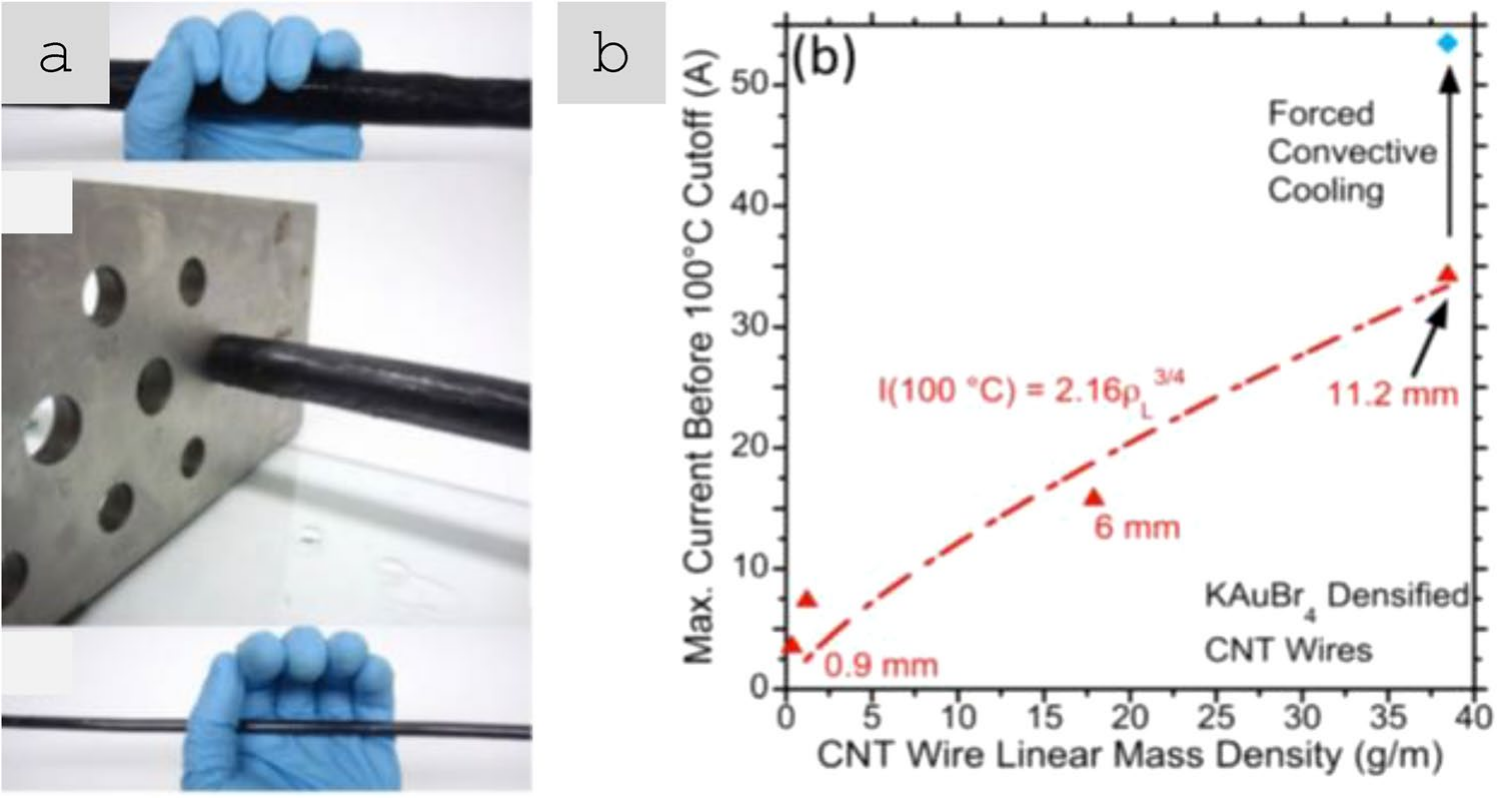
Power conductor of CNT fibres. **a** Fabrication by dye densification of commercial sheets of CNTs. Diameter decreases from 22.5 mm to the final 6 mm diameter after densification. **b** Scaling law for maximum current and linear density [127]. **a**, **b** Adapted from [127] with permission. Copyright 2017 AIP Publishing

Most properties of nanostructured conductors measured in research laboratories are large enough to exhibit accurate bulk properties of the material but small compared to engineering components. However, significant progress has been made in the fabrication and testing of electrical components using CNT fibre conductors according to application-based conditions (Fig. 14). Cress et al*.* produced 1 cm-diameter cables from commercial sheets of CNTs produced by Nanocomp [127]. The cables could operate at 20 A and failed above 45 A for cables doped with KAuBr_4_ through oxidative degradation above 500 °C.

The work compared performance relative to Cu an Al on a volume basis, measured in terms of 100 °C limiting current (I) and once cable diameter (d) was factored in ($I={A}_{1}{d}^\frac{3}{2}$). The proportionality constant A1 can be seen as a figure of merit of maximum current for a given cable diameter (though with units of Amp mm^3/2^). Alternatively, differences in volumetric density can be taken into account using:

2 $${A}_{2}={A}_{1}{\left(\frac{4}{\pi {\rho }_{v}}\right)}^\frac{3}{4}$$

where ρV is the volumetric density.

A comparison in Table 3 shows that the performance of these CNT fibre cables is within factors 4 and 8 of commercial Cu and Al cables, respectively. Note that the constituent CNT material used for such study had a longitudinal conductivity of around 1.3 × 10^6^ S/m, whereas recent reports have demonstrated 10.9 × 10^6^ S/m [111]. This strongly indicates that a power cable made up of present CNT fibre materials would outperform Cu and Al under similar prototype-scale tests.

### Protective conductors

Protective conductors are specific to aircraft with fuselage predominantly of composite materials. The transfer from previous metallic aircraft to last-generation composite ones, although involving a significant weight saving, also led to some key constraints in terms of lightning strike and electrical conductivity protection. This resulted in the design and application of add-on metallic solutions, which in some cases can even compromise the weight benefit associated with the use of composite materials in aeroplanes.

Under “protective conductors”, we consider conductive elements whose main purpose is to dissipate current, such as for lightning strike protection (LSP). This protection is provided by a metallic electrical mesh, which essentially acts as a conductive network to safely dissipate unintended current throughout the aircraft structure, although it carries additional secondary functions.

#### Lightning strike protection of structures

**Fig. 15:**
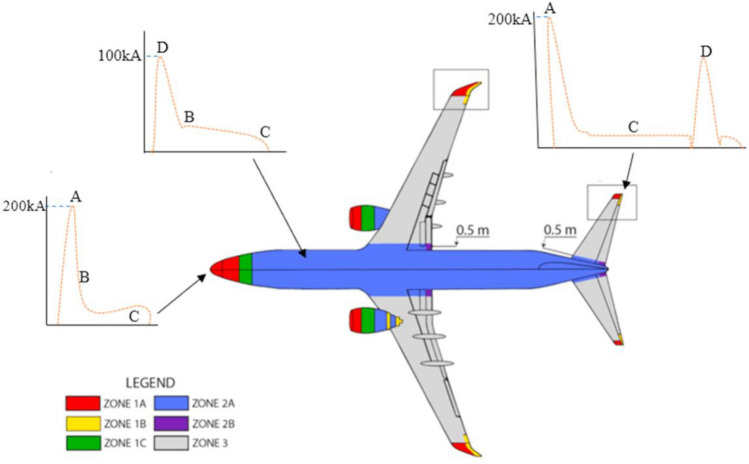
Schematic with LSP zoning in conventional commercial aircraft . Reproduced from  [128] under the Creative Commons BY-NC license

The direct effects of lightning strikes on aeronautical composite structures are the structural damages caused by the electro-thermal and mechanical forces produced by the high current of a lightning strike. Different threat areas in aircraft are defined according to the probability of a lightning strike. An example of the mapping of lightning strike zones in the last generation commercial aircraft is shown in Fig. 15: Zone 1 with a high probability of initial lightning flash attachment; Zone 2, where a lightning flash can sweep from the point of its initial attachment; Zone 3 with a low probability of a direct attachment, however, may carry substantial currents by direct conduction between two attachment points [128].

For certification, LSP is designed according to the progression of current peaks of different intensities and durations. Protective conductors for different zones are chosen based on requirements extracted from such a current profile, taking into account both maximum currents and integrated current peaks. In areas of most probable lightning strike, for example, design parameters include sustaining a current peak of 200 kA reached in less than 20 μs and 200 A for longer periods of up to 1 s.

The indirect effects of lightning strikes refer to the induced voltages in the systems due to lightning currents. During a lightning strike, the current flows through the fuselage; therefore, to limit the overvoltage in the systems, the electrical resistance of the fuselage must be kept low.

#### Edge glow

During some lightning strike lab tests of composite coupons made of last-generation aeronautical thermoset interleaved matrix, some sparking was observed in the coupon edges, a phenomenon termed edge glow. The current understanding is that during the lightning strike, a large electric potential is established between the outer and inner CFRP plies, which are separated by an insulating polymer interleaf layer. This could lead to sparks between plies at the edge, which must be avoided, particularly in the wing box, due to potential ignition risk in the fuel tank [129]. Eliminating edge glow effects requires increasing the conductivity of the composite laminates through their thickness to values around 2 S/m to match current solutions based on a surface Cu mesh (Table 4). Moreover, an improvement to 20 S/m could eliminate the need for metallic mesh for edge glow altogether. These levels are within reach upon the addition of conductive nanomaterials to polymer matrices. Indeed, industrial suppliers of aerospace-grade polymers offer commercial products to avoid edge glow consisting of polymer matrices with conductive nanoparticles.

**Table 4:** Overview of protective conductors for aircraft

Protective conductor—function	Current material/solution	Relevant materials specification	Dominant properties
Lightning strike protection (direct and indirect effects)	Expanded copper foil (175 g/m^2^) or bronze mesh, preimpregnated with a polymer	Composite skin surface resistance of 1–12 mΩ/square	Specific ampacity and specific conductivity
Lightning strike protection—Edge glow	High grade of expanded copper foil (815 g/m^2^) to ensure contact with fastener and z-conductivity	Through-thickness (Z) conductivity of the composite part	Through-thickness conductivity of composite part
		SoA > 2 S/m	
		Target performance > 20 S/m	

For a mid-size aircraft, the additional metallic elements added for LSP can amount to 5–10% of the fuselage weight. The current materials and dominant properties are summarised in Table 4. Although technical documents and common perception identify specific room temperature conductivity as the figure of merit for protective conductors for aircraft, this is an erroneous oversimplification. For one, it cannot explain the fact that current materials consist of Cu instead of Al, such as expanded copper foil or bronze mesh impregnated in epoxy resin/adhesive film. Although cost and galvanostatic corrosion are important considerations, the most likely reason is the much lower thermal stability of Al (it melts at nearly half the temperature of Cu), which limits its performance at high current densities. A suitable figure of merit would seem to be specific ampacity.

**Fig. 16:**
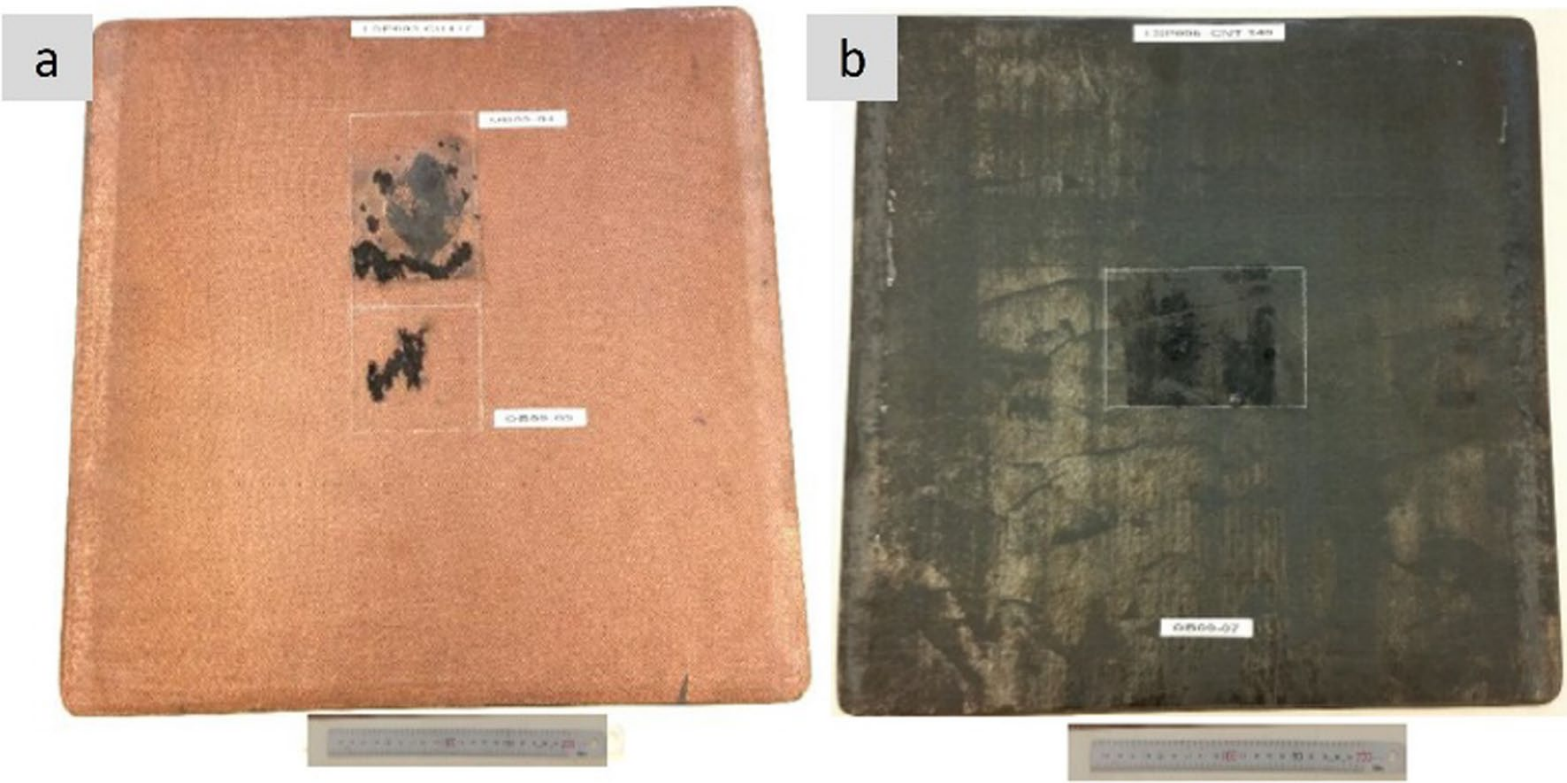
Lightning strike protection test [0]_8_ woven carbon fabric laminate with **a** Cu foil and **b** CNT fibre veils, performed at low energy strike conditions (100 kA) according to EUROCAE ED84 [132]. Copyright 2024 IMDEA Materials

Regarding lighting strike protection, several prototypes and studies show that nanocarbon-based materials can be competitive compared to standard copper foil systems. One of the solutions is using continuous macroscopic ensembles of carbon nanotubes, such as veils or sheets. Figure 16 shows examples of structural laminate CFRP composite panels with SoA Cu foil and CNT fibre veil after lightning strike tests. Under simulated LSP at 100 kA (ERUOCAE ED84), the total damaged area decreased linearly with the areal density of the protective conductor but at a much faster rate for sheets of CNTs. Regarding edge-glow protection, nanocarbon-based materials can increase the trough-thickness conductivity of the composite parts. In one example, out-of-plane conductivity increased from ~ 10 S/m for the pristine CFRP with plain woven T300 carbon fabric to ~ 16 S/m for the hybrid CFRP laminate interleaved with randomly oriented MWCNT buckypapers (at 5.1%vol CNT content) and reached 50 S/m at much higher CNT content [109]. Epoxy composites with ~ 30%vol CNT fibres produced by FCCVD have shown through-thickness electrical conductivity in the range of 40–80 S/m, depending on the porosity [130]. A significant increase in the through-thickness conductivity of unidirectional CFRP laminates from 4 S/m (control CFRP) to 21 S/m was demonstrated by interleaving it with FCCVD-produced CNT sheets at 8%wt global CNT content in the laminates [131].

#### Compendium of properties for high-current conductors

From a review of the engineering requirements on power cables and protective conductors discussed before, we identify the following dominant material properties: specific conductivity, specific ampacity, temperature coefficient of resistance, and specific thermal conductivity. Table 5 presents these properties measured on nanostructured conductors of macroscopic dimensions, compared against current materials used in aerospace.

**Table 5:** Selected properties of nanocarbon fibres and metallic materials for aircraft power & protective conductors at room temperature

Property normalised by density	Specific electrical conductivity at room temperature (S m^2^/kg)	Specific maximum current density (A/cm^2^/g/cm^3^)	Specific thermal conductivity (mWm^2^/K∙kg)	Temperature coefficient of resistance (1/K)	Thermal stability (temperature of phase change C)
CNT fibre (FCCVD)	400 [133] –530 [134]	1200	180–1220 [133]	− 0.001–0.002 [135]	> ≈ 3000^c^
CNT fibre (LC-spun)	5450 [111]	15,000 [136]	195 [111]		> ≈ 3000^c^
CNT fibre (wet-spun)	250 [137]		12 [137]		> ≈ 3000^c^
Graphene fibre (wet-spun, annealed at 2850 °C)	13.8 × 10^4 a^ OR 118 [138]		690 [138]		> ≈ 3000^c^
CNT fibre/Cu (CNT fraction 44% wt. [49], 16.3%wt [139])	1200 [49]^b^	10,000 [49], 42,400 [139]	-	1.7 × 10^–3^ [49]	1085 (Cu)
	5222 [139]			1.14 × 10^–3^ [139]	
Cu	6600	5000 [49]- 10,900 [139]	43	3.9 × 10^–3^	1085
Al			87	4.3 × 10^–3^	660

^a^Doped with K [114]

^b^Sample with full filling and density of 5.4 g/cm^3^

^c^Oxidation may start at T around 600 °C, depending on degree of graphitisation

### Integrated conductors and data transfer

The third class of conductors have in common a relatively low operating current in the range of mA—15A. They include many different components, but common examples are miscellaneous wires for the power supply of small equipment and various types of data transfer cables. They form part of avionics systems and increasingly for electronic functions in the cabin. The interest is not only in decreasing the weight associated with these wires but also in simplifying their integration methods and reducing the need for inspection and maintenance.

Opportunities for improvement of aircraft wiring are identified by considering the general example of coaxial cables. Core conductors for data transfer require several properties beyond electrical conductivity, starting with performance at multiple frequencies. An opportunity for improvement is the metallic sheath of coaxial cables, whose main purpose is to provide EMI shielding from external sources to the aircraft and to reduce interference between onboard equipment. Wiring EMI protection in aircraft depends on the type of cable, signal level and aircraft area. Shielding sheaths for coaxial cables are of Cu or Al due to their high specific conductivity. For communications, electromagnetic attenuation is a relevant figure of merit, particularly at high frequencies (40 kHz–100 GHz). Since at such high frequencies, most conduction is through the skin of the metal, there is a drive to make high surface area conductors, a strategy that lends itself to using nanostructured conductors.

**Fig. 17:**
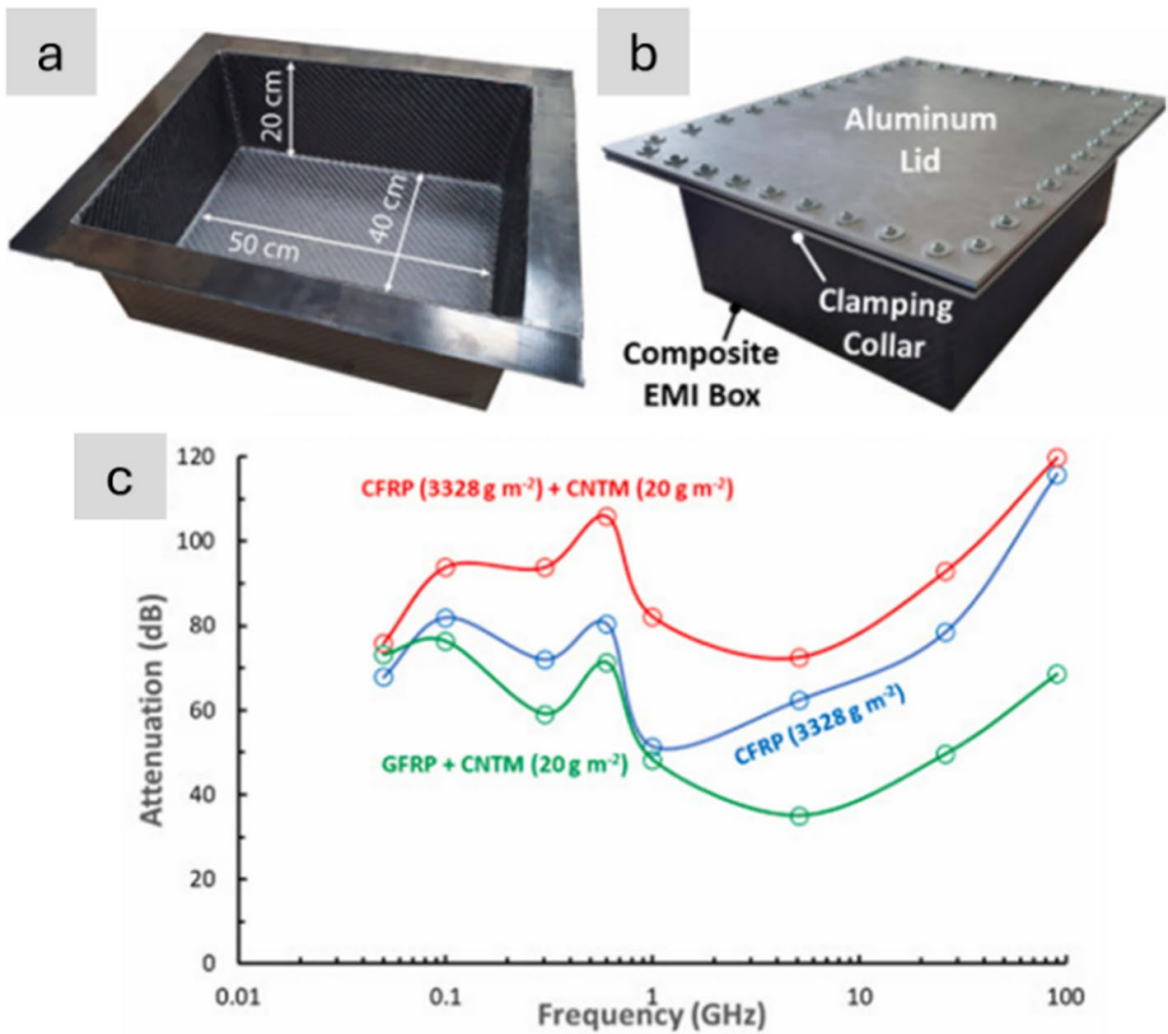
**a**, **b** Images of the CFRP boxes with CNT material EMI protection, post autoclave moulding; **c** EMI attenuation as a function of frequency fortested EMI boxes [140]. **a**–**c** Adapted from [140] under the Creative Commons CC-BY license

**Fig. 18:**
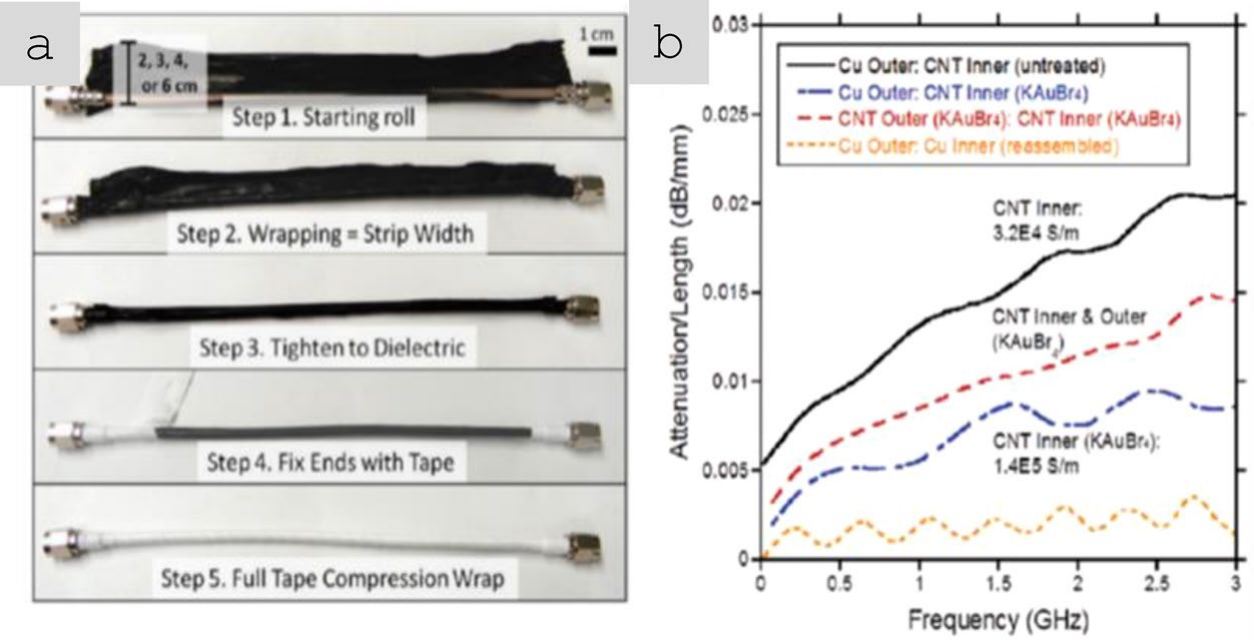
Co-axial cables from CNT fibres. **a** Fabrication of a coaxial cable with core and sheath of CNT fibre sheets. **b** Attenuation level showing improve performance when using doped CNT material [142].  **a**, **b** Adapted from [142] with permissions. Copyright 2012 American Chemical Society

Figure 17 shows the recent study of applying CNT sheets produced at scaled-up facilities for EMI shielding applications. The CNT-based gaskets outperformed a commercially available high-performance EMI gasket material based on a silicone matrix with Ni and graphite fillers at 26.5 GHz. When testing large-scale EMI CNT-based composite boxes at a frequency range of 50 MHz–90 GHz, no apparent EMI leakage was detected, and the boxes showed promising EMI attenuation capabilities up to 115.9 dB [140]. Evers et al*.* demonstrated the EMI shielding effectiveness of composite laminates reinforced with high-performance CNT yarns. In their work, EMI shielding effectiveness ranged from 90 to 120 dB measured for the 4–26.5 GHz range, outperforming CFRP of similar thickness reinforced with IM7 carbon fibres due to the higher electrical conductivities of CNT yarns [141]. Other advanced demonstrators are lightweight coaxial cables from carbon nanotube conductors. Landi et al*.* fabricated a large coaxial cable with a conducting core and sheath using sheets of semi-industrial, aligned CNT fibres (Fig. 18 a,b). Cables of KAuBr_4_-doped CNT fibres showed performance approaching operational requirements in attenuation/length at about a fifth of the weight [142]. This weight reduction may be a low estimate, considering the significant improvements in electrical conductivity on CNT fibres since such cables were made in 2012. Indeed, CNT fibre coaxial cables for data transmission are already marketed by industrial producers and are claimed to offer 70% weight reductions relative to metallic alternatives.

In other developments, the European Graphene Flagship project developed a 2.5 m-long Cu cable coated with graphene. Coating with graphene increased electrical conductivity by 1%. A continuous coating process developed by Aixtron, a producer of nanomaterials synthesis equipment, is expected to enable the production of 1500 m/min of such cables.

Another area particularly suited to nanomaterials is printed electronics and the integration of electrical functions in structures or parts. The main motivation for printed circuits and integrated electronics are cabin electrics and data transmission. Potential benefits include a reduction in the number of parts, increased automation and highly simplified wiring and harness installation.

Conductor durability and reliability under harsh mechanical manipulation are important considerations for integrated conductors. For a glimpse of the burden of cable inspection and maintenance in transport-related applications, data from the US Navy indicates that they use 1–4 million man hours, and $94 million are spent annually in time and materials to address wiring faults [143]. Other critical aspects of integrated conductors are adherence to substrate, durability, maintenance and repairability, and compatibility between wiring and part/substrate certification. Table 6 includes the dominant properties for cabin printed circuits or electrics.

**Table 6:** Integrated conductors for cabin

Integrated conductors	Type	Relevant specification
Inks printed on thermoplastic layer/foil	Conductive ink (cured)	Viscosity (uncured ink) 30–55 Pa s Electrical conductivity 3 × 10^6^ S/m Tensile ductility > 0.5%
Cabin electrics / data transfer	Conductive adhesive (cures)	CTE < 70 × 10^–6^/K Electrical conductivity 10^6^ S/m Tensile yield strain < 20% Tensile strain at break < 50%

**Fig. 19:**
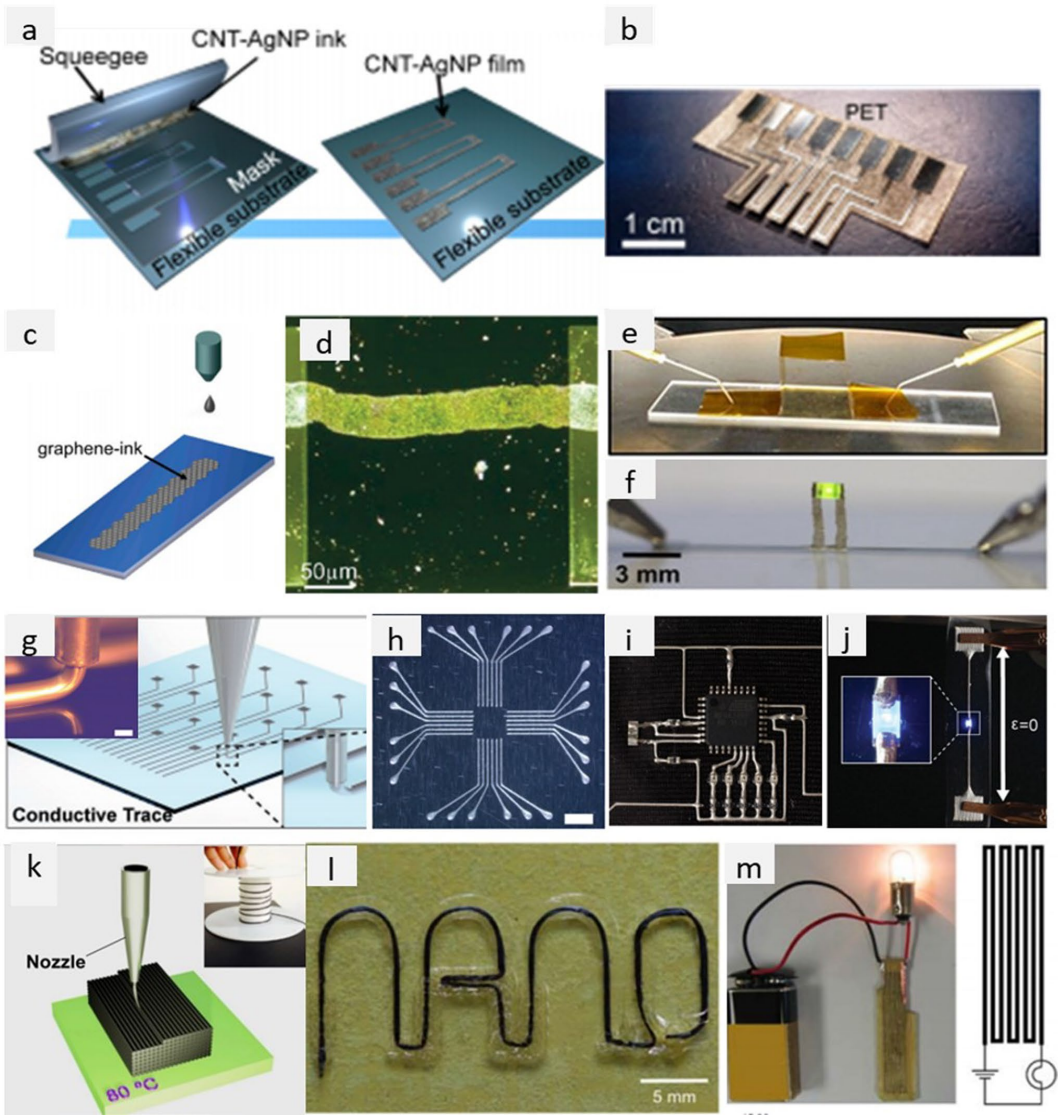
**a**, **b** Schematic of the screen-printing method and the device printed on PET substrate [146]; **c** schematic of the inkjet printing the graphene-based ink on Si/SiO_2_ substrate with **d** the optical micrograph of inkjet-printed graphene stripe on HMDS-treated substrate with minimised “coffee-ring” effect [150]; examples of inkjet printed **e** highly-flexible graphene lines printed on Kapton film [154] and **f** pillars fabricated from silver nanoparticles assembled with a light emitting diode [155]; **g** schematic of the direct ink writing method (inset: direct writing of Ag/TPU ink through the 200 µm-nozzle, scale bar 200 µm) with examples of printed **h** electrodes in a 24-pad wiring scheme with electrode widths 100 µm, **i** microcontroller circuit fabricated by hybrid 3D printing, and **j** LED device showing interface of surface mount LED connected with Ag/TPU electrodes [156]; **k** schematic of the fused deposition modelling 3D printing process [157] with examples of **l** the letters “NANO” printed using a single CNT yarn-based filament showing 90°, 180° and large radius turns, and **m** powering the 1W bulb through the printed resin-rich specimen with continuous CNT yarn [158]. **a**, **b** Adapted from [146] with permission. Copyright 2014 American Chemical Society. **c**, **d** Adapted from [150] with permission. Copyright 2012 American Chemical Society. **e** Adapted from [154] with permission. Copyright 2012 American Chemical Society. **f** Adapted from [155] under the Creative Commons CC-BY license. **g**–**j** Adapted from [156] with permissions. Copyright 2017 John Wiley and Sons. **k** Adapted from [157] under the Creative Commons CC-BY 4.0 license. **l**, **m** Adapted from [158] with permissions. Copyright 2016 Elsevier

Printing electric circuits involves, in general, the deposition of conducting particles on a substrate through various methods (Table 7) to create an electrically conducting path, which is physically attached to the substrate. Screen printing, inkjet, and direct ink writing have been applied to print electric circuits with various conductive nanofillers (Fig. 19) and could be considered the most promising methods for electrical integration in aircraft cabin parts.

**Table 7:** Comparison of different printing methods (adapted from [144])

Printing method	Ink viscosity, Pa⋅s	Line thickness, µm
Screen	0.5–10	5–100
Inkjet	0.001–0.02	< 1
Direct ink writing	20 to 10^3^–10^5^ [145]	Various

In screen printing, the printed pattern is defined by the size of the gaps in the printing template (screen mask resolution) and is compatible with various inks and substrates. For example, Harada et al*.* [146] have printed the conductive CNT/AgNP composite electrodes with a high loading of Ag nanoparticles (up to 50%wt). Hyun et al*.* demonstrated screen-printed graphene lines on polyimide films with high electrical conductivity of ~ 1.86 × 10^4^ S/m and flexibility from inks of different viscosities (1 and 10 Pa⋅s) [147].

Inkjet printing is a scalable technology based on the same principle of depositing liquid droplets on a substrate as in paper printers [148]. In the printed liquid droplet, the self-assembly of nanoparticles during drying forms a thin and planar conductive layer on the substrate. The generation of liquid droplets and the homogeneity of inkjet-printed drops are affected by many parameters, including surface tension, density, nozzle diameter, and ink viscosity. The “coffee ring effect”, as a consequence of extra low viscosity and distortion of the drops during solvent drying, is a common problem of inkjet printing and requires specific ink formulation to control evaporation of the ink and/or surface modification of the substrate [149, 150]. The formulations are usually complex, including thickening agents to adjust the viscosity to the desired value (e.g., polyvinyl alcohol [151]), surfactants to modify surface tension, dispersants to stabilise conductive colloids, humectants (alcohols, glycols) to mitigate fast drying at the nozzle and control evaporation of the ink, and others, such as adhesion promoters, pH buffers, de-foamers, etc*.* [152, 153]. The corresponding inks should comply with very low viscosity requirements for thermal (1–5 cP or 0.001–0.005 Pa⋅s) and piezoelectric printheads (5–20 cP or 0.005–0.02 Pa⋅s) [151]. These viscosities are extremely low compared to the expected viscosity (30–55 Pa⋅s) for inks with sufficient volume fraction of 1D nanomaterials to achieve the electrical specifications for integrated conductors for the cabin (Table 6).

Direct ink writing can be used to print electric circuits from the conductive inks of different viscosity, ranging from low-viscous liquid to paste [145, 148], resulting in printed conductive paths of different thicknesses. The composite conductive inks exhibit strong shear thinning behaviour so that their viscosity reduces from ~ 10^3^–10^5^ Pa⋅s [145] to < 50 Pa⋅s [156, 159] at higher shear rates, enabling their printing from the fine nozzles. Usually, such composite conductive inks for direct ink writing are characterised by the high storage modulus and high shear yield stress [160] that help to retain the filamentary shape and integrity after passing through the nozzle.

The printed conductive circuit is expected to replace the bulky and heavy wires currently made of copper without compromising their electrical performance. The electrical conductivity of inks in both inject printing and direct ink writing depends on the volume fraction of conductive filler and follows the theory of percolating networks. For inject printing, conductive inks with various properties could be made with metals (for example, a suspension of nanoparticles, such as silver or copper), conductive polymers such as PPV (polyphenylene vinylene) and PEDOT:PSS (poly (3,4-ethylenedioxythiophene):poly (styrene sulfonic acid)), carbon nanomaterials, or their hybrids (Table 8). Metal-nanoparticle-based inks [151, 152] offer the highest electrical conductivity but can be rather expensive, and their sintering could impose practical limitations on the choice of printing substrate.

**Table 8:** Comparison of electrical properties of different inks for inkjet printed electronics (adapted from [144])

Ink type	Conductive material	Conductivity (S/m)
Metal nano particles	Ag	~ 10^6^–10^7^
Organic polymer	PEDOT: PSS	< 10^6^
	PPV	< 10^4^
Nano carbon	CNT	~ 10^4^ [161]
	Graphene	~ 10^2^ [150]—10^4^ [154]

Carbon nanomaterial-based inks with carbon nanotubes and graphene benefit from lightweight and printability on a wide range of substrates; however, they show modest electrical conductivity [150]. If the high loadings of conductive fillers are necessary to reach the desirable level of conductivity, the rising viscosity will impose technical limitations. In direct ink writing, due to the shear forces occurring when the viscous fluid passes through the nozzle, the highly anisotropic conductive particles undergo shear-induced alignment during printing. This increases the electrical conductivity to above 10^6^ S/m for Ag/TPU printed circuits [156], to 4.5 × 10^4^ S/m for RGO-based [159], and up to 10^4^ S/m for CNT-based printed patterns [161], which are among the highest values reported for printed graphene and CNT-based electronics.

Apart from printing itself, component integration is another critical aspect. In any case, printed electrodes should retain good bonding to the underlying substrates; a robust interface is required to prevent delamination [162, 163].

Another direction to integrate the conductive paths of tailored rigidity (or flexibility) in complex 3D structures, eliminating the associated problem of the percolation threshold for conductive inks with discrete fillers, is using the aligned nanostructured ensembles of nanoparticles as continuous fibres or yarns. In principle, they can act as a continuous conductive core for the composite filament in fused filament fabrication (fused deposition modelling)–3D printing technique of the complex and thick structures with a solid thermoplastic filament. This emerging area will require developing composite filaments with continuous nanostructured fibres/yarns with control over compatibility with polymer matrix, wettability, and the degree of polymer infiltration [164]. Early pioneer works have demonstrated the printability of highly-densified CNT yarns/PEI with high loading of CNTs (> 40% vol.) [158] and CNT yarn-core with thick Nylon coating [165]. Although no detailed analysis of the electrical conductivity of printed parts has been performed, the study demonstrated that a resin-rich 3D printed specimen with an embedded CNT yarn was able to power a 1W bulb and showed the 180° turns possible with continuous CNT yarn (Fig. 19 l,m).

### Anti-icing and de-icing

**Fig. 20:**
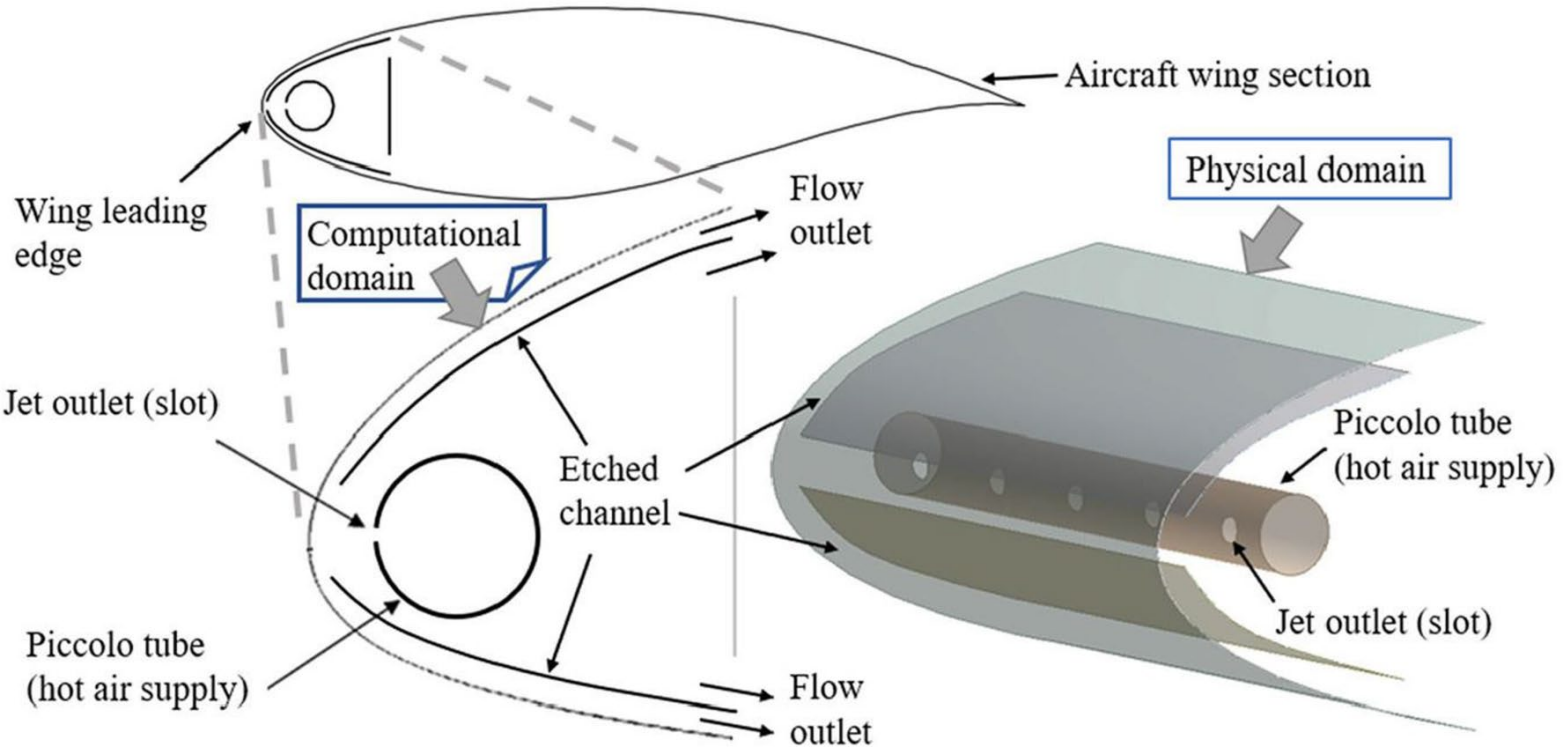
Example of  the ice protection system (IPS) —Piccolo tube [167]. Reprinted from [167] under the Creative Commons CC-BY 4.0 license

Commercial aircraft need the implementation of anti-icing (ice prevention) and de-icing (ice elimination or mitigation) systems in order to avoid detrimental effects on aerodynamic profiles and any linked safety concerns [166]. Current solutions are mainly based on airflow, that is, Piccolo tubes, which consist of titanium pipes inserted into the wing slat. They distribute the hot air from the engine to the leading edge to avoid ice accumulation on the wing surface, see Fig. 20.

However, these “bleed” systems often cannot be made of lightweight composite parts due to the high operation temperature required and the low heat transfer efficiency of some CFRPs. Moreover, this heating method is not suitable for electric/hybrid aircraft, which will therefore require electro-thermal ice protection systems (IPS). In current aircraft, the key areas requiring IPS include wing leading edge, engine inlets, nacelle lips and pylon structures, although it would also be beneficial to use them on the Horizontal or Vertical Tail Plane (HTP or VTP). A common de-icing system utilises pneumatically inflated rubber boots usually attached to the leading edges of the wings and stabilisers, especially in twin-engine aircraft models. These boots can be expanded under the formed ice to break it, although a particular thickness of ice needs to be developed before the application, and any disturbance to airflow caused by inflated tubes should be kept to a minimum [168].

Electro-thermal anti-icing systems use conductive circuits to generate heat through Joule heating. Proposed electro-thermal ice-protection systems are multi-layer solutions consisting of a top layer, a heater-electrical circuit including connectors to apply the electrical current, and an insulating layer. Some drivers for selecting the different elements of this multilayer system are included in Table 9.

**Table 9:** Dominant drivers for electro-thermal Ice Protection System (IPS) approach

Layer	Drivers/properties
Top layer	Protection of heater against damage
	High temperature resistance
	Easy to integrate in CFRP
	Qualified for airframe
Heater/electrical circuit	Electrical properties for specific high heat output/anti-icing effect:
	-Specific heat power or heat flux: 20–50 kW/m^2^
	-Total power (on aircraft level): ~ 150–160 kW (Anti-icing, fully evaporative)
	Surface temperature, in icing conditions:
	-Anti-icing: for fully evaporative anti-icing 40 °C
	-De-icing: for a non-evaporative system, surface temperature > 0 °C
	Voltage: 230 V AC, 3-phase, or 540 V DC (± 270 V)—More electrical AC
	Homogeneous heating, no high temperature difference in heated area
	Low density
	Easy to manufacture & integrate in CFRP
	Chemically and thermally stable
	Flexible
Connectors	Mechanically stable
	Good behavior against corrosion (chemical stable)
Insulating layer	Thermal and electrical insulation against CFRP material
	High temperature resistance
	Easy to integrate in CFRP

A structural composite with a graphene anti-icing sheet was developed by the Graphene Flagship [166]. The anti-icing layer was produced from graphene nanoplatelets (GNP) preassembled in sheets. The sheets have high electrical and thermal conductivity, good bendability and processability, and are chemically and thermally stable [169, 170] under the envisaged operation conditions. A multi-layer system was used to integrate graphene paper within CFRP [171, 172].

**Fig. 21:**
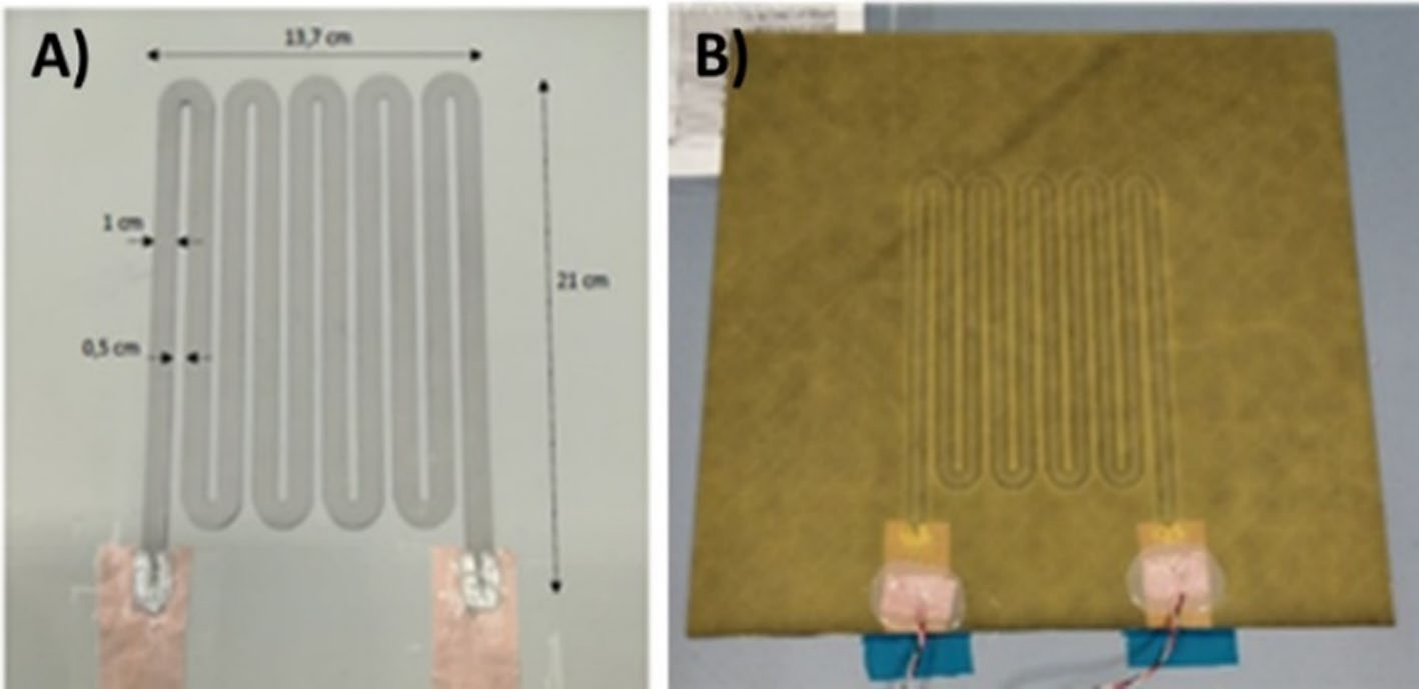
**a** Graphene serpentine/circuit for ice protection system, **b** flat panel with integrated graphene serpentine by co-curing. **a**, **b** Reproduced from [173] under the Creative Commons BY-NC-SA license

The serpentines are graphene sheets with a sheet resistance (Rs) of about 0.1 Ohm/sq, patterned using an automatic cutting machine to obtain the final shape [166, 173]. The selected graphene design for this project is serpentine to optimise power consumption (Fig. 21). The typical circuit resistance is 20–30 Ohm. Ending connections of this circuit are bonded by silver paint and conductive epoxy to ribbons of copper grid, to which cables will be attached to introduce the electrical current.

**Fig. 22:**
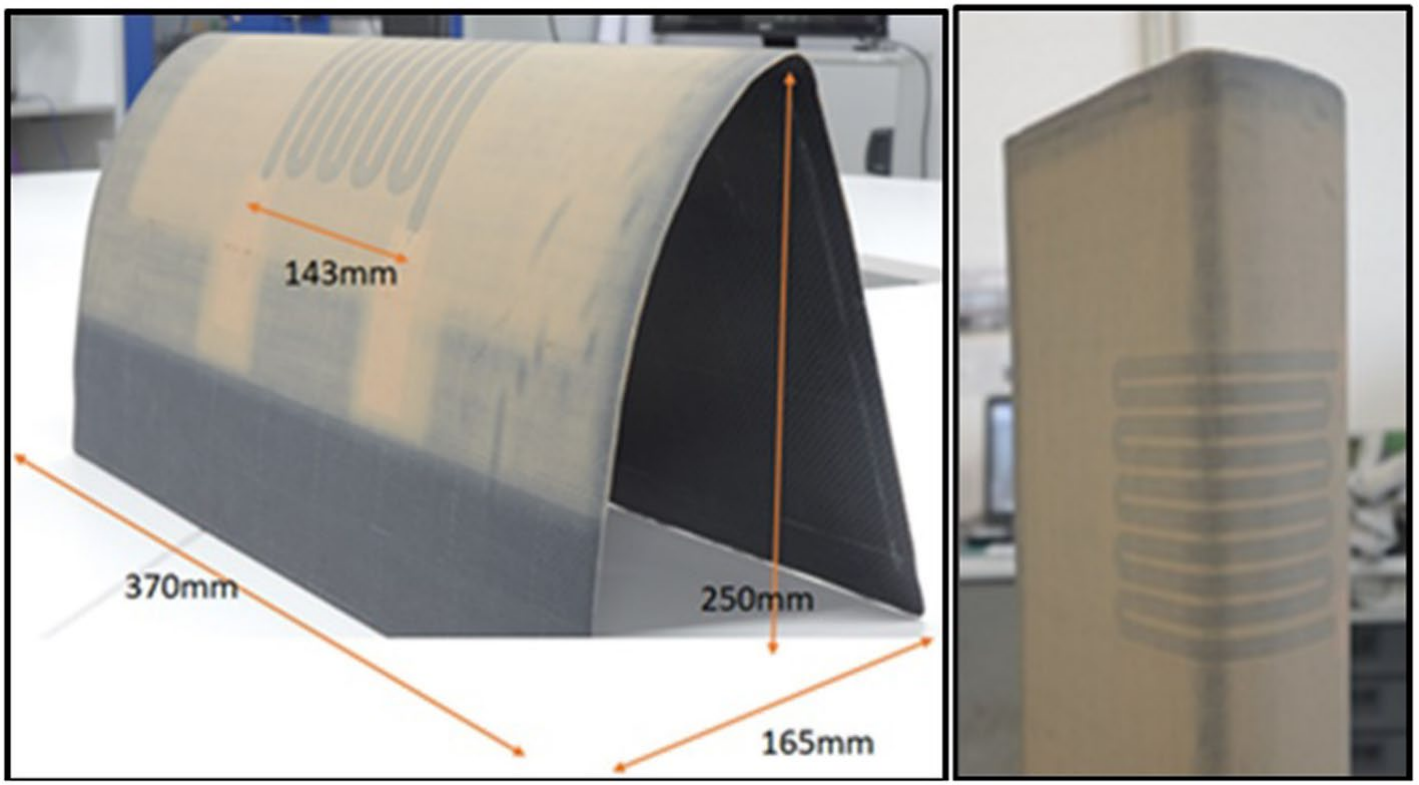
Curved panel (HTP leading edge shape) with integrated graphene serpentine by co-curing. Reproduced from [173] under the Creative Commons BY-NC-SA license

The de-icing concept was demonstrated on complex-shaped panels. The graphene serpentine was co-cured in an oven with CFRP and insulation layers on a curved mould, which is representative of a Single Aisle HTP leading edge shape, obtaining good integration quality results without visual defects, as it can be seen in Fig. 22.

Joule heating elements made with laser-induced graphene layers demonstrated rapid de-icing when incorporated as an interlayer in GFRP [174]. Heating elements made of highly aligned CNT webs produced by chemical vapour deposition (CVD) introduced in GFRP demonstrated electro-thermal behaviour suitable for rapid de-icing [175, 176]. In the CFRP prototype containing 40 layers of CNT, web ice was removed within 15 s at a constant power supply of 4.9 kW/m^2^.

## Energy storage and conversion

### Electrochemical energy storage methods for aviation

**Fig. 23:**
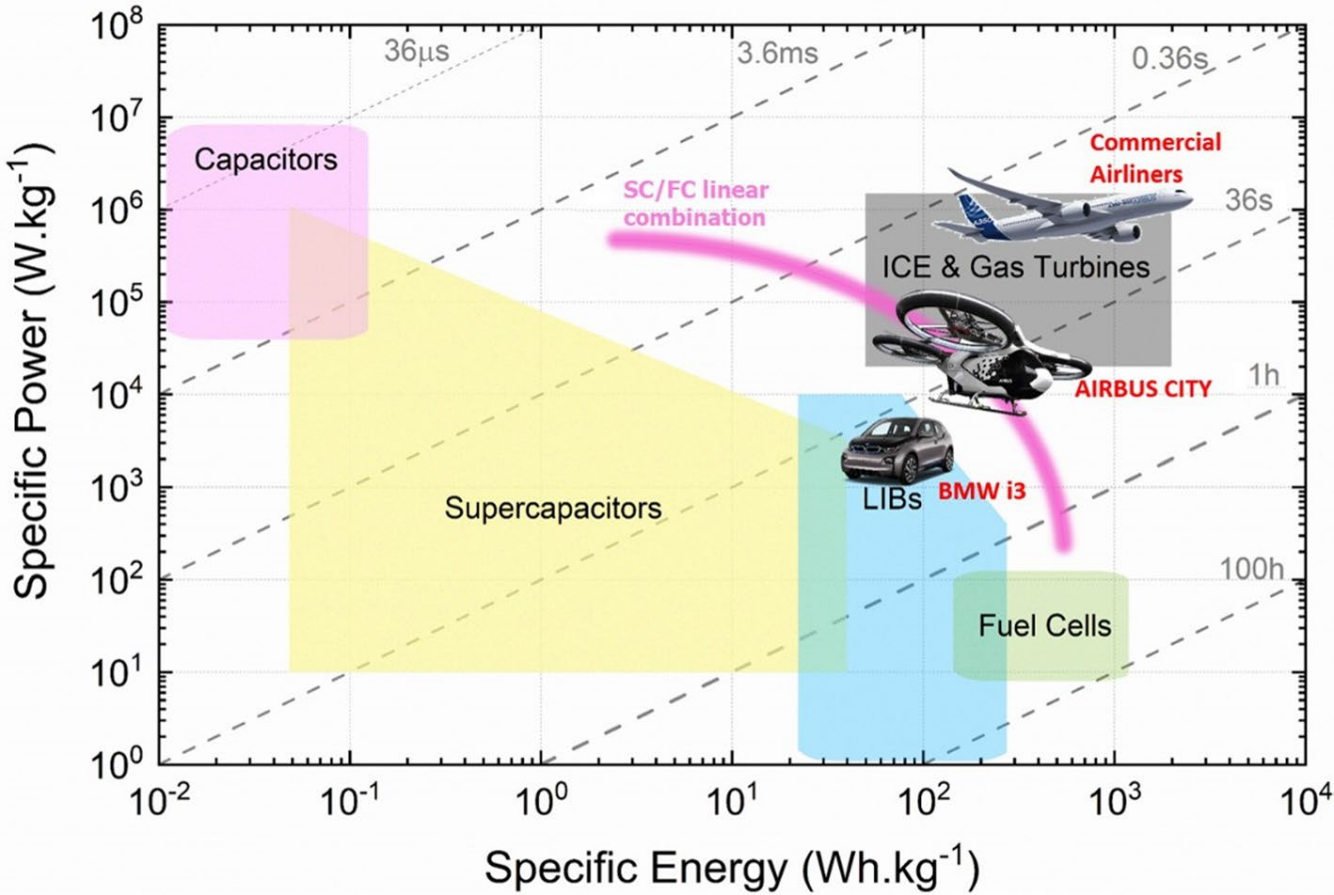
Ragone plot for different energy storage systems, including ICE and a rule of mixture for a linear combination of fuel cells and supercapacitors. Some example electric/hybrid concept vehicles are superimposed. Copyright 2024 IMDEA Materials

Vehicles in civil aviation range from small unmanned aerial vehicles (UAV) of less than a kilogram with flight times of a few hours to large aircraft of hundreds of tonnes for long-range flight. The corresponding requirements of propulsion power and energy density are equally broad and span across different electrochemical energy storage technologies, even for hybrid aircraft combining electrical propulsion with gas turbines. As a global image, Fig. 23 shows a Ragone plot (e.g., specific power vs. specific energy) for different powering devices and superimposed reference electric/hybrid concept aircraft. Most aircraft electrification concepts for propulsion anticipate combinations of different electrochemical systems for combined high thrust and long range. Thus, the graph includes for reference a curve corresponding to the linear combination of supercapacitors and fuel cells. The comparison highlights the challenge of replacing jet fuel, which is about 30 times more energy-dense than state-of-the-art commercial lithium-ion cells, for example. Indeed, pure electric propulsion is not yet a feasible replacement for gas turbine motors [177]. Detailed reviews on different electrical architectures for increasing electric aircraft ranges and the relative importance of different energy storage contributions can be found elsewhere [178, 179]. Here, we focus on contributions to improving the storage performance of batteries, supercapacitors, and fuel cells by using nanomaterials and their possible impact on their development/integration towards more/all-electric aircraft.

### Nanomaterials in batteries

**Fig. 24:**
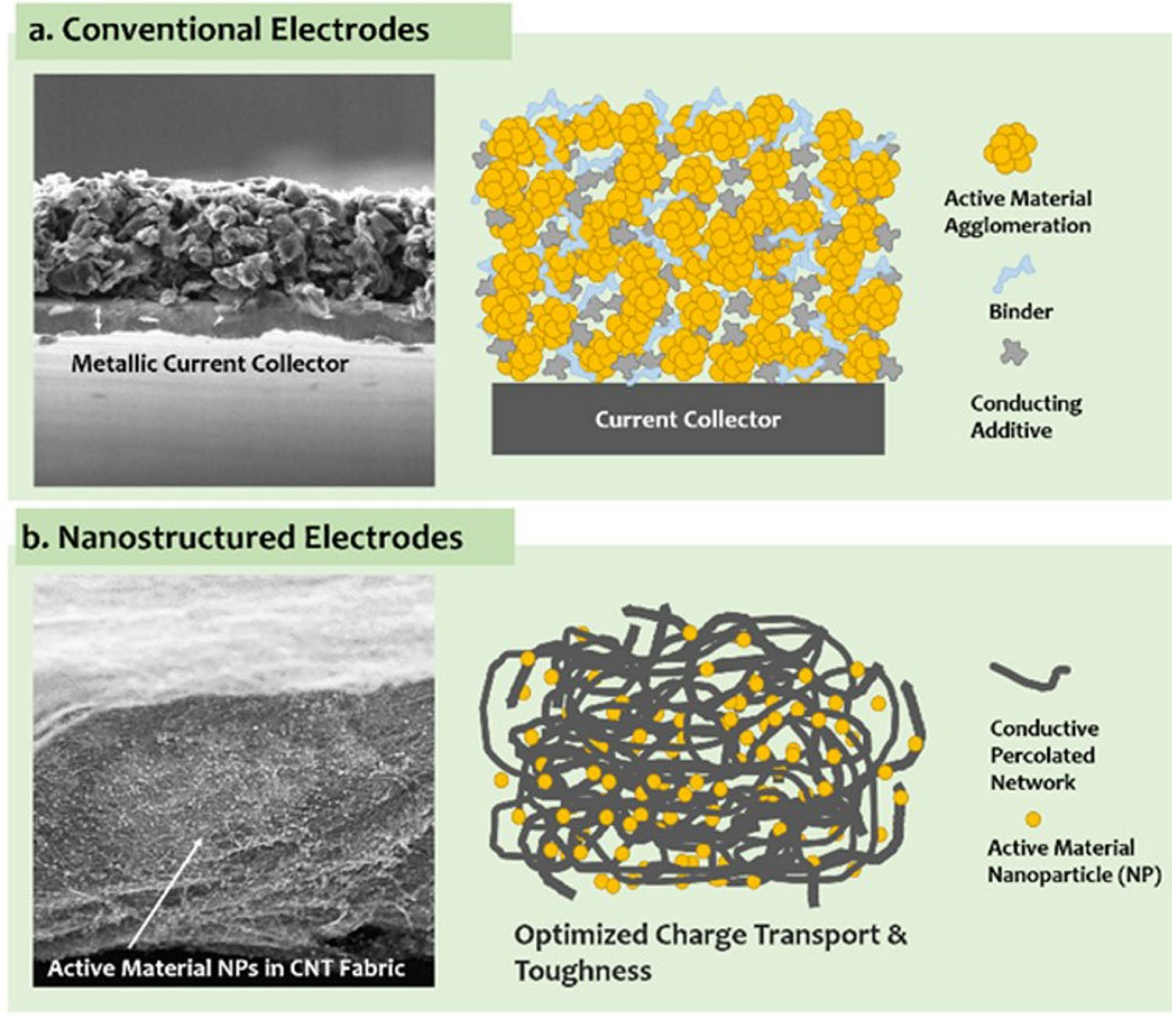
Comparison of the microstructure of **a** a traditional battery electrode and **b** a nanostructured electrode with carbon network. Copyright 2024 IMDEA Materials

Batteries are possibly the most effective application of nanomaterials [180] since they can improve capacity [181], power [182], cyclability [183], provide alternative electrode possibilities [184] and manufacture in ways inaccessible to conventional materials. The conventional configuration of traditional electrodes consists of aggregated microparticles of active material and conductive additive particulates glued together by a polymeric binder and supported on a metallic foil as the current collector. An example of this “granular” electrode and its schematic representation is shown in Fig. 24a. Alongside, we show an example of a nanostructured electrode consisting of a percolated network of high-aspect-ratio nanocarbons, acting as a scaffold or built-in current collector, hosting nanosized active material. This nanostructured electrode (Fig. 24b) resembles a composite and captures the three key features that result in an overall improvement in electrode performance in battery: a higher electrical conductivity, both in and out of the plane; a higher toughness; and a smaller size of active material.

#### Nanocarbon network for increased conductivity, toughness, and buffer effects in composite electrodes

**Fig. 25:**
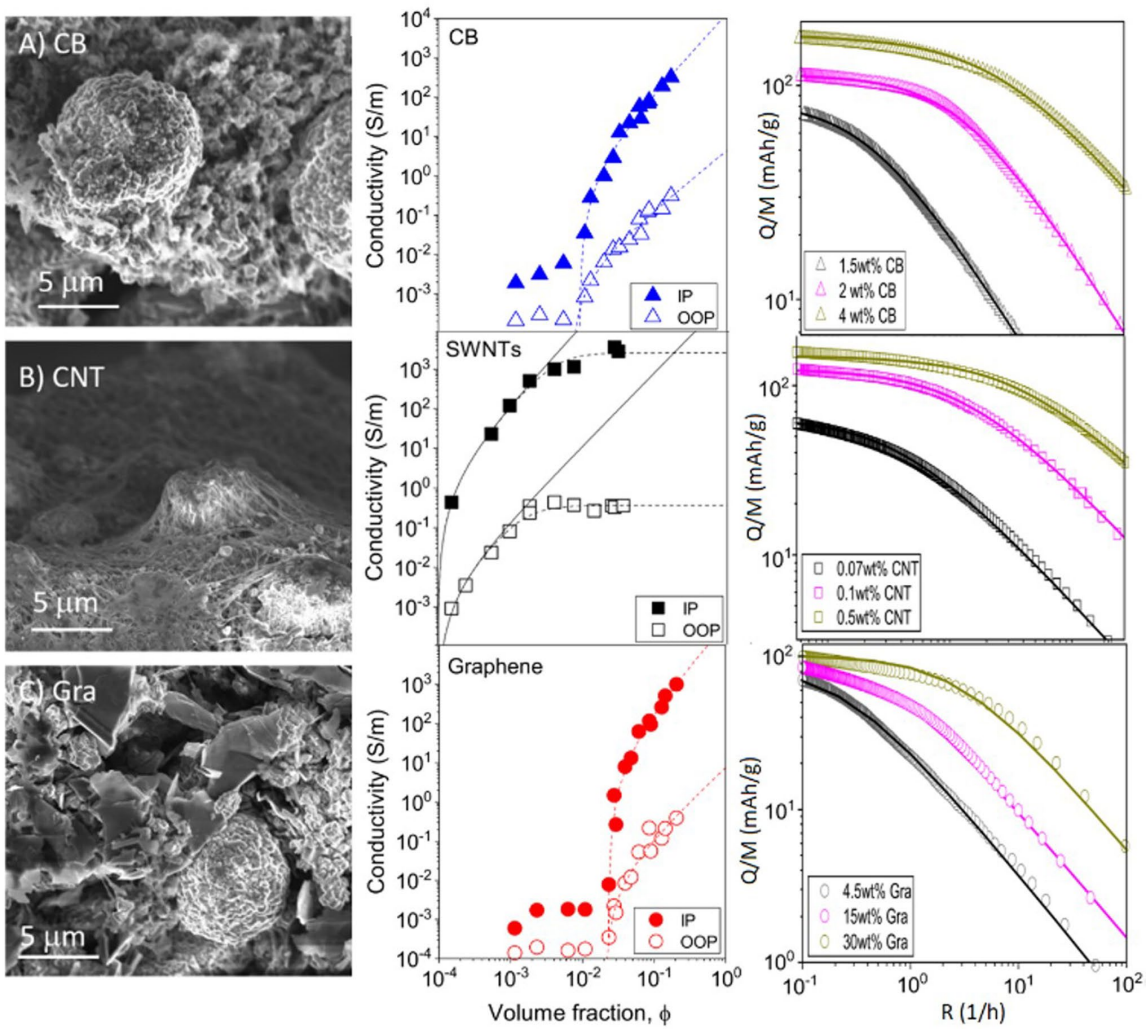
SEM images of the LIB cathodes (e.g., NMC 811) with different conducting fillers: **a** carbon black, **b** SWCNT, and **c** graphene layers with various mass fraction of the filler. For each case, the in-plane (IP) and out-of-plane (OOP) conductivities and their specific capacities (Q/M) vs. charge–discharge rate (R = (I/M)/(Q/M)) in several mass fractions are depicted. Due to high aspect ratio, SWCNTs form percolating networks at lower mass fractions. The threshold for electrical limitations on capacity at high rates is an OOP conductivity of ~ 1 S/m [186]. **a**–**c** Adapted from [186] with permission. Copyright 2020 American Chemical Society

Replacing traditional conductive fillers used in LIBs, such as quasi-spherical carbon black (CB) particles, with high-aspect-ratio CNTs or graphene has been extensively explored over the last decades. Through the formation of conducting percolating networks, nanocarbons give access to electrical conductivity not accessible in traditional electrodes and can thus result in capacity approaching near-theoretical values for a variety of chemistries [185]. A recent study comparing the rate capability of NMC cathodes with different conductive fillers provides compelling evidence of the advantage of high-aspect-ratio nanocarbons. Using a mechanistic analysis, the authors separate the factors limiting the capacity of LIB electrodes [186] and compare electrical transport limitations in cathodes with different contents of highly conducting SWCNTs, graphene, and CB. Their results show that optimum electrode performance requires (amongst other factors) decreasing RC charging time, translating into a minimum out-of-plane electrical conductivity of ∼1 S/m. For traditional anodes the high electrical conductivity of graphite (10^4^ S/m) is above this threshold, but most other anode (e.g., Li_4_Ti_5_O_12_, σ ranging from 10^–7^ to 10^–11^ S/m) [187] and cathode (e.g., LiFePO_4_, σ ~ 10^–8^ S/m) [188] materials are essentially electrical insulators. In their study with NMC, these authors found that SWCNTs provided much higher values of out-of-plane electrical conductivity than CB or graphene at all mass fractions produced (Fig. 25), mainly a consequence of the higher aspect ratio of these one-dimensional conductors leading to more conducting networks at lower concentrations. The electron micrographs in Fig. 25 qualitatively show this higher degree of interconnection for CNTs, even below 1% wt., visible as a continuous “mesh” wrapping the active material particles.

The example in Fig. 25 is particularly illustrative in capturing the capacity improvement associated with different nanofillers and identifying the corresponding electrical properties. Multiple other processing methods exploit different routes to form graphene networks [189] or CNTs to produce electrically conducting LIB electrodes. A promising method is to introduce active materials to pre-formed sheets of CNTs, for example, by spraying dispersion of the active material [190] or direct growth of the active material on the internal pores of the porous conductor [191]. The resulting composite structures are characterised by a low internal electrical resistance. Interestingly, despite the predominant in-plane alignment of the CNT fibre sheets, out-of-plane electrical conductivities of the order of 1 s/m are found in composite electrodes with 16–31% mass fraction of CNTs [110].

In addition, nanocomposite electrodes with an internal network of high-aspect-ratio nanoparticles show higher stability under repeated charge/discharge cycles. As an example, electrodes of few-layer MoS_2_ have shown high capacity retention of repeated cycles at high current density when comprising a network of CNTs (∼950 mAh/g after 500 cycles at 2 A/g), compared to < 10 cycles for an electrode with conventional CB [50]. Several other active materials have shown increases in cyclability when combined with a nanostructure-supporting network, including emerging high-energy LIB anodes (Si) [185] and cathodes (FeF_2_) [192]. The conducting network immobilises active materials during repeated cycling and thus has a mechanical role. Indeed, in a study of CNT/MoS_2_ nanocomposite electrodes, a correlation between tensile fracture energy and cyclability was observed [50]. Overall, the formation of a composite electrode structure with higher toughness is seen as beneficial to contain the volumetric expansion of the active material and prevent electrochemical milling; however, in most systems, the electrical and mechanical contributions of the nanocarbon network are difficult to decouple.

In the case of S cathodes, there is additionally extensive evidence that the integration of nanocarbon network not only provides the cathode with electronic conductivity (sulfur is practically insulating, ~ 10–30 S/cm) [193] but also mitigates polysulfide shuttling, a known problem in Li–S batteries. In this fashion, the porous carbon acts as a storage container for the active material in its micropores, improving the cyclability of the Li–S batteries [194]. Similarly, a high specific capacity of 1320 mAh/g, approaching 80% sulfur utilisation, and stable cyclability were accomplished by impregnating sulfur into a highly ordered nanosized mesoporous carbon [195]. It has been demonstrated that the morphology of the rearranged sulfur is influenced by the surrounding conductive carbon host rather than the S original morphology. As a result, various carbon networks in the S − porous C composites have been employed to evaluate and improve the reversibility and cyclability of the Li–S system. Although the microporous carbons (*i.e.,* pore size < 2 nm) have been found to be the ideal container for accommodating and immobilising the active material, their small pore size limits the S content and also impedes the Li-ion and electrolyte transfer. Therefore, porosity must be controlled to accommodate a high content of the active material and to avoid polysulfide shuttling. The macro- to meso-porosity in interwoven carbon nanofibres or carbon nanotube networks can lead to excellent electrolyte immersion and suppress polysulfide migration due to their high electrolyte uptake [196, 197].

**Fig. 26:**
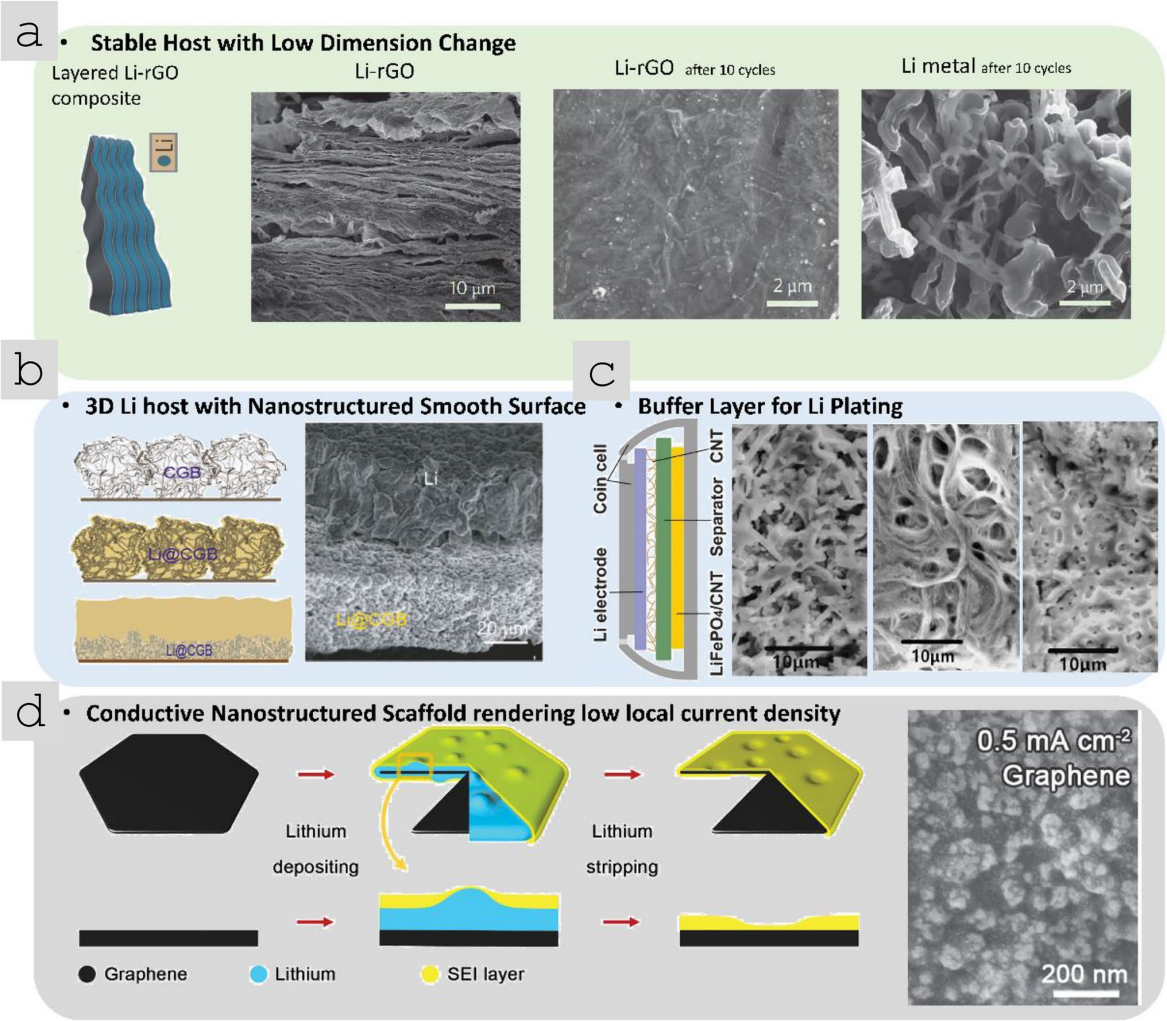
The application of lithiophilic nanocarbons as host or buffer to retain Li for batteries with high energy density. **a** layered rGO with nanoscale interlayer gaps as stable Li host with low dimension variation of ~ 20%, retaining a high capacity of ~ 3390 mAh/g and flat voltage. SEM micrographs of the sample after 10 cycles are compared to that of metallic Li electrode [200]. **b** Schematic showing crumpled graphene balls (CGB) capable to support high Li metal loading without volume fluctuation and further Li deposition on top of the sample without dendrite formation. SEM shows the Li deposited on a 40 µm thick CGB electrode [202]. **c** The effect of CNT as a buffer layer to store Li during plating/stripping. SEM micrographs show the comparison of the Li metal with and without the CNT buffer layer after cycling [203]. **d** Schematic diagram of the Li deposition and stripping process on one graphene flake along with SEM micrograph of the electrode after Li deposition at 0.5 mA/cm.^2^ [204]. **a** Adapted from [200] with permission. Copyright 2016 Springer Nature. **b** Adapted from [202] with permission. Copyright 2018 Elsevier. **c** Adapted from [203] with permission. Copyright 2016 Royal Society of Chemistry. **d** Adapted from [204] with permission. Copyright 2016 John Wiley and Sons

Similarly, nanocarbons have shown to prevent formation of dendrites when using Li as anode [198, 199] (see Fig. 26).

This has been observed in various nanocarbon ensembles, such as graphene-derived samples (e.g., sparked rGO, scaffolds [200], graphene-crumpled balls [202], or its composites with CNTs [201, 205], etc*.*), graphitised carbon nanofibres [206–209], and thin films of CNTs [203]. This is due to ultralow local current densities, which favour smooth plating of the Li. Effectively, the carbon network acts as a buffer layer during Li plating/stripping; its high surface area provides a reservoir for uniform/facilitated Li nucleation, thus mitigating the dendrite formation on the underlying metallic surface. Such architectures can also reduce charge transfer resistance and enhance Li + ion diffusivity. Overall, the integration of nanocarbons leads to high capacities (> 3300 mAh/g) and high cyclability at higher rates, thus improving the safety of the battery.

#### Increasing stability and diffusion in nanosized active materials

The stabilisation of active materials that undergo large deformations upon ion intercalation/alloying can be achieved by reducing one of the dimensions to the nanoscale. This is particularly relevant for Si, envisaged as a possible replacement of natural graphite in the next generation of LIBs [210]. The critical sizes to prevent fracture propagation in crystalline and amorphous Si particles are 50 and 870 nm, respectively [211]. Thus, nanostructured silicon can sustain repeated volumetric expansions of over 300% without fracturing, attaining impressive capacities of over 3000 mAh/g after 20–200 cycles [46, 212].

In addition, reducing the size of active material can also overcome limitations from slow ionic solid-state diffusion. For example, the low diffusion coefficient of Li-ion in silicon (~ 10–14 cm^2^/s in crystalline Si) [213] implies that particle sizes as small as 100 nm are required to extract high capacity at high rates, that is, for high power. Other cathodes and anodes have similarly low solid-state diffusion coefficients and thus benefit from nanostructuring and/or nanocoatings. For instance, the poor rate capability of NMC cathode materials can be mitigated via synthesis as a nanostructured material rich in the {010} facets [214]. Similarly, in Li-rich layered oxides (e.g., Li_1.2_Mn_0.54_Ni_0.13_Co_0.13_O_2_), the Li diffusion rate can be increased with nanoscale surface coatings of spinel oxides [215]. The addition of a thin nanoscopic layer of turbostratic carbon on the surface of graphite anodes has also been shown to improve charging capability. Such behaviour is attributed to the presence of active sites and additional fast Li + diffusion pathways at the basal‐plane side of graphite sheets, which increase in the Li + diffusion rate and reduce polarisation in a working battery [216]. Moreover, the fabrication of composites through the hybridisation of inorganic nanomaterials and reinforcing active components’ chemical/structural stability was demonstrated as an effective strategy to improve battery electrode performance, including cycle life [217], even in chemistries beyond Li [218].

#### Electrode fabrication and cell improvements

**Fig. 27:**
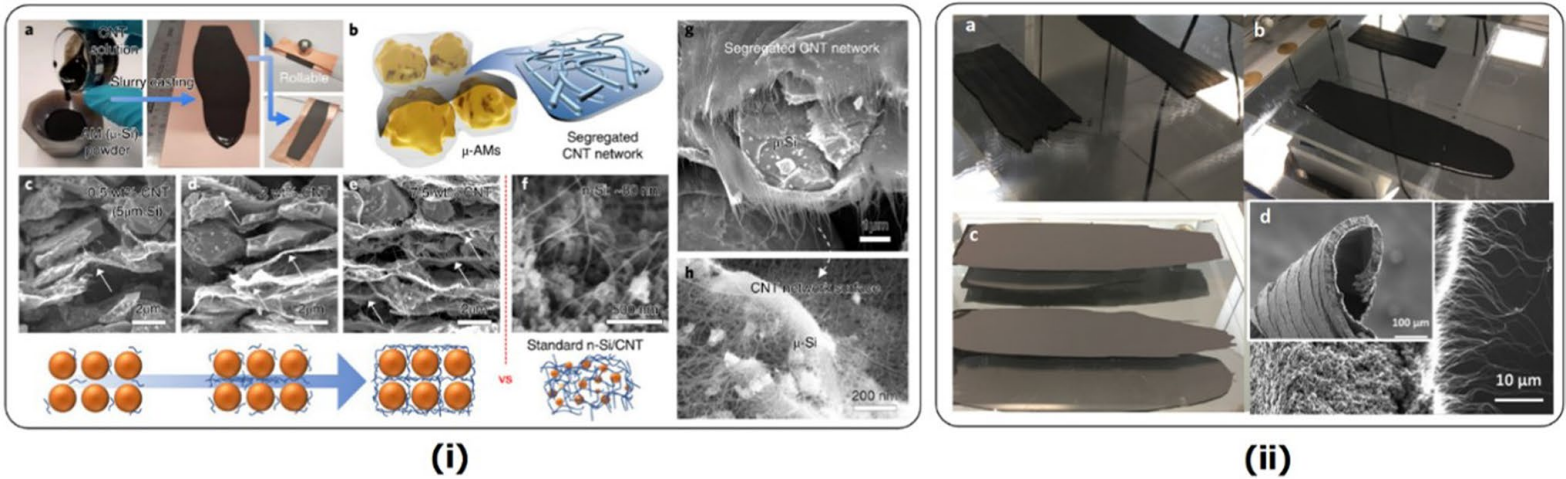
Nanocomposite electrodes with reduced content of materials. **(i)** **a**, **b** Nanocomposite of Si and 7.5% wt. (optimised) of SWCNT, without polymeric binder [185]. As it is seen from the SEM micrographs (**c**-**h**) the segregated network could be achieved at > 1% wt. of SWCNTs for microsized Si particles while such network did not form in the case of Si NPs. **(ii)** Olivine LiFePO_4_ cathode electrodes with CNT fibre-based fabrics as current collector accounting for 18% wt., compared to 58% wt. for regular Cu foil (electrode level)[223]: **a** the bare CNTf, **b** slurry-coated, **c** dried films and **d** cross-sectional SEM micrographs of the electrodes. **(i)** Reproduced from [185] with permission. Copyright 2019 Springer Nature. **(ii)** Adapted from [223] with permission. Copyright 2019 American Chemical Society

Nanomaterials also offer opportunities for new electrode/cell architectures that are different from traditional battery designs., An area of intensive activity is to reduce or eliminate elements that do not take a direct part in the charge storage or transfer process, thus, to reduce the weight of batteries. These components can represent 50% of the battery weight [219] (and a significant fraction of the pack price [220]), depending on battery chemistry. In the nanocomposite electrode structures described above, where the active material is held by a network of nanocarbons (Fig. 27i), the content of the conductive agent may be reduced from > 10% wt. to ~ 1% wt. [185], and the need for polymeric binder (6–10%wt.) was eliminated altogether. Similarly, depending on cell design, fabrics of CNT fibres can replace metallic foils (Fig. 27ii) and reduce the contribution of current collectors from ~ 20–25% wt. [221, 222] (single cell level) to ≈5% wt.[190, 223]. Beyond weight reductions, using nanocarbon conductors in nanocomposite electrodes may also lead to more sustainable manufacturing routes by reducing solvent use, reducing integration steps, and removing energy-intensive components. According to some estimates, using solvents to disperse and coat microparticles translates into a large footprint of approximately 400 CO_2_ per kWh [224].

#### Specificities of batteries in aviation

**Fig. 28:**
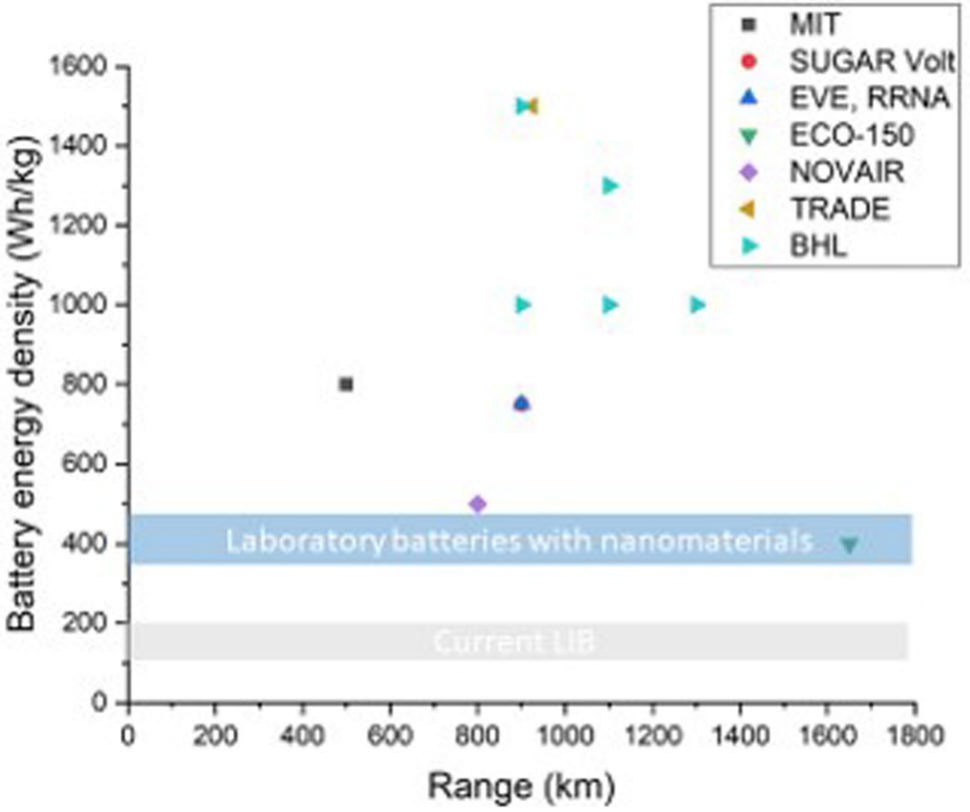
Estimated energy density required for different 180 pax hybrid/electrical concept aircraft, with current values for commercial and laboratory-scale LIBs. Copyright 2024 IMDEA Materials

In a commercial aircraft, there are several non-propulsive functions that are/can be covered by batteries. They provide auxiliary power, for example, as a buffer to prevent power peaks to the engine during take-off, to power the electric network during the descent phase or for electronic systems during emergencies. However, propulsive requirements can be altogether on a different scale. For example, we may consider a single-aisle aircraft since this type can represent 85% of present fuel consumption in aviation. Figure 28 shows the estimated energy densities required for different hybrid/electric concept aircraft for 180 pax to travel a range of 500–1700 km. To put these numbers in context, Fig. 28 shows a first range corresponding to current LIBs, for example, the type used in the automotive sector. The comparison in Fig. 28 also shows another band with recent data for laboratory-scale battery cells containing nanomaterials, which lies significantly above present LIBs. This simple comparison gives a feel for the enormous challenge of using batteries as the main propulsion in medium-large aircraft.

Nevertheless, batteries remain a key element in energy management across the complete aeronautical spectrum, from the propulsion of UAVs to distributed power in electrical systems. Table 10 lists fundamental target properties for 2030 for batteries used in airborne transport according to the Strategic Research Agenda for Batteries of the European Commission [210]. Note that these properties are not meant to be fulfilled simultaneously by single battery chemistry or construction; instead, they address the different needs for auxiliary power supply, long range and high power. The common feature of these requirements is the need for very high specific power. The properties under these demanding testing conditions for current commercial batteries are included for reference.

**Table 10:** Key battery target properties for electric or hybrid electric aircraft

Property	2017	2030
High energy battery—energy density at 8C discharge (Wh/kg)	< < 70	450
High power battery—energy density at 2C discharge (Wh/kg)	< < 70	550
Number of cycles at 3C charge, 1C discharge	< 1000^a^	> 3000

^a^Based on 90% DOD at 0.2C

**Fig. 29:**
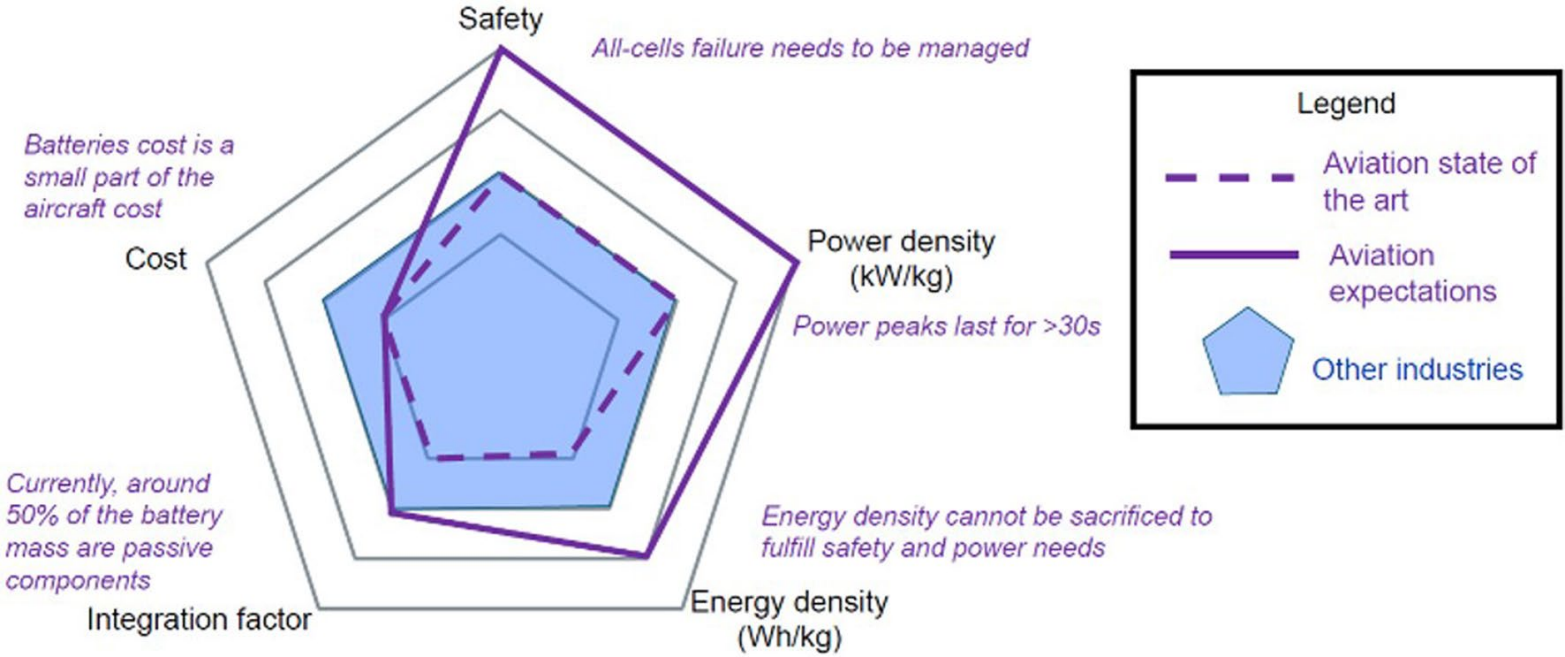
Schematic representation of the importance of different performance indicators in aviation batteries [225]. Copyright 2020 Airbus

Table 10 implicitly includes another key property of aeronautical batteries, namely safety. At present, more than 80% of batteries in conventional aircraft are Ni–Cd [225], a low energy density chemistry but considerably safer than LIBs. A schematic representation of the importance of safety relative to other performance indicators in aviation batteries is shown in Fig. 29.

Another very active avenue to reduce weight of batteries in transport is through redesigning battery packs and materials in battery cells to reduce heavy components that do not take direct part in the charge storage or transfer process. One favoured metric in the aeronautical industry is the Integration Factor, defined simply as:

3 $$IF = \frac{{Total\;cell\;weight}}{{Battery\;pack\;weight}}$$

Integration factors in conventional aircraft batteries (e.g., B787 and A350) range between 0.35 and 0.8; however, these are for the low energy density chemistries currently used in aviation. In the automotive industry, IFs above 0.8 for LIBs are common. From a device perspective, the target would be to achieve performance in line with Table 10, together with IFs above 0.75.

**Fig. 30:**
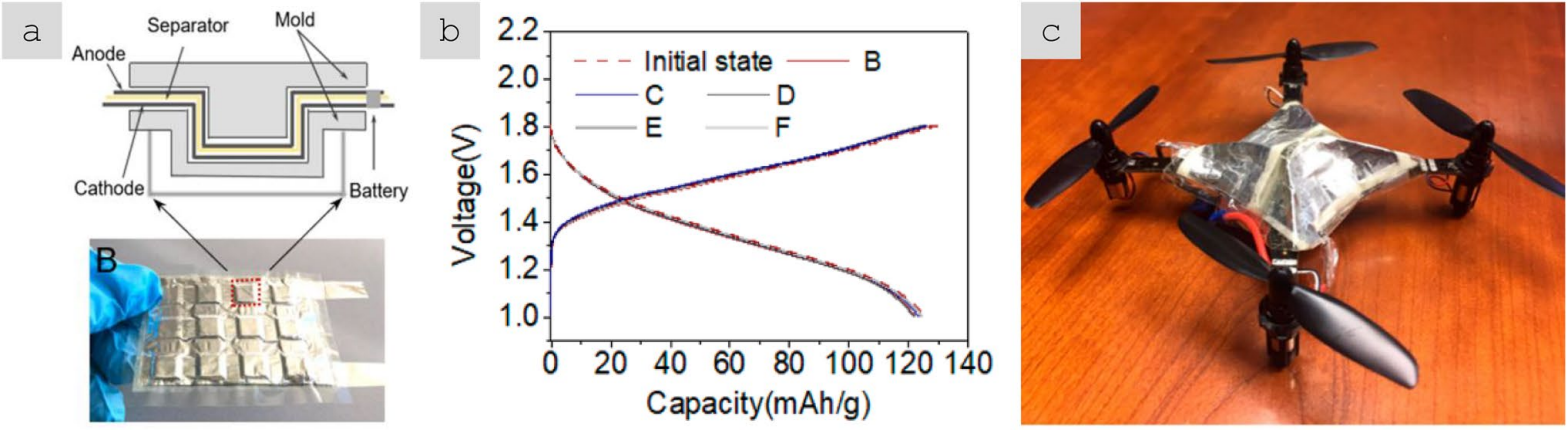
Example of a structural battery enabled by nanoconstituents, including aramid nanofibres (ANF). **a** Structure and photograph of a layered device. **b** Voltage profiles and **c** Example of a UAV with an energy-storing body [227]. **a**–**c** Adapted from [227] with permission. Copyright 2019 American Chemical Society

In addition to more efficient integration of cells, new cell designs are under constant development to reduce the weight of “parasitic” elements. As discussed before, the use of nanocomposite electrodes and lighter nanocarbon-based current collectors are promising strategies in this direction that could produce weight reductions of around 15% wt., with modest changes in battery architecture. As a more ambitious strategy, an attractive new challenge is to produce composites that simultaneously act as structural elements and store energy, termed structural batteries [60, 61, 226]. Combining multiple functions can reduce weight by indirectly eliminating components, thus effectively leading to IF > 1. Figure 30 shows details of a recent example of a structural Zn battery based on aramid nanofibres (ANF) and poly(ethylene oxide) [227], demonstrated in laboratory-scale UAVs (Fig. 30c) and robotic structures [228]. A related strategy is using networks of cellulose nanofibrils for mechanically-augmented battery constituents [229, 230].

### Nanomaterials in supercapacitors

Alongside batteries, integrating supercapacitors as an intelligent and reliable energy source is highly important. They can assist in ensuring that aircraft systems operate reliably and safely and protect the electric generator from abrupt power changes, even in critical situations. Overall, they can help reduce the need for battery replacements and, therefore, improve the overall cost of ownership.

#### Electrochemical energy storage mechanism and interest in nanomaterials

**Fig. 31:**
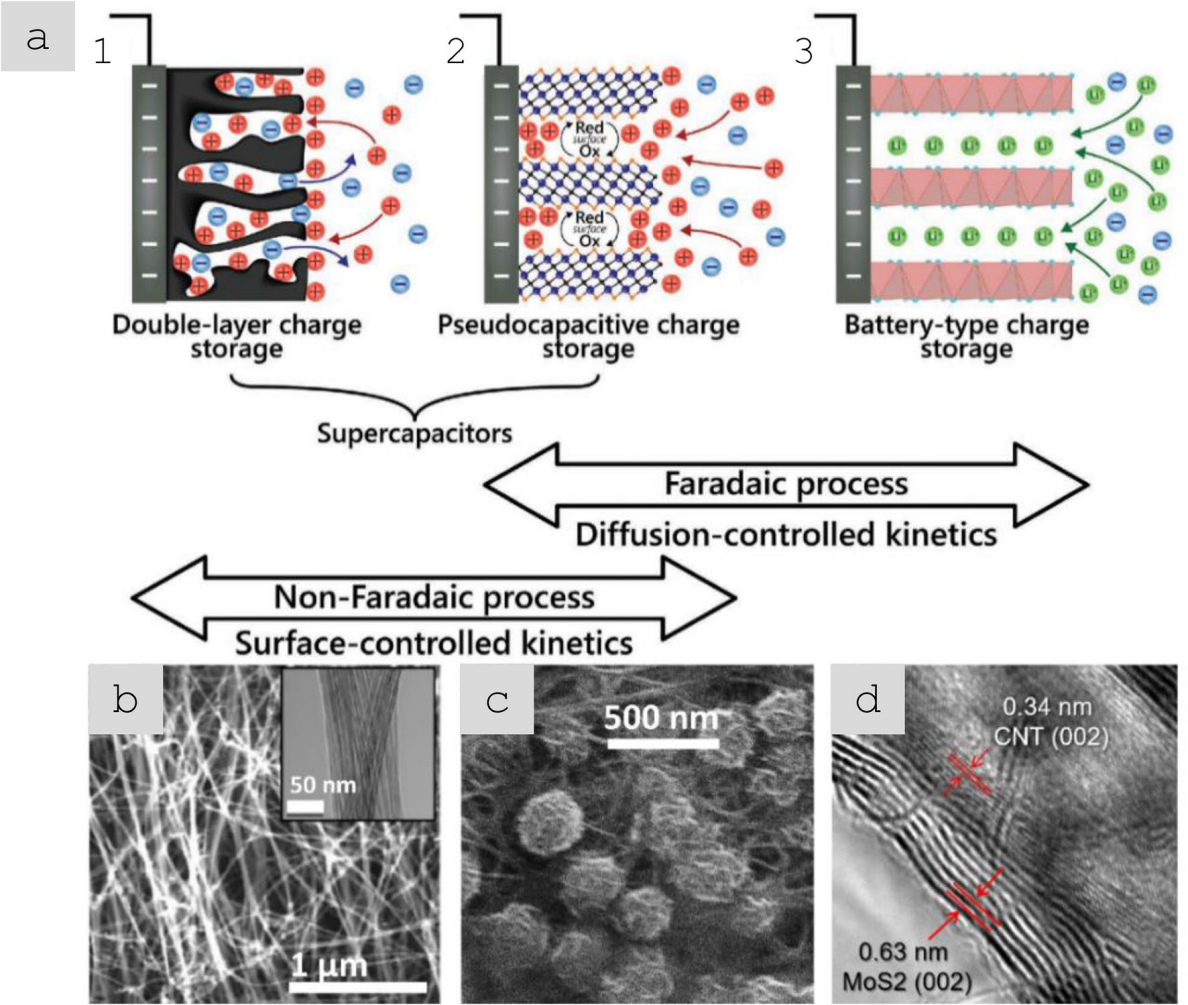
**a** Schematic of different electrochemical energy storage mechanisms [231]: **(1)** electrical double-layer capacitor, **(2)** pseudocapacitive (surface-confined reactions), and **(3)** Faradaic electrodes; and examples of related nanostructured electrodes: **b** CNT network [232], **c** MnO_2_ decorated CNT fibres [233], and (**d** conformal MoS_2_ on CNT fibres [110]. **a** Reproduced from [231] with permission. Copyright 2019 John Wiley and Sons. **b** Adapted from [232] with permission. Copyright 2019 Elsevier. **c** Adapted from [233] with permission. Copyright 2017 Elsevier. **d** Adapted from [110] with permission. Copyright 2021 American Chemical Society

The energy storage mechanism in supercapacitors and its relation to other electrochemical processes is schematically shown in Fig. 31 and briefly reviewed below.

In electrochemical double-layer capacitors (EDLC), electrolyte ions (*i.e.,* charge carriers) are electro-adsorbed on the surface of the electrode material. As EDL formation is through electrostatic accumulation and does not involve any electron transfer at the electrolyte/electrode interface, *i.e.*, the process is non-Faradaic, the charge storage process is highly reversible over long consecutive cycles (e.g., up to 10^6^ cycles) with high Coulombic efficiency (nearly 100%). The kinetics are surface-controlled and much faster than battery-like processes, hence the higher power of EDLCs.

Considering the non-Faradaic nature of EDLCs, the higher the electrode surface, the more charge is stored in the device. The high specific surface area (SSA) of carbon-based materials makes them the natural choice of active material for EDLC. Some examples are included in Table 11. Traditionally, the commercially-used active materials are activated carbons (AC), which can reach SSA above 2000 m^2^/g and specific capacitance (normalised by mass of the active material) of 50–350 F/g. However, most methods that produce high porosity in AC introduce defects in the sp^2^-conjugated network, thus reducing the electrical conductivity of the carbon structure. The interest in nanocarbons stems from their high SSA and high electrical conductivity at the molecular-scale level. The challenge is in developing methods to assemble them into percolated networks with simultaneously high SSA and high electrical conductivity, using processing routes that preserve the high conjugation of the constituents.

**Table 11:** Properties of common carbon materials used in EDLCs and comparison with MnO_2_ as a pseudocapacitive material [232]

Material	σ (S/cm^2^)	SSA (m^2^/g)	C_sp_ (F/g)
AC	10^–4^—10^–1^	800–2300	50—350
Graphene	10^2^—10^5^	100–2630	20—550
RGO	10—10^4^	100–1000	20—250
CNT	10^2^—10^5^	10–1315	10–325
CF	10^2^—10^4^	~ 0.1	< 0.1
MnO_2_	10^–7^—10^–9^	20—320	60–1370

σ: electrical conductivity; SSA: specific surface area; and C_sp_: specific capacitance; AC: activated carbon; RGO: reduced graphene oxide, and CF: carbon fibre

**Fig. 32:**
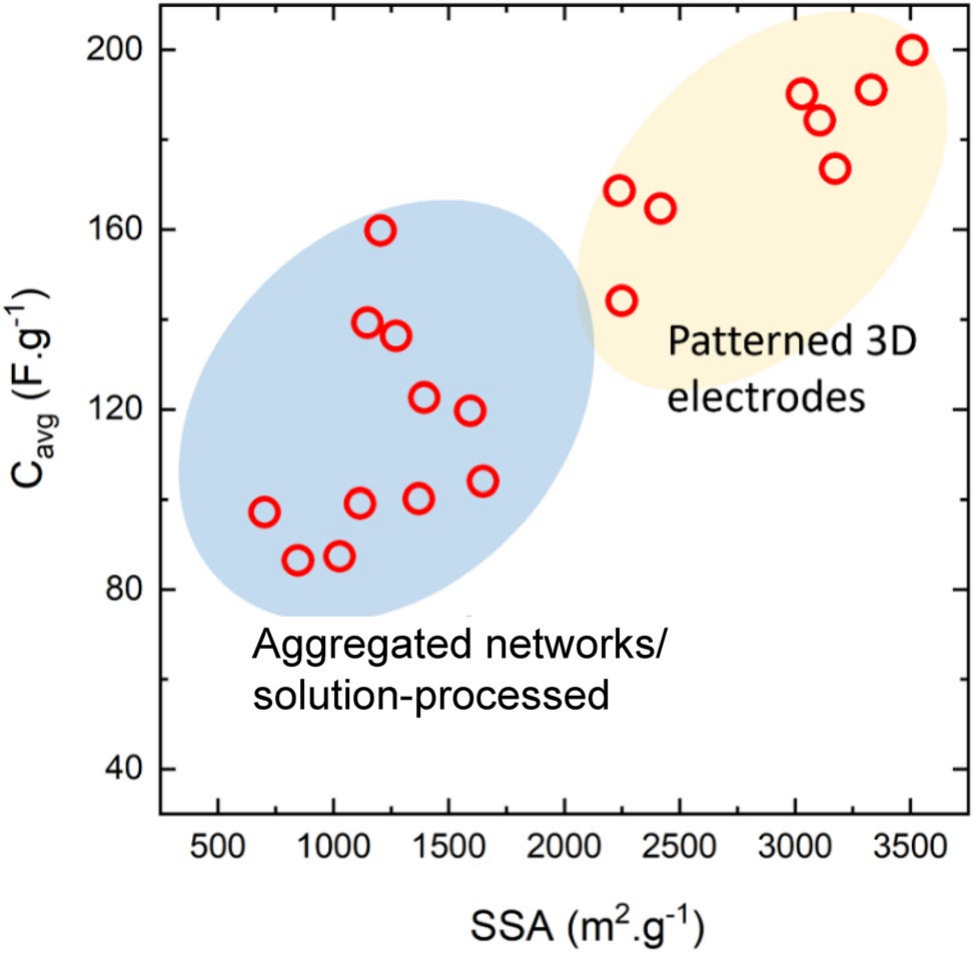
Dependence of specific capacity on specific surface area of nanocarbons in EDLCs [239]. Adapted from [239] with permission. Copyright 2014 Springer Nature

In the absence of defects in the nanocarbons that produce pseudocapacitive reactions and ignore quantum capacitance contributions, the specific capacitance of nanocarbon networks is simply the product of their SSA and the capacitance per unit area of graphite ${C}_{EDLC}={C}_{A}\times SSA$, with ${C}_{A}\cong 8 \mu F/{cm}^{2}$. With purely ELDC storage (porous carbon electrodes without any dopant or functional groups), the large library of nanocarbons available [234] gives access to a wide range of specific capacitances, reaching values above 200 F/g. The examples at the higher end are typically 3D electrode structures with patterns that retain the high SSA of the nanocarbon constituent while ensuring the electrical conductivity of the whole structure. Examples include laser-reduced graphene oxide ensembles [235] or arrays of vertically aligned CNTs [236]. However, the most common method to fabricate nanocarbon supercapacitor electrodes is by the formation of high-aspect-ratio networks, where the available SSA arises due to imperfect packing and electrical conductivity through percolation. The corresponding reduction in SSA from aggregation in these systems limits specific capacitance to around 90 F/g (Fig. 32). Overall, the specific energy density of EDLCs based on nanocarbons (including AC) is in the range of 5–10 Wh/kg in the pack level [237, 238].

#### Pseudocapacitive materials and nanostructured composites electrodes

**Fig. 33:**
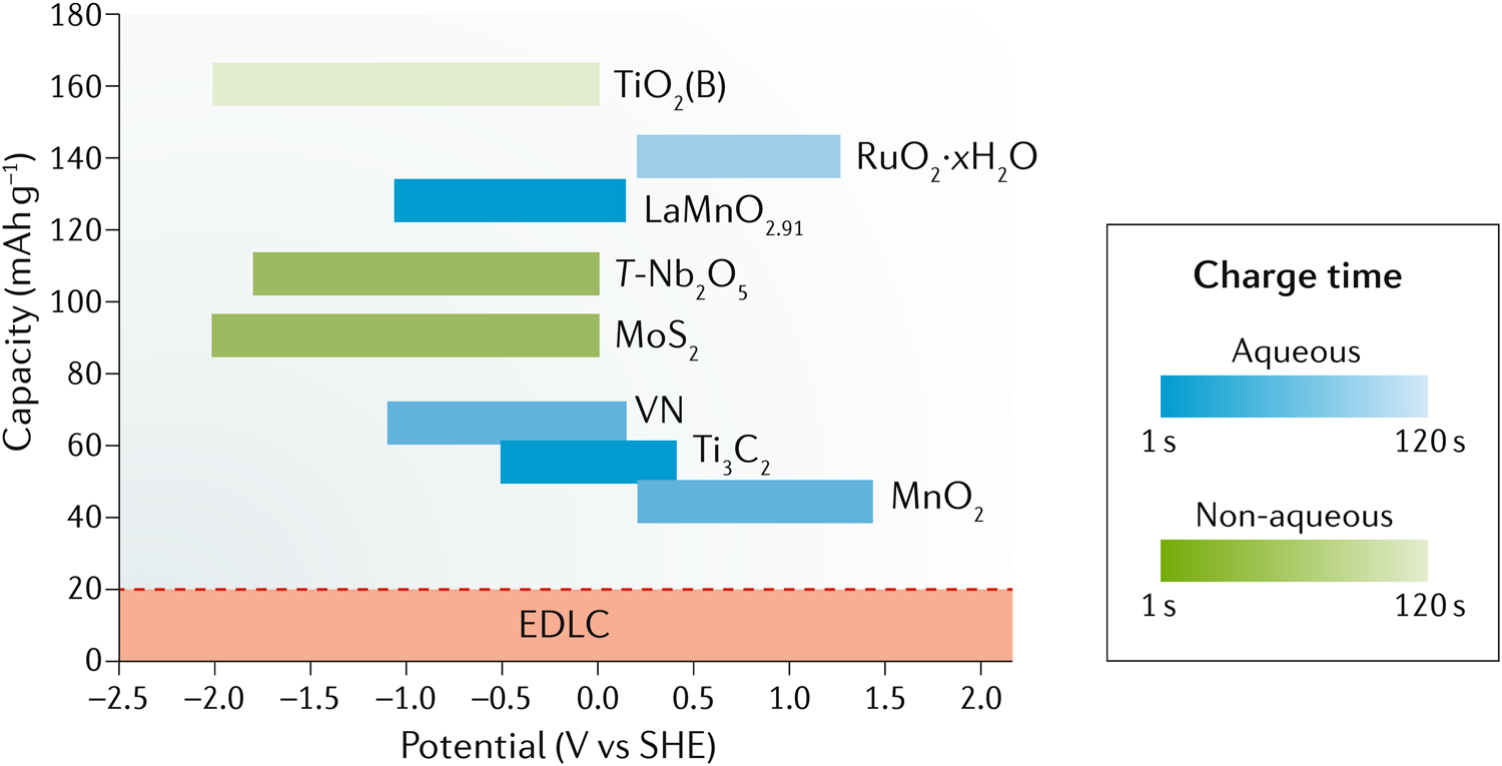
Electrochemical performance of some selected pseudocapacitive material (with a mass loading > 0.5 mg/cm^2^) in aqueous (blue) and nonaqueous (green) media in comparison with benchmark EDLC (~ 20 mAh/g in ~ 1 s). Colour gradient intensity represents the required charging time [238]. Reproduced from [238] with permission. Copyright 2019 Springer Nature

Electrode materials that store energy through Faradic processes, rather than those governed by just physical ion separation, fill the gap of specific energy between the batteries and EDLCs by providing relatively high energy density and high power in the same electrochemical energy storage device. A common type of Faradaic process is surface redox reactions (Fig. 33), which combine order-of-magnitude higher theoretical capacitances (e.g., a theoretical capacitance of 1370 F/g for MnO_2_) than in EDLC nanocarbons, with EDLC-like fast kinetics and are hence termed pseudocapacitive [238]. Figure 33 includes selected examples of pseudocapacitive materials at aqueous and nonaqueous media. An extended list and a detailed discussion of their properties in different electrolyte media can be found elsewhere [238, 240].

The electrochemical properties of intrinsic pseudocapacitive materials such as hydrated transition metal oxides and redox-active polymers have been widely studied for decades [241], and impressively high specific capacitances have been reported (e.g., over 1100 F/g for MnO_2_/LSG electrodes) [242]. We note, though, that most scientific literature reports capacitance values normalised by the mass of the pseudocapacitive material alone and typically for thin electrodes. That may be suitable for understanding the fundamental electrochemical properties of constituent materials but can make it difficult to extrapolate properties to electrodes of mass loading in the range of practical applications. Here, we focus predominantly on reports for “thicker” electrodes (with mass loadings > 3 mg/cm^2^) and for which we can determine capacitance normalised by mass of the whole composite electrode (*i.e.*, including conductive additives and binders, but not current collector). In this regard, research attempts have recently been devoted towards developing novel nonaqueous high-performance pseudocapacitive materials at practical mass loading levels for commercialisation (e.g., > 10 mg/cm^2^). As an example, Sun et al*.* showed that a thick Nb_2_O_5_ (an intrinsic pseudocapacitive material) composite with a 3D holey structure could attain high-rate performance at commercially viable mass loadings [253].  Through electrode composition and structure optimization, they showed minimum capacity loss even at high mass loadings and high rates (e.g., up to 100 ºC). This could be achieved by tailoring the porosity of the 3D conductive scaffold at the nanoscale, which has been found to be essential for optimised charge transport and high-rate energy storage with mass loadings at practical levels.

More recently, there has been strong interest in non-oxide pseudocapactive materials, such as layered carbides/nitrides, termed MXenes. MXenes are 2D hexagonal sheets consisting of an electronically conductive carbide core and a transition metal (M) surface produced from etching the “A” atoms from the MAX phase (where “A” is typically a group 13 or 14 element and “X” is carbon and/or nitrogen) [238]. Having high surface areas and being redox-active (amenable to the intercalation of various ions between the 2D layers) [244], MXenes have high capacitances (e.g., over 900 F/cm^3^ for Ti_3_C_2_T_x_ [245] and a theoretical value up to 1800 F/g for Nb_2_C [246]) in aqueous media. Nevertheless, their electrochemical properties in organic-based electrolytes are less established [238].

**Fig. 34:**
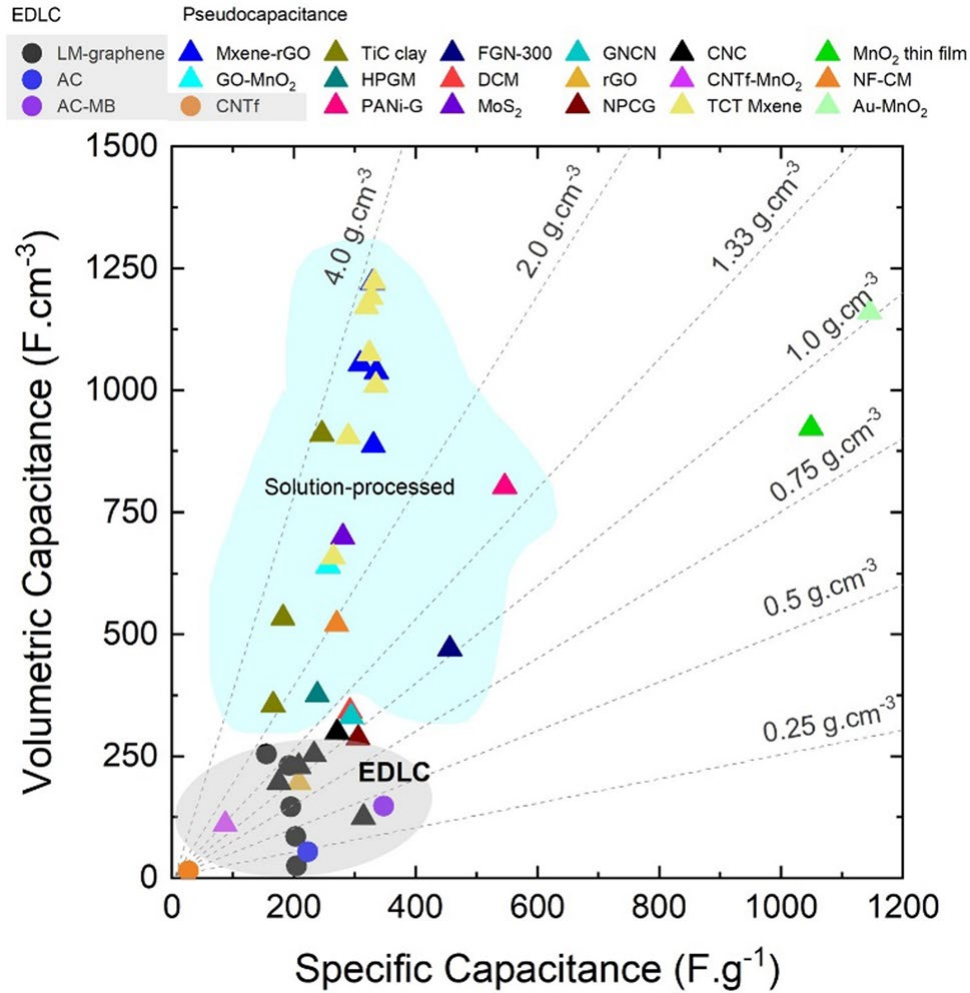
Comparison of the specific and volumetric capacitance of a large pool of materials (EDLC, circles and pseudo-capacitance, triangles) reported in literature. The dashed lines display the apparent density of the electrodes. The grey ellipsoidal shadow shows the aggregation of pure EDLC electrodes. The data have been collected from the ref. [233, 245, 249–267]. Copyright 2024 IMDEA Materials

Overall, nanostructuring of these pseudocapacitive materials has served two main purposes: increasing their SSA to maximise the surface available for redox reactions and decreasing the thickness of the active material to reduce electrical transport resistance from the often-poorly electron-conducting pseudocapacitive materials. From these simple considerations, the performance of pseudocapacitive electrodes is maximised by minimising the size of the active material phase and assembling it over a porous conducting scaffold. The synthesis of composite electrodes consisting of porous networks has been widely pursued by either deposition of the pseudocapacitive material on a pre-formed nanocarbon network (e.g., 1100 F/cm^3^ or 1145 F/g_MnO2_for MnO_2_/Laser-scribed Graphene) [242], or through wet-processing of mixtures of pseudocapacitive nanoparticles and nanocarbons [247] (e.g., 570 F/g for RuO_2_/Graphene Sheets [248]). The success of these strategies can be assessed by considering both the resulting gravimetric and volumetric capacitance, normalised by the whole electrode, including the built-in conducting phase. Selected data are included in Fig. 34. It shows that wet processing gives access to much higher electrode capacitance values. Electrodes combining MXenes and a small amount of nanocarbons have resulted in the highest volumetric capacitance for pseudocapacitive materials. This suggests that assembly from dispersion is more effective at packing the two nanostructured phases as porous networks, probably due to both types of nanoparticles acting as “spacers” preventing excessive aggregation. In pre-formed nanocarbon networks, the initial degree of aggregation is probably too high, with an excess of nanocarbon-nanocarbon interfaces. However, for thicker electrodes, as electrical resistance becomes the limiting process [185], the optimum network structure will require higher through-thickness conduction and, thus, possibly a different structural balance.

It is worth mentioning that in most routes for composite electrode fabrication, the nanocarbons are previously functionalised. This can facilitate the preparation of dispersions and/or improve the interaction and homogeneity of the active nanoparticle distribution near the nanocarbons. Such functionalization usually results in partial surface oxidation of the carbon surface by forming oxygen-containing functional groups (e.g., carboxyl, carbonyl, hydroxyl, epoxy, etc*.*). These functional groups not only improve the adhesion of the nanoparticles on the carbon but also enhance the wettability (hydrophilicity) of the C nanonetwork (resulting in an enhanced impregnation of the aqueous electrolytes) and induce an extra pseudocapacitance caused by redox reactions of the functional groups themselves [232]. However, excessive functionalisation can reduce electrical conductivity, increase self-discharge, and reduce cycle life through electrode corrosion and/or electrolyte decomposition.

**Fig. 35:**
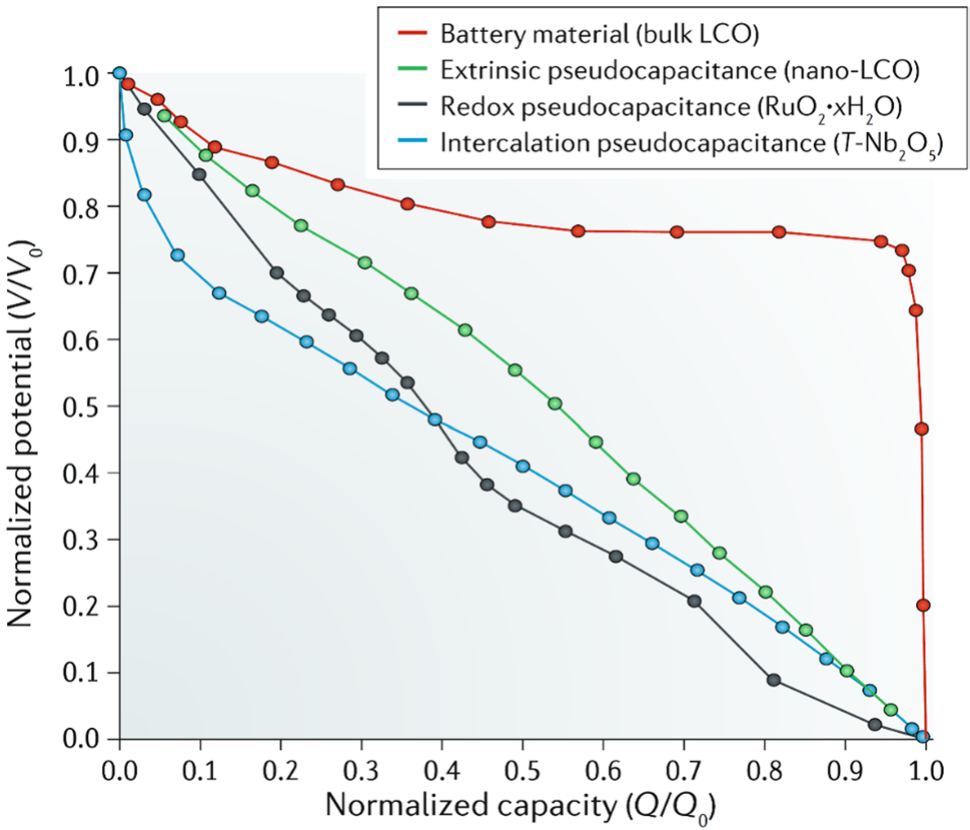
Galvanostatic discharge profiles for various pseudocapacitive materials in comparison with a battery electrode of LiCoO_2_ (bulk LCO) [238]. Reproduced from [238] with permission. Copyright 2019 Springer Nature

In addition to intrinsic pseudocapacitance in materials like hydrated RuO_2_ and MnO_2_, a better understanding of the electrochemical properties of nanoscale materials in recent years led to the appearance of extrinsic pseudocapacitive materials where the electrochemical characteristics of some battery materials change significantly and exhibit a pseudocapacitive response when the size of the particles is nanometric. For some active materials, this can be simply a consequence of their smaller size and correspondingly high SSA, leading to an inherently large pseudocapacitance relative to their diffusion-limited (*i.e.*, battery-like) capacity. An exciting example is layered LiCoO_2_ (LCO), a well-known cathode material for LIBs. Its battery-like voltage profile transforms into a capacitive signal with a linear dependence of charge storage (similar to the intrinsic pseudocapacitive RuO_2_) upon nanostructuring (see Fig. 35), reflecting a different balance of the electrochemical storage process. For other active materials, the charge storage process changes altogether when nanostructured. For instance, reducing the particles of MoO_2_ to 20 nm suppresses kinetically unfavourable phase transitions upon lithiation, resulting in significantly enhanced kinetics of the charge storage process (high-power properties), which could be realised from diminished voltage plateau and going towards a linear profile [268].

In addition to the nanoscale and composite materials, other strategies to stimulate pseudocapacitance, like doping, e.g., in layered materials to increase the interlayer spacing with large moieties, have recently attracted much attention. The doping approach has been examined for materials such as MnO_2_ and Ti_3_C_2_, for which the increase in interlayer spacing can drastically increase ionic diffusion [269, 270].

#### Hybrid devices: metal-ion capacitors

Apart from the hybridisation of the pseudocapacitive and EDLC materials in a single electrode, there has been progress in pairing pseudocapacitive electrodes with EDLC electrodes as hybrid supercapacitors. In such systems, a purely EDLC electrode (e.g., carbon) is often integrated with a redox-active battery-like electrode, such as conducting polymers or metal oxides. Although all early efforts have been centred on the development of aqueous-based hybrid devices (e.g., based on MnO_2_ and RuO_2_), they have not been successful in surpassing the EDLC performance (e.g., 5 Wh/kg at 10 kW/kg based on the total mass of the device), but they laid the groundwork for further development of the nonaqueous hybrid devices with ~ 4 V (e.g., high specific energy).

In this regard, one of the promising fast-advancing devices is the metal-ion capacitor. In such devices, an intercalation electrode is often integrated with a purely EDLC electrode (e.g., AC). In a lithium-ion capacitor (LIC), where a Li-ion battery anode (e.g., Li_4_Ti_5_O_12_) is paired with an AC cathode, a gravimetric energy density of ~ 3–4 times greater (e.g., 40 Wh/kg) than that of EDLC is achievable over 10,000 of cycles [271, 272]. The use of graphite anode increases the specific energy of LICs up to 60 Wh/kg, although they encounter the pre-lithiation issue [273]. In the past 20 years, tremendous efforts have been devoted to developing metal ion capacitor electrodes and promising advances have been made. For instance, in an all-nanocarbon architecture, a high specific energy of over 250 Wh/kg (based on active material) has been achieved using 3D open-porous interconnected partially rGO cathode and ultralong open-end N-doped carbon nanopipes [274]. Employing the conversion mechanism, Cao et al*.* have shown that a high maximum specific energy of 227 Wh/kg (on the material basis) can be achieved by using MnO anode consisting of homoepitaxially aggregated MnO nanocrystals with percolated porous channels [275].

#### Structural supercapacitors

**Fig. 36:**
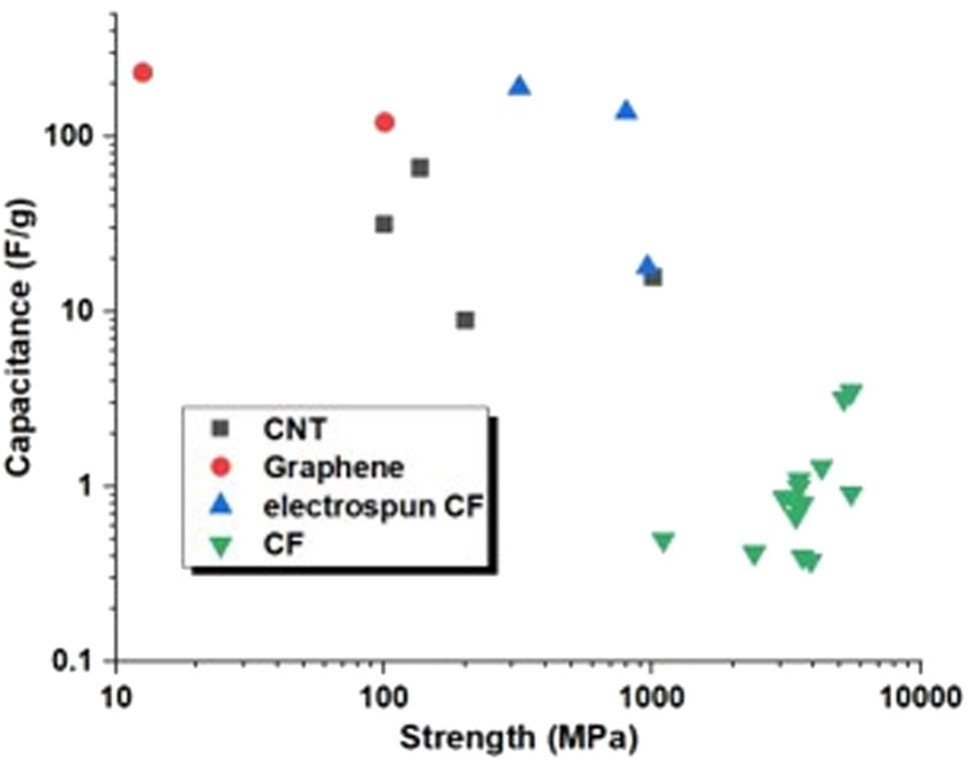
Multifunctional properties of carbon materials for structural supercapacitors. Copyright 2024 IMDEA Materials

Integrating the carbon and pseudocapacitive materials is even more relevant when other functionalities (e.g., those requiring mechanical and flexible properties) of the nanocarbon networks in the electrode are brought to the device. Accordingly, apart from improving the electrochemical properties of supercapacitors, research efforts over the last years have also focused on introducing new materials and processes leading to mechanically augmented electrodes and, in the bigger picture, in full devices. In order of complexity and difficulty, they may be flexible, stretchable, tough, and ultimately act as load-bearing structural elements [60, 61, 232]. As mentioned previously, the ability to produce networks of materials based on high-aspect-ratio conducting nanocarbons leads to an unusual combination of high surface area and electrical/mechanical properties of interest for multifunctional composites. By illustration, Fig. 36 presents a plot of capacitance against tensile strength for different carbon materials. The comparison shows the area of combined performance to be populated by nanostructured materials (including electrospun nano/micro CFs [276]).

**Fig. 37:**
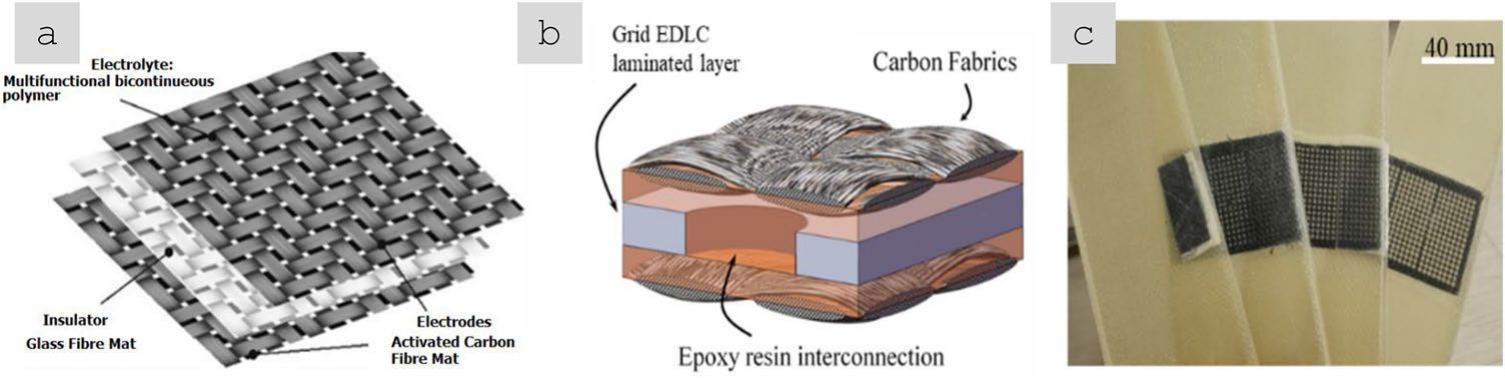
Structural supercapacitor composite architectures: **a** laminated structure with a nanoporous monolithic carbon phase as active material and stiff matrix [278]; **b,c** energy-storing interleaves with patterns for lamina interconnection through structural resin plugs [279, 280]. **a** Reprinted from [278] with permission. Copyright 2013 Elsevier. **b** Adapted from [279] under the Creative Commons CC-BY license. **c** Reprinted from [280] with permission. Copyright 2022 Elsevier

Composites based solely on nanomaterials for mechanical reinforcement currently have mechanical properties in the range of thermoplastics [277]. Composites with properties in the structural range require hybrid solutions, most often combining FRPs with nanomaterials. An important factor in such laminated structural power composites is the matrix, which controls ionic conductivity and several composite mechanical properties (e.g., compressive stiffness) and represents a significant challenge for manufacturing and properties. There are broadly two methods to integrate nanomaterials in FRP compatible with a subsequent formation of a stiff matrix: a) formation of a nanoporous monolithic structure around the CFs that acts as both active material and matrix scaffold [226], and b) introduction of nanostructured interleaves with patterns enabling the flow of structural resin to join lamina. Examples of these two strategies are included in Fig. 37. In Table 12, we summarise the properties of structural supercapacitor composites of selected examples predominantly containing nanomaterials.

**Table 12:** Mechanical and electrochemical properties of structural supercapacitor laminates

Electrode material	Electrolyte	Capacitance (mF/g)	Energy density (mWh/kg)	Power density (W/kg)	Mechanical properties
CF-based EDLC interleaf in FRP (no nanomaterials) [281]	H_2_SO_4_ + PVA	0.87(a)	0.08(a)	0.0092(a)	S = 192 MPa M = 9.3 GPa
CNT fibre-based interleaves in CFRP [279]	PVDF-HFP + PYR14TFSI	88(b)	37.5(b)	30(b)	S = 153 MPa M = 60,000 MPa
CF + nanoporous C matrix [282]	PEGDE + 10% IL	602(c)	1(c)	2.68(c)	S_shear_ = 8.7 MPa M_shear_ = 895 MPa
CNT/ABA/Polyaniline modified CF [283]	CF_3_SO_3_Li + PEG copolymer	125(d)	17(d)	-	S = 21 MPa M = 2900 MPa

S: flexural strength; M: flexural modulus; S_shear_: shear strength; M_shear_: shear modulus. Normalised by the hybrid composite total weight (a), by the total weight of EDLC interleaf (b), by the electrode weight (c), and by the total cell weight of electrodes, electrolyte, and separator (d).

#### Supercapacitors in aviation

Their high specific power and long cyclability (10^5^–10^6^ cycles) make supercapacitors suitable for the management of electric power in the electric network, which is critical in future MEAs. Despite their limited energy densities, EDLC supercapacitors are highly attractive in transportation applications for burst and/or regenerative power due to their fast kinetics (high power) and small/independent internal resistance to depth of discharge (DoD). They are ideal high-power sources in combination with batteries and are currently used in hybrid/electric vehicles. For instance, municipal buses supply electricity to open/close the doors as well as help the vehicle’s initial acceleration while being charged during braking. In the Airbus A380, supercapacitors can open/close the aircraft's heavy doors in an emergency independently of the central power system [284]. It is envisioned that supercapacitors will be increasingly integrated into future aircraft for powering new electronics, such as autonomous wireless sensor networks [285], high-power actuators for altitude control [286] and activation of electromechanical conduits, as well as their use to compensate the battery for power applications [287]. However, the main opportunities are probably in smaller aircraft, including UAVs, and particularly for lift-off/take-off [288, 289]. To enable assessment of their potential in such applications, in Table 13, we include metrics for high-performance supercapacitive energy storage devices with nanostructured electrodes that we consider amongst the more mature for implementation in aviation. These selected examples combine demonstration of electrodes produced using scalable processes, electrodes of significant mass loading, and electrochemical tests in full cells.

**Table 13:** Selected examples of high-performance electrochemical energy storage nanostructured systems

System	Electrode composition	Electrolyte	Energy density	Power density	References
Pure EDLC	Activated Graphene-based AC	[EMIM][TFSI]/AN	16 Wh/kg(a)	7 kW/kg(a)	[290]
Pure EDLC	Liquid-mediated Graphene	1 M H_2_SO_4_	60 Wh/L(a)	~ 10 W/L(a)	[259]
Pseudocapacitive composite electrodes	Graphene/MnO_2_	1 M Na_2_SO_4_	31 Wh/kg(b)	100 W/kg(b)	[291]
Lithium Ion Capacitors	AC (+ sacrificial salt)/Graphite	1 M LiPF_6_ in EC:DMC	~ 60 Wh/kg(c)	50 W/kg(c)	[273]

(a) The values are at cell level, (b) at active material level, and (c) at the electrode level.

### Nanomaterials in Fuel cells

#### Hydrogen technologies

**Fig. 38:**
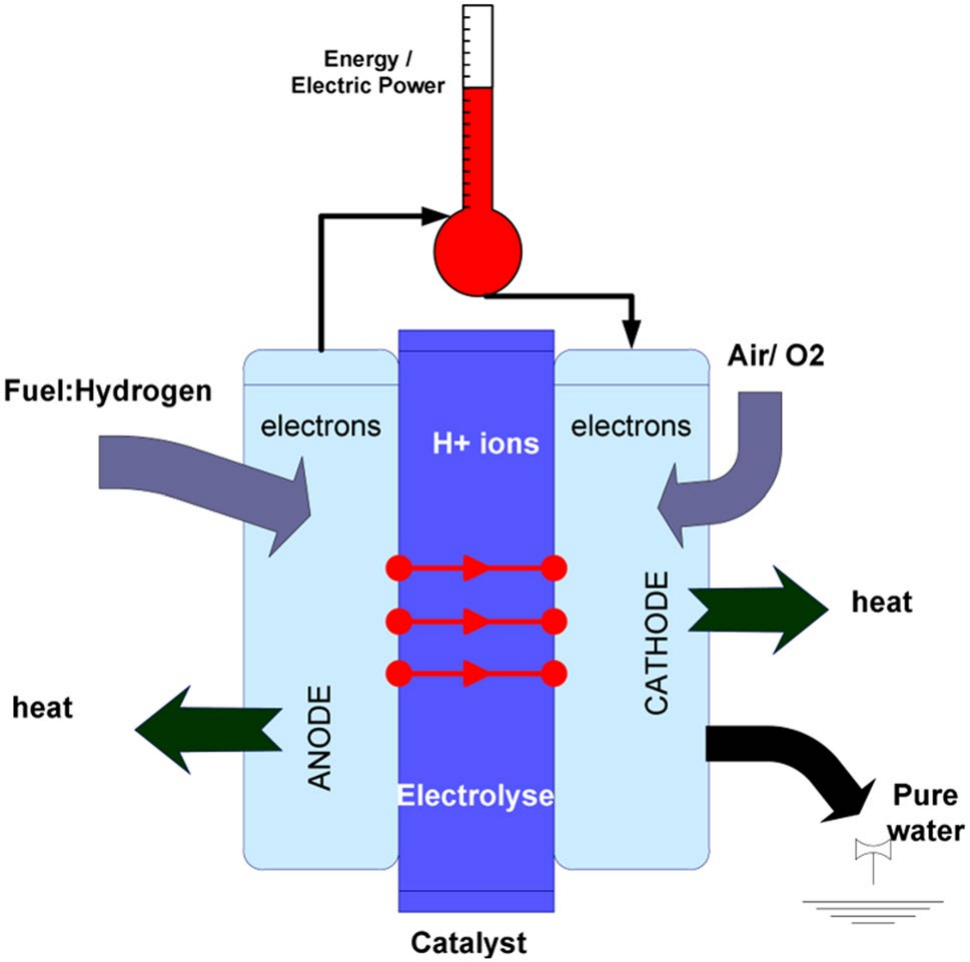
Schematic illustration of a fuel cell [292]. Reproduced from [292] with permission. Copyright 2006 European Organisation for the Safety of Air Navigation EUROCONTROL

**Fig. 39:**
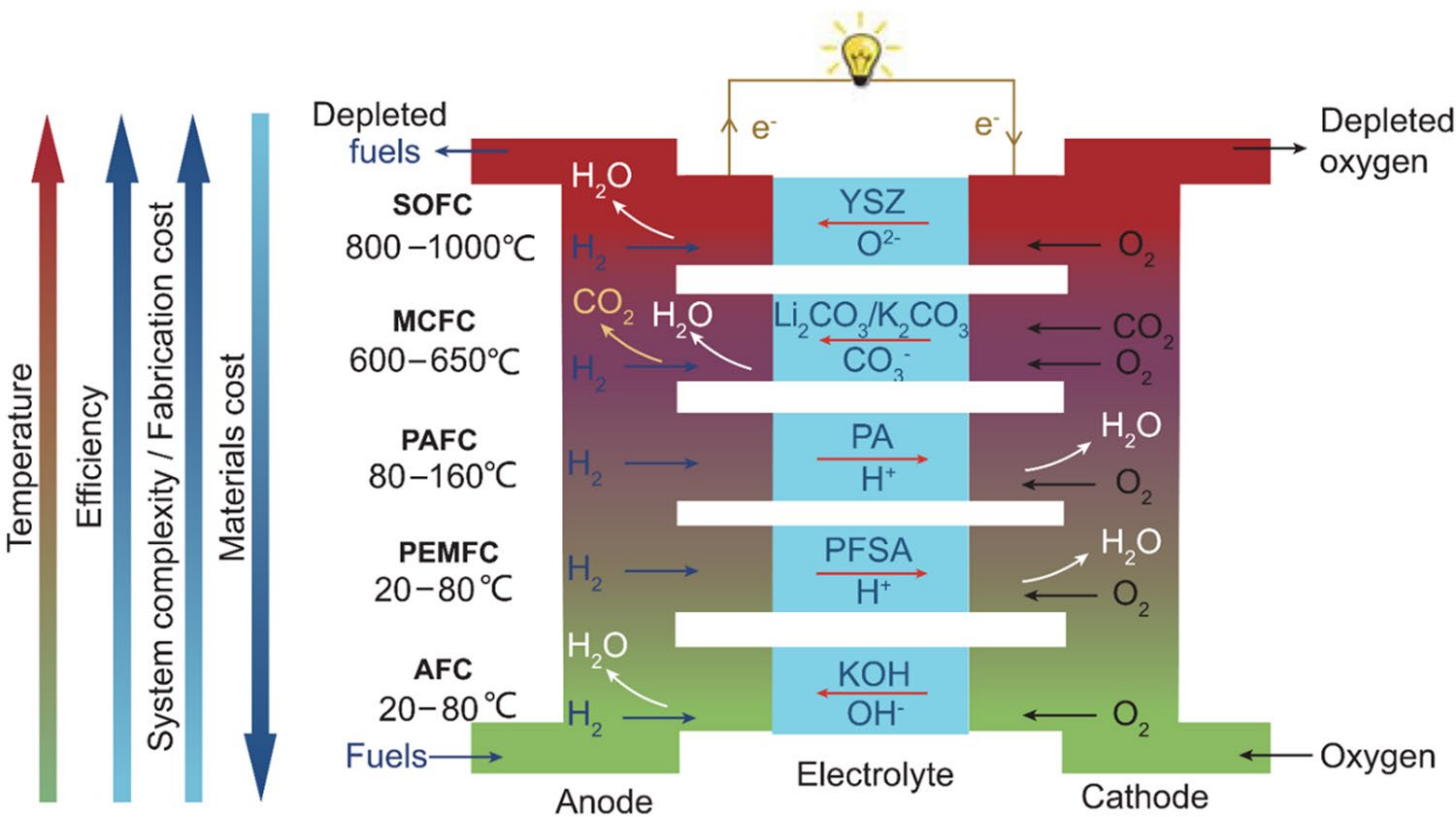
Schematic representation of various fuel cell types, showing their operation temperature range, common electrolytes as well as the general trend for their efficiency, complexity, fabrication, and material cost. SOFC: Solid Oxide Fuel Cell; MCFC: Molten-Carbonate Fuel Cell; PAFC: Phosphoric Acid Fuel Cell; PEMFC: Proton-Exchange Membrane Fuel Cell; AFC: Alkaline Fuel Cell; YSZ: Y_2_O_3_-ZrO_2_ electrolyte; PA: Phosphoric Acid; PFSA: Perfluorosulfonic Acid [294]. Reprinted from [294] under the Creative Commons CC-BY license

Fuel cells (FCs) are among the oldest electrical conversion technologies, dating back to the nineteenth century. Similar to batteries, FCs convert chemical to electrical energy through electrochemical reactions, but unlike batteries, FCs do not require charging and can operate as long as the fuel is supplied. Most simply, they work by oxidation of H_2_ (as fuel) in the anode and reduction of O_2_ (from air) in the cathode (Fig. 38). The application of FCs extends from small-medium mobile electric generators to large stationary units. They are commonly classified based on the electrolyte type (shown in Fig. 39 and Table 14).

**Table 14:** Summary of the parameters corresponding to various fuel cell systems and their application [293]

Parameters	Fuel cell types					
	PEMFC	AFC	PAFC	MCFC	SOFC	DMFC
Electrolyte	Solid polymer membrane (Nafion)	KOH solution	H_3_PO_4_	Lithium and potassium Carbonate (LiAlO_2_)	Solid Oxide electrolyte (Y_2_O_3_, ZrO_2_)	Solid polymer membrane
Operating temperature (ºC)	50–100	50–200	~ 200	~ 650	800–1000	60–200
Power density (kW/m^3^)	3.8–6.5	~ 1	0.8–1.9	1.5–2.6	0.1–1.5	~ 0.6
Capacity	30 W—250 kW	10–100 kW	100 kW –1.3 MW	155 kW—2 MW	1 kW—1.7 MW	1W—1 MW (Research)
Applications	Residential; UPS; emergency services; transportation	Transportation; space shuttles; portable power	Transportation; commercial cogeneration; portable power	Transportation; industries; utility power plants	Residential; utility power plants; commercial cogeneration; portable power	Replacement of batteries in portable devices
Advantages	High power density; quick start up; solid non-corrosive electrolyte	High power density; quick start up	Produce high grade Waste heat; stable electrolyte characteristics	High efficiency; no metal catalysts needed	Solid electrolyte; high efficiency; generate high grade	Reduced cost due to absence of fuel reformer
Drawbacks	Expensive platinum catalyst; sensitive to fuel impurities (CO, H_2_S)	Expensive platinum catalyst; sensitive to fuel impurities (CO, CO_2_, CH_4_, H_2_S)	Corrosive liquid electrolyte; sensitive to fuel impurities (CO, H_2_S)	High cost; corrosive liquid electrolyte; slow start up; intolerance to sulfur	High cost; slow start up; intolerance to sulfur	Lower efficiency and power density

The properties of FCs, including high electric efficiency, low emissions, and silent operation, are highly attractive to transport applications, especially in aviation. However, the weight and complexity of associated H_2_ storage systems are major challenges.

#### How can nanotechnology improve fuel cells?

**Fig. 40:**
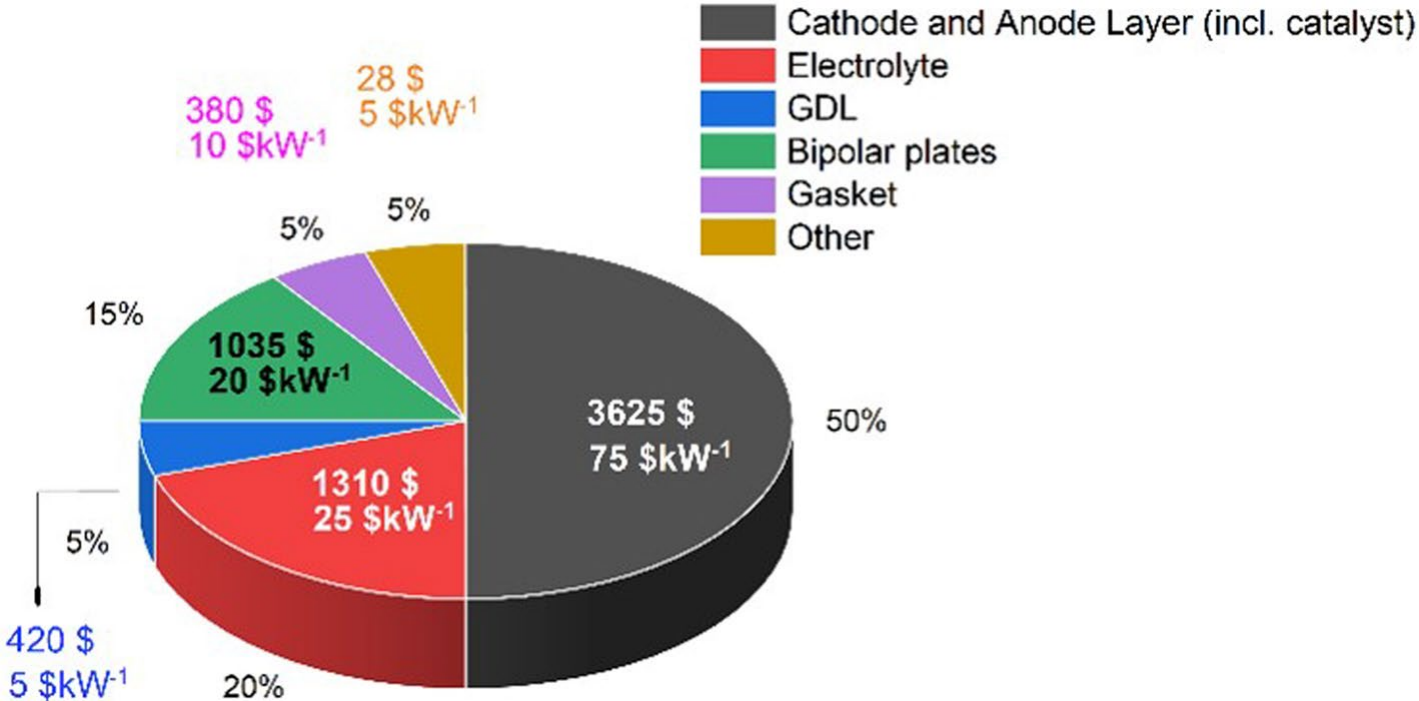
Breakdown of fuel cell stack costs [292]. Adapted from [292] with permission. Copyright 2006 European Organisation for the Safety of Air Navigation EUROCONTROL

The operation of the FCs is strongly related to the electrode catalysts involved in the redox reactions. Platinum is the most active catalyst for fuel cells fed with hydrogen, reformate or methanol [295]. However, using bulk Pt as the catalyst drastically increases the cost of the FCs (see Fig. 40). Reducing Pt content or replacing it altogether with nanostructured catalysts has been a constant goal.

Nanostructuring can enhance the active surface area of the precious metal catalyst (e.g., Pt), leading to enhanced activity and, therefore, reduced mass loadings. Dispersing the Pt nanoparticles on carbon black support is the most common method to lower the catalyst use and, hence, price. In this fashion, the surface chemistry of the catalyst and the strength of catalyst-support interactions can be tuned through nanoengineering to get desirable dispersion and electronic characteristics needed for the electrocatalytic reaction. On the other hand, the hydrogen stream is not often pure as it includes CO_2_ and CO (e.g., H_2_ obtained from the reformation process), which poison the Pt and deteriorate the electrical efficiency and power density of the FC. Nevertheless, the CO tolerance of the catalyst can be enhanced through alloying Pt with Ru, Sn, or Mn, and it has been shown that the best performance can be obtained with Pt-Ru electrocatalysts with a particle size of 2–3 nm [295]. Another approach to enhance the surface area of the catalyst is the fabrication of porous electrocatalyst films. One effective strategy is de-alloying, whereby nanoporosity is formed through selective dissolution of one of the phases [296]. This method is also often used to obtain specific surface composition layers [297], like the Pt_3_Ni1 {111} system [298].

Apart from the focus on enhancing the performance of the Pt-based catalysts, extensive efforts have also been devoted to developing non-precious catalysts (e.g., those without Pt), such as nanostructured metal oxides [299] or nanocarbon catalysts [300]). In addition to the improved catalytic properties using nanostructured catalysts, employment of the nanocarbons with high crystallinity (e.g., CNTs) as the support (instead of commercial Vulcan XC-72) has been demonstrated to be an effective strategy towards carbon corrosion issues, usually seen in (start up/shut down) conditions [301].

In addition to the catalyst, the development of nanostructured materials is also impacting fuel reformation and hydrogen storage, as well as the fabrication of membrane-electrode assemblies (MEA). The aim is to make more efficient membranes that can result in lighter and more durable fuel cells. The membrane pore structure and its transport characteristics are interrelated through properties like conductivity, diffusion, permeation, and electroosmotic drag. Nanostructuring offers new methods to modify these properties [302], for example, through a combination of organic polymers with different nanofillers to form hybrid membranes. For instance, CNTs added to the common Nafion membrane enhance the mechanical and thermal stability of the membrane [303]. The homogenous dispersion of MWCNT in a Nafion matrix through the melt processing method demonstrated 60% lower methanol permeability and 160% higher young modulus compared to a less homogeneous dispersion obtained by ball milling [304]. Also, using functionalized CNTs (with polysiloxane agents) not only helps better filler dispersion but also enhances the proton conduction in the membrane [305]. Beydaghi et al*.* evaluated a graphene oxide (GO) based nanofiller in composition with Fe_3_O_4_ nanosheets for DMFC applications. Accordingly, they showed an addition of 5% wt. GO/Fe_3_O_4_ nanosheets improved membrane properties in terms of methanol permeability, that is, with a decrease from 1.78 × 10^–6^ to 8.83 × 10^–7^ cm^2^/s caused by good inhibitor properties of GO [306]. As another example, the addition of nanoceramic (e.g., SiO_2_, ZrO_2_) fillers in the polymer electrolyte network can reduce fuel cross-over in perfluorosulfonic membranes [307, 308].

#### The application of fuel cells in aviation

**Fig. 41:**
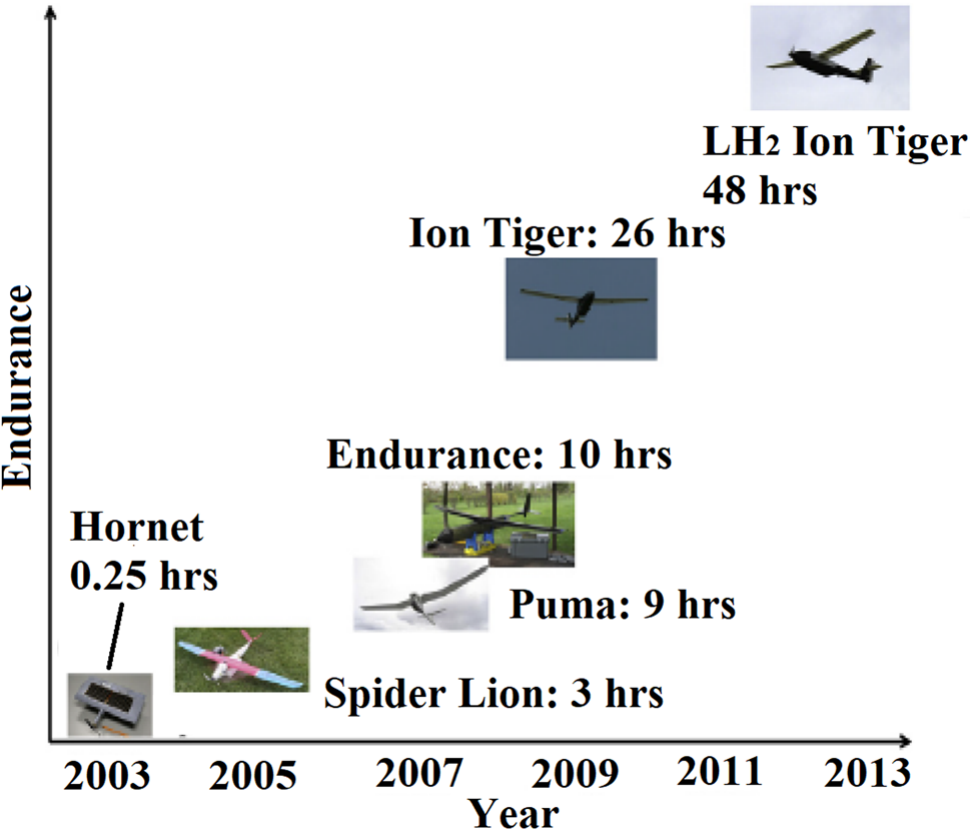
Historical evolution of endurance of FC-powered UAVs. Reproduced from [311] with permission. Copyright 2017 Elsevier

Almost two decades have passed since the appearance of the first fuel-cell-powered UAVs and five years since the first 4-seat passenger aircraft powered exclusively by FCs [309]. Advances in the endurance of FC-powered UAVs (see Fig. 41) indicate the timeline and early progression in FC propulsion. For mid-range commercial aircraft, in addition to the interest in hybrid propulsion, there are various additional functions identified in the short term by replacement of on-board power sources with silent and more efficient FCs: emission-free ground operations, electrical main engine start, electrical environmental control system, water generation for on-board purposes, heat generation (for de-icing purposes), and humidification of the cockpit and/or cabin [310].

**Fig. 42:**
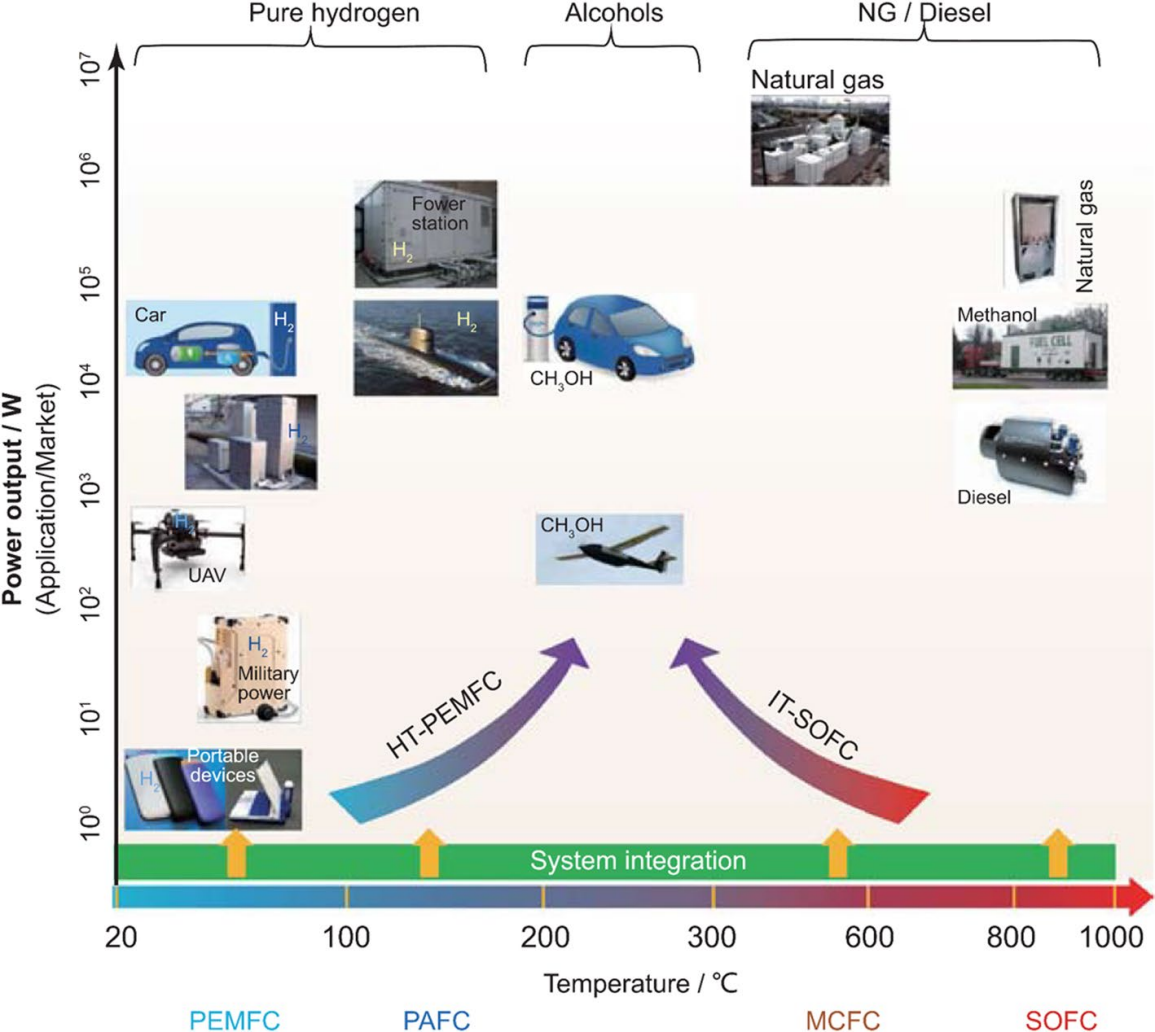
Schematic presentation of application and power range of the main FCs as a function of their operational temperature [294]. Reprinted from [294] under the Creative Commons CC-BY license

Figure 42 schematically illustrates the application and power range of the main FCs. Among all the different types of FCs, SOFCs and PEMFCs are the most studied for aircraft technology. This is reflected in the R&D trend (the arrows in Fig. 42) in the development of high-temperature PEMFCs (HT-PEMFCs) and intermediate-temperature SOFCs (IT-SOFCs). The operating temperature and fuel processing of the SOFCs are theoretically and conceptually suitable for hybrid configuration with the gas turbines in the aircraft. Boeing has conducted several investigations for hybridisation of the SOFC and micro gas turbines, conceptually demonstrating the significant benefit for high-temperature systems [312–314]. SOFC processing methods and related systems have also been developed by Airbus [315, 316]. However, SOFCs still seem far from aircraft requirements and significant improvement in their power density is needed. On the other hand, PEMFCs are more advanced in TRL in the automotive industry, and therefore, they have a better chance of being promising candidates to be integrated into aeronautic applications. In this regard, Boeing has demonstrated a small manned 2-seat prototype glider aeroplane powered by a hybrid PEMFC and LIBs.

Despite this progress, there are several challenges to the operation of FC in aviation (in addition to new requirements for infrastructure and systems [20]). For example, in the case of SOFCs, the key challenges are the system’s thermal stability and stack durability. This opens different opportunities for nanomaterials for thermal management and structural materials for LH_2_ storage and handling (briefly discussed below). It also implies a big gap in projecting the performance metrics from laboratory-scale FCs (e.g., often tested in micro or even nanoscale) to the final device solution, particularly for aerospace applications, which are orders of magnitudes larger and operating in conditions far from the lab. In the case of PEMFCs, the most advanced proton-exchange membranes (*i.e.*, PFSA-based) are essentially only suitable for low operation temperatures (80 °C or lower). However, high-temperature operation is desirable for high energy efficiency, high impurity tolerance and better heat/water management. In addition, although massive research activities have been devoted to the development of non-precious metal electrocatalysts, the majority of them perform reasonably only under alkaline medium (aiming AEMFCs) and are not suitable for PEMFCs. Therefore, considering any commercially viable application of PEMFCs in aerospace would depend on considerable R&D effort in developing the key materials (*i.e.*, the electrocatalysts, membranes, and corrosion-resistant bipolar plates) working at high-temperature operation. Although, in recent years, great advancements in improved Pt-based catalyst performance with low mass loading have been achieved, long-term durability at high temperatures still remains the main challenge [317].

In the case of SOFCs, the R&D trend is to reduce the temperature range to 400–700 °C to avoid electrode/stack degradation, decrease the cost of cell fabrication, and increase thermal stability while maintaining the ability to operate on hydrocarbon fuels. However, the development of IT-SOFCs is tied to introducing novel electrolytes with high ionic conductivity and high-performance electrodes at such reduced temperatures [318]. Nevertheless, the advance of SOFC in lowering its operating temperature has opened up new prospects for applying nanomaterials. For instance, the Sn-doped Ni/YSZ anode [319] has been investigated at 650 °C for 137 h, providing a power density of 410 mW/cm^2^. Overall, further advancement is required in the case of both HT-PEMFCs and IT-SOFCs systems while considering more realistic scenarios regarding the final application so that their commercially viable integration could be realised in aerospace.

## Structural properties

### Revisiting reinforcement of structures

**Fig. 43:**
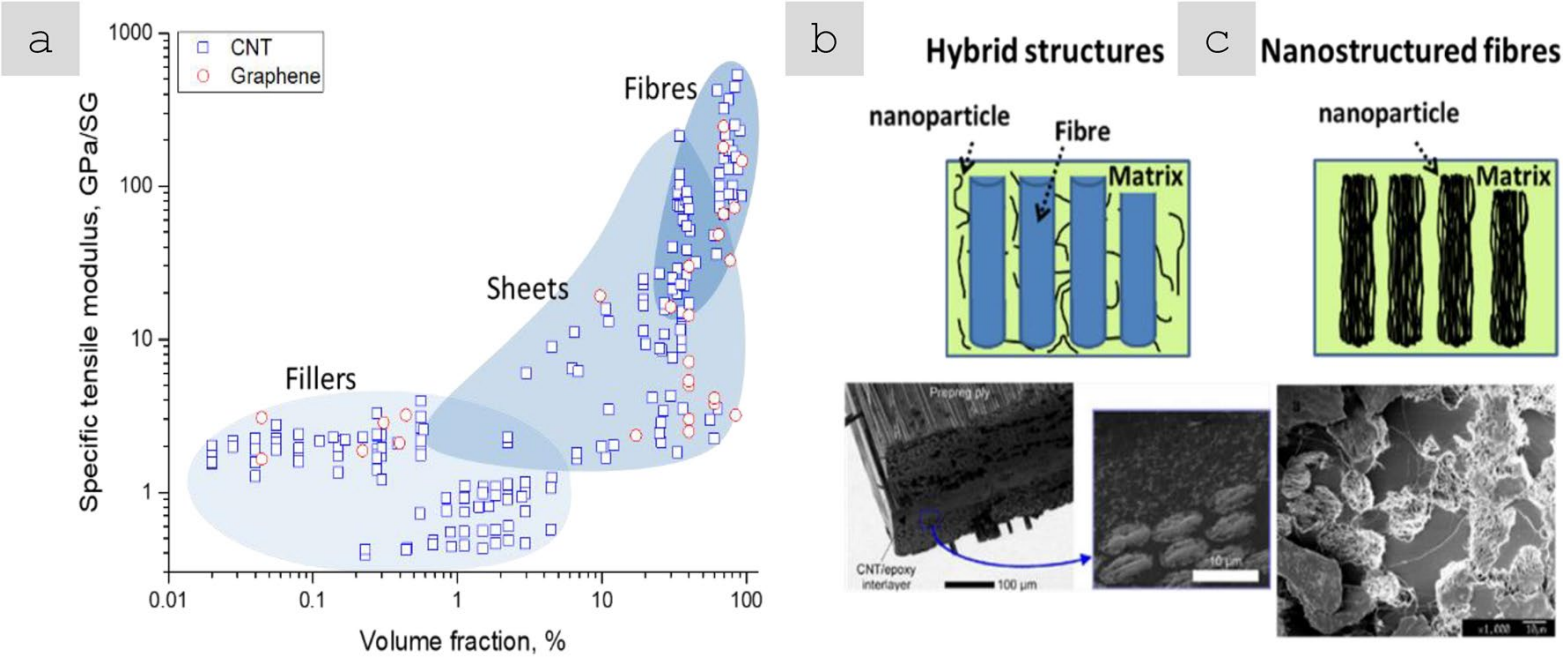
Mechanical reinforcement with high-aspect ratio carbon nanomaterials. **a** Specific tensile modulus of the main embodiments of CNTs and graphene, showing the increase in load transfer with improved packing of elements. **b,c** Different routes to use nanomaterials in aeronautical composites: as fillers for matrix reinforcement, as sheets or aligned arrays [326] for interlaminar reinforcement and as reinforcing fibres [327]. **a** Copyright 2024 IMDEA Materials. **b** Adapted from [326] with permission. Copyright 2008 Elsevier. **c** Reproduced from [327] with permission. Copyright 2009 Elsevier

The application of nanomaterials, particularly nanocarbons (CNTs and graphene), for improving the mechanical properties of aerospace structural materials has received vast attention over the last decades. As observed for electrical and thermal properties, a dominant factor for bulk mechanical properties is the volume fraction of nanomaterials achieved. Figure 43a shows this as a plot of specific tensile modulus against volume fraction. Note, for fillers, density is taken as that of the nanocomposites; the data for sheets and fibres is without any matrix; volume fraction is calculated as the ratio of their apparent density over the theoretical density of 1.8 g/cm^3^, assuming close packing of CNTs.

Polymer matrices with randomly dispersed fillers peak at a specific tensile modulus around 1–3 GPa/SG. Most sheets have a modulus of 1- 30 GPa/SG in the plane, with some exceptionally high values shown for composites with stretched sheets with maximised alignment (approaching 100 GPa/SG) [320, 321] or with stretched and functionalized sheets (200 GPa/SG) [322]. Aligned fibres have longitudinal modulus from 10 GPa/SG to values above 200 GPa/SG [323, 324], which approach values for single crystals of the constituents and high-modulus CF. The main formats to integrate these materials into aeronautical components have been as hybrid structures of nanomaterials and FRPs [325] and as nanostructured fibres to replace CF altogether (Fig. 43b,c).

#### Hybrid structures

**Fig. 44:**
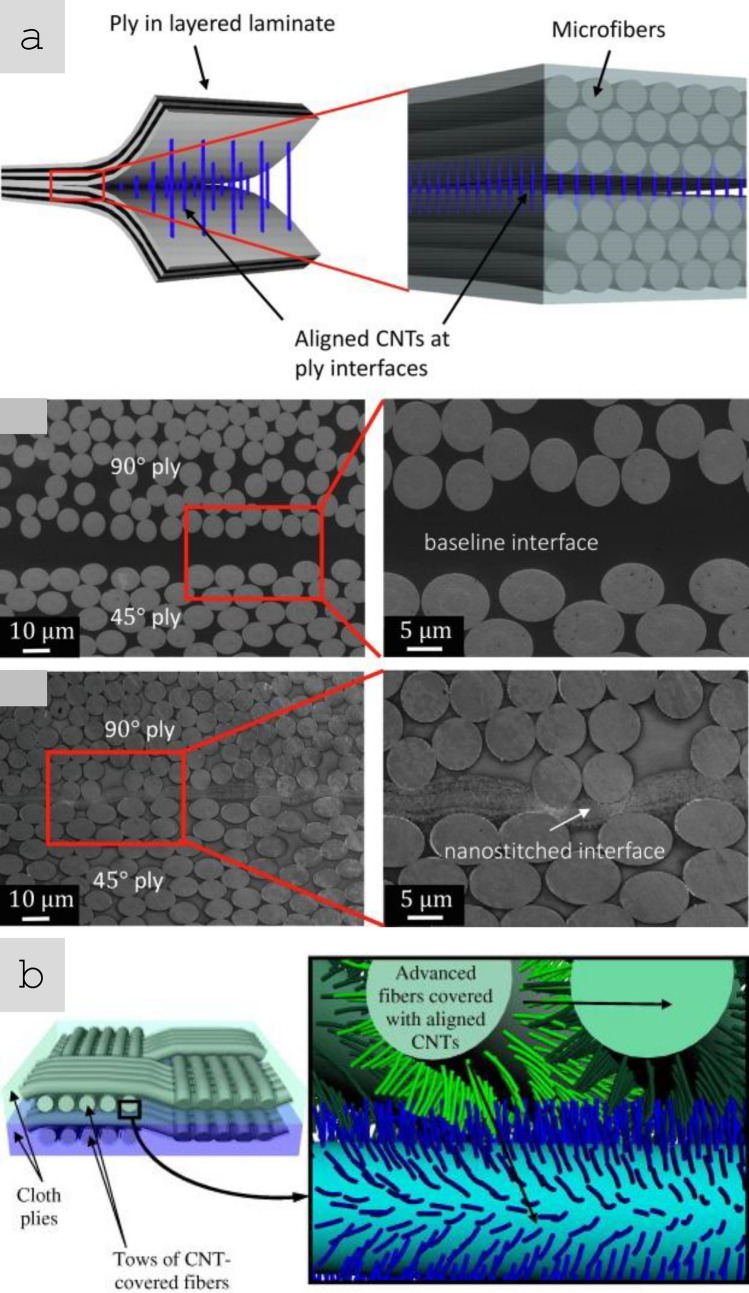
Strategies for interlaminar reinforcement with nanocarbons. **a** Prepegs with arrays of vertically-aligned short CNTs, termed ‘nanostitching’ [332]. **b** An example of a 3-dimensional CNT-reinforced composite: a ‘fuzzy-fibre’ composite with in situ-grown radially-aligned CNTs on the woven cloth [329]. **a** Reproduced from [332] with permission. Copyright 2016 Elsevier. **b** Reproduced from [329] with permission. Copyright 2008 Elsevier

So far, most work on structural materials with nanomaterials has been on combining fillers or sheets with CF to produce hybrid composites with improved interlaminar mechanical properties and/or higher electrical conductivity through the thickness (see discussion on edge-glow above). Interlaminar properties of aeronautical components are evaluated through fracture toughness in Mode I (G_*IC*_) and Mode II (G_*IIC*_), as well as compression after impact (CAI). They are measured on large panels, each typically 2 × 100 × 100 cm, comprised of multiple layers of CF fabric and produced using dedicated composite fabrication methods for incorporation/curing of the polymer matrix. Because of the requirement for relatively large amounts of nanomaterials, stringent fabrication methods, and component-specific properties, research on nano-augmented aeronautical composites has often been undertaken by large academic/industrial consortia. Selected examples are discussed below:

- Smart Intelligent Aircraft Structure (2011—2015) SARISTU project (FP7—EC) [328] involved developing methods to disperse CNTs, combine them with thermoplastics and integrate them into CFRPs (veils, powdered doped prepreg, and adhesives with fillers). Remarkable mechanical improvements of aeronautical composites were obtained at coupon level in CAI (up to 50% improvement with CNT treated/doped prepreg) and G_*IC*_ (close to 150% improvement with CNT doped thermoplastic veils in combination with infusion materials). However, these achievements have not yet been transferred and/or demonstrated at the large aircraft component level.
- Nano-Engineered Composite aerospace Structures (2006 -) NECST project, led by MIT, included major aircraft producers (Airbus, Boeing, Bombardier). NECST was focused on interlaminar reinforcement through the joining of laminate layers with ∼20 μm CNT oriented in through-thickness direction by two methods: inserting arrays of short vertically-aligned CNTs in CFRP lamina, termed nano-stitching or nanostiched laminates (Fig. 44a) [326], and direct growth of CNTs on carbon fibres (Fig. 44b) [329]. The results showed that under optimised integration, nanostitched laminates have both improved interlaminar fracture toughness (2.5—threefold enhancement of G_*IC*_ and G_*IIC*_) [326, 330] and 14–40% increase in in-plane strengths [331], demonstrating improvements in both inter and intra-laminar properties.

In further developments, some of these research groups have demonstrated additional improvements in interlaminar properties under short beam shear (SBS) fatigue tests: 115% fatigue life increase at 60–90% of static strength and a 249% in high-cycle at 60% of static strength [332]. For both routes, major manufacturing obstacles were overcome. The manufacturing of interleaves of vertically aligned CNTs has been scaled up and industrialised over the last decade [8]. Nanostitching integration in prepreg laminate by N12 has led to multifunctional laminate properties such as laminate strength, toughness, and electrical and thermal conductivities. Recent progress for direct growth on CF has demonstrated continuous CNT synthesis without degradation of the underlying CFs while improving interfacial shear strength to 101 MPa [333].

● EU Graphene Flagship—WP15 (Production). A consortium of research institutions and aeronautical companies have studied the addition of graphene to aerospace-grade resin matrices for the mechanical improvement of the horizontal tail plane leading edge of the A350. The results from testing obtained up to now show that graphene increases the mechanical properties of the leading edge in terms of impact resistance or CAI by around 10%. In related developments, the addition of graphene to CFRP has been reported to produce increases of CAI of up to 25%, interlaminar shear strength (up to 50%), G_*IC*_ and G_*IIC*_ (up to 40%) and impact energy absorption (up to 150%) [334, 335], as well as reductions of water uptake by epoxy resin of around 14%.

There are many other methods and examples of interlaminar reinforcement with nanocarbons, using spray methods [336], non-woven fabrics [337], etc*.* Furthermore, following developments using nanocarbons, several other nanostructured materials have been explored for interlaminar reinforcement, most notably electrospun polymer felts, nanocellulose and nanoaramid. A review of all these materials is beyond the scope of this work; however, in Table 15, we provide selected examples that show the magnitude of improvements observed in aeronautical structural composites.

**Table 15:** Examples of mechanical improvements in laminated structural composites with nanomaterials

Nanomaterial type	Lamina type and fabrication method	Nanomaterial mass fraction/areal density	Mechanical improvement
Filler: CNT in epoxy	UDCF –Autoclave curing	1% wt	60% G_*IC*_ [338], 75% G_*IIC*_
		0.5% wt	83% G_*IC*_ [339]
Filler: CNT in epoxy	UDCF—VARTM	0.5, 1 and 1.5% wt	25%, 20%, 17% G_*IC*_ [340]
CNT grown on CF	Woven CF—VARTM	2.24% wt	83% G_*IC*_ [341]
CNT sheet by spray coating	UDCF—VARTM	0.6% wt	24% G_*IC*_ [342]
	UDCF—Hot press	1.2 g/m^2^	46% G_*IC*_ [343]
Interleaved CNT sheets	UDCF- hand lay-up	0.2 g/m^2^	13% G_*IC*_ [344]
	Woven CF-VARTM	0.8 g/m^2^	60% G_*IC*_ [337]
	UDCF—Hot press	0.8 g/m^2^	88% G_*IIC*_ [345]
Graphene 1 (GNP or GO, as filler)	Three-roll-mill process, UDCF Prepreg and lay-up (16 plies)	0.5% wt	Up to 51% G_*IC*_ [346]
Graphene 2 (GO, as inter-laminar reinforcement)	GO dispersion in PVP polymer carrier, applied in the CF composite interlaminar region	0.007% wt	100% G_*IC*_ [347]
Graphene 3 (as sheets)	Electrospraying for selective deposition of graphene sheets on the surface of carbon fabrics		50% G_*IC*_
Nanoaramid (as inter-laminar reinforcement)	Nanofibres deposited onto the surface of woven carbon fabric using a spray-coating technique		33.7% and 81.6% [348]
Interleaved polymer veils	UD-GF- HVARTM	12–22 g/m^2^	90% G_*IC*_, 100% G_*IIC*_ [349]
	Woven GF- Hot press	3 g/m^2^	50% G_*IC*_ [350]

#### Reinforcement fibres

**Fig. 45:**
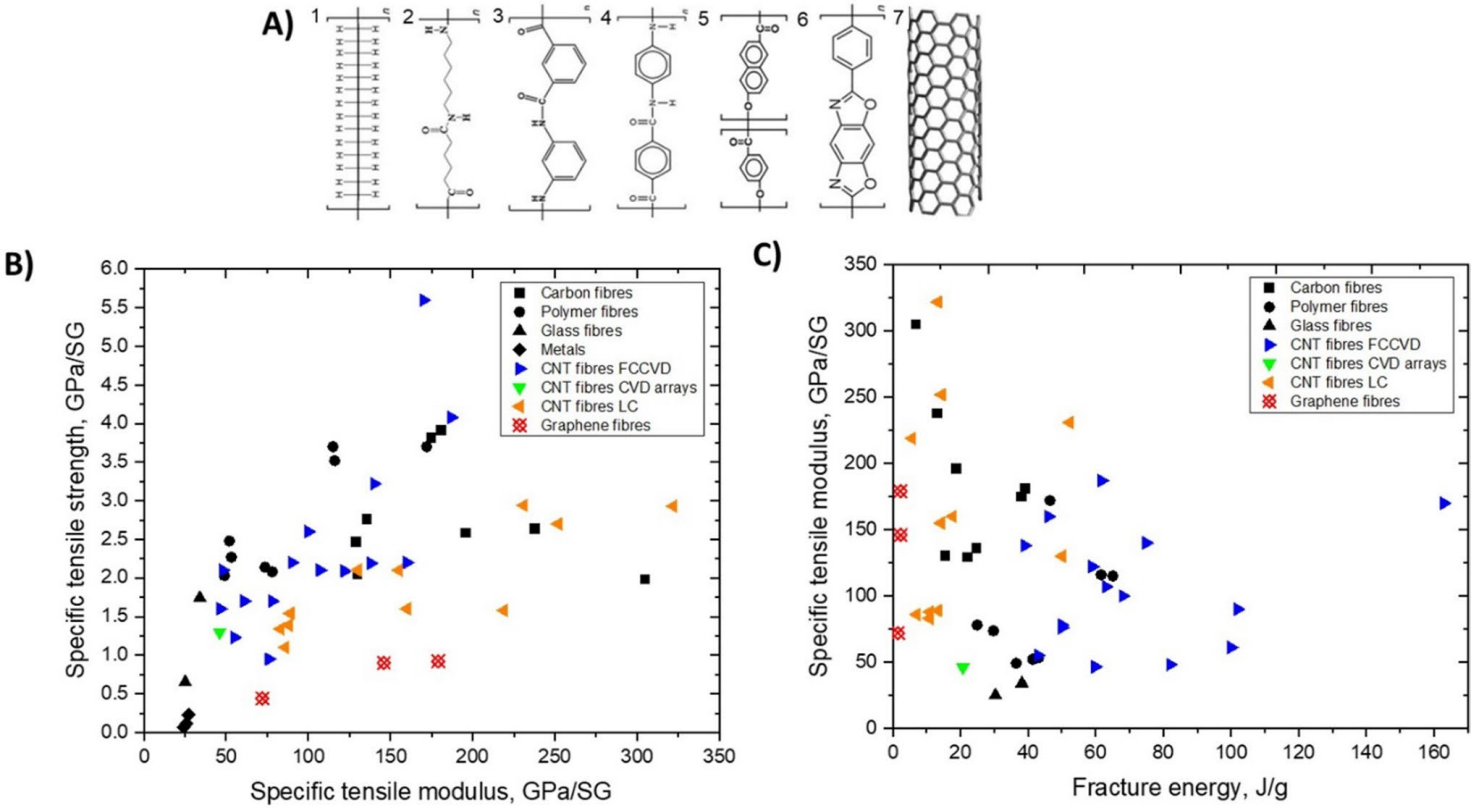
Properties of fibres of nanocarbons. **a** Analogy to polymers used for high-performance fibres: 1—polyethylene; 2—nylon 6; 3—meta-aramid (Nomex); 4—para-aramid (Kevlar); 5—LC aromatic polyester (Vectran); 6—PBO (Zylon); 7—a single-walled CNT as the “ultimate” polymer. **b,c** Tensile properties of CNT and graphene fibres compared to other materials, including high-performance CF (T300, T1100GC, M35JB, M60JB, AS4, AS7, IM10, HM63), polymer fibres (Techora, Vectran-HT, Vectran-UM, Kevlar 29, Kevlar 49, Dyneema SK75, Zylon), glass fibres (S-glass, R-glass), and metals (Ti–6Al–4V, SS316, Al6061). Copyright 2024 IMDEA Materials

Aligned fibres are a natural architecture for high-aspect-ratio nanomaterials, following the basic principles to make high-performance fibres based on extended polymer chains previously applied to produce all the synthetic polymer fibres currently used in aircraft (aramid, PBO, CF, etc*.*). Figure 45 presents a summary of the specific strength and specific modulus of fibres of CNTs, which are the most technologically mature of nanostructured fibres under development. Their tensile properties are currently on par with some CFs and doubling every two years [111, 134], a pace of development ten times faster than for CF [68]. Very importantly, in nanocarbon-based fibres, the fabrication of building blocks and their assembly are separate stages, similar to polymeric fibres but in contrast with CF. As a consequence, nanocarbon-based materials give access to mechanical properties beyond the property envelope of synthetic fibres used in aviation. As an example, Fig. 45c shows the exceptional fracture energy of CNT fibres, in some cases above UHMPE or PBO fibres in terms of strain wave velocity for ballistic performance [351], auguring enormous potential against impact.

#### Mechanical properties of CNT fibre-reinforced composites

The most significant progress in the area of composite laminates has been achieved using ultra-high volume fractions of high-performance CNT yarns through the NASA research initiatives in the following topics: i) prepregs, ii) tensile properties of unidirectional, cross-ply, and quasi-isotropic CNT yarn-based composites, iii) analysis of their transversal and other properties. CNT rovings and epoxy-infiltrated prepregs with improved tenacity and shear properties were developed [352]. A sufficient degree of resin infiltration was achieved by using current-induced swelling and wet-stretching with optional calendaring. The resin infiltration was assessed using pull-out tests, and the resultant CNT-yarn composite fibres demonstrated an apparent interfacial shear strength IFSS of 17.50 MPa.

**Fig. 46:**
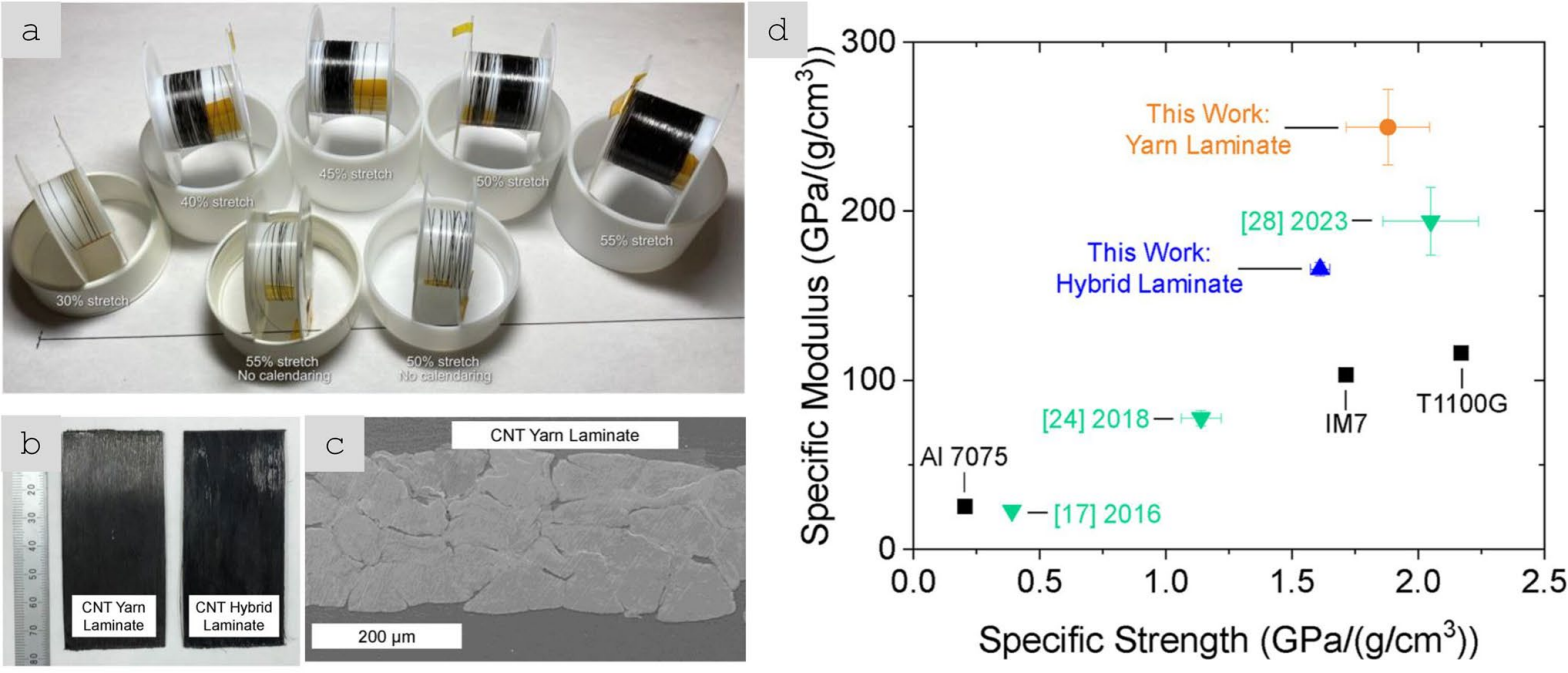
**a** CNT prepreg spools, **b,c** CNT yarn laminates and **a** SEM cross-section showing densely-packed CNT yarns; **d** specific tensile modulus and strength unidirectional CNT yarn laminates compared to the state-of-the-art high-modulus and high-strength CF composites. **a** Adapted from [352] with permission. Copyright 2024 Elsevier. **b-d** Adapted from [141] with permission. Copyright 2024 Elsevier

Figure 46 illustrates the state-of-the-art of unidirectional laminates reinforced with CNT yarns. The composites exhibit a specific tensile modulus comparable with or superior to that of CFRP reinforced with M60J high-modulus carbon fibres and nearly double the moduli of IM7- and T1100G-reinforced CFRP used in aerospace. The achieved specific tensile strength and modulus are close to 2 GPa/(g/cm^3^) and  GPa/(g/cm^3^), comparable to IM7 composites and superior to M60J composites, demonstrating an attractive combination of mechanical properties for structural applications [141, 353].

Transverse tensile properties and fracture toughness of unidirectional CNT fibre composites were investigated [354]. Owing to the improved load transfer in CNT composites, transverse tensile strength (TTS) was as high as 81.3 MPa/(g/cm^3^), which is higher than literature values for CF/BMI composites (42.0 MPa/(g/cm^3^)). The toughness mechanism with multi-scale fibre and CNT bundle bridging contributed to improved Mode I fracture toughness (G_*IC*_ of 0.670 kJ/m^2^).

Most recently, unidirectional, cross-ply, and quasi-isotropic composite laminates were made with high volume fractions of CNT yarns and bismaleimide (BMI) resin [355]. The work is the first-of-this-kind attempt to translate the highly anisotropic properties of CNT yarns into multi-directional laminates. The achieved properties were at the level of composites reinforced with IM7 thin-ply prepreg (70 gsm). The challenges of the CNT-yarn reinforced composites for structural applications are mainly related to the material availability. There has been a notable progress in the attainable size of the specimens over the last decade, starting from few-mm width laminates made with laboratory-produced CNT fibres [356] to intermediate-scale coupons and wider double cantilever beam panels reinforced with commercially produced CNT yarns [354]. Yet, testing procedures adaptation remains a practical challenge at the current state of CNT yarns’ production maturity, and the comparison with traditional CFRP at the larger-scale levels (close to established ASTM standards) will require kilometers of yarns as an expected outcome of the current scale up processes.

#### Application in current and future aircraft

**Fig. 47:**
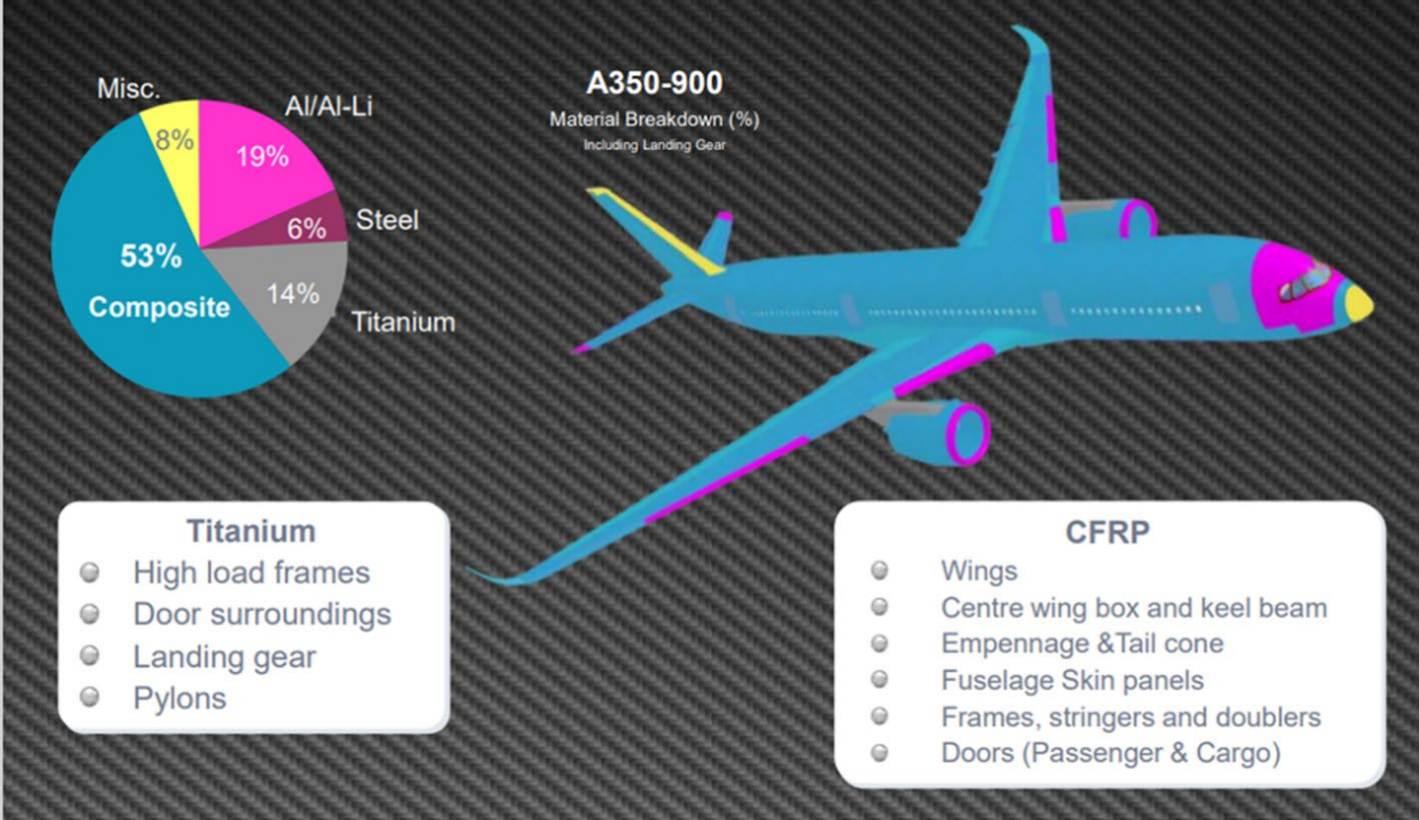
Material breakdown in last generation A350 aircraft. Copyright 2024 Airbus

CFPR are already highly optimised structures, hence, the room for improvement of mechanical properties of the next generation aircraft is small. However, reducing the environmental footprint of aviation for the next 10—15 years requires solutions applied to today’s aircraft. Weight reduction may be the key element in this endeavour. Given that the last generation aircraft (e.g., A350 or Boeing 787) has more than 50% of structural weight in composite materials (Fig. 47), reducing structural weight for composites through the integration of nanomaterials offers one of the quickest options for high-impact sustainability.

For upcoming hybrid/electric aircraft, an additional opportunity for nano-augmented structural composites is Hydrogen tanks. Depending on the design, Hydrogen can be stored as a gas or a liquid. As a gas, it typically requires high-pressure tanks (350–700 bar), while as a liquid, it requires sustaining cryogenic temperatures of around − 250 ºC. Liquid hydrogen is the preferred option in civil aviation. Based on data from space cryogenic tanks, composite tanks are expected to be ~ 25% lighter than metallic analogues. Although there have been several attempts to implement composite Liquid H_2_ for space application, e.g., by NASA, there are still key challenges that need to be overcome and demonstrated, which are increasingly difficult for aircraft application due to its operation conditions:

- Geometry/integrity and associated manufacturing challenges:
  - Cylindrical vs. complex shapes (improved volumetric efficiency)
  - Load-bearing tank versus only hydrogen container
- Temperature insulation & thermal cycling:
  - Insulation technology to maintain a T < − 252ºC to minimise boil-off
  - Thermal expansion/contraction during tank filling/draining
  - CTE differences between materials (e.g., carbon fibre and resin)
  - Micro-cracking in the case of composites
- Hydrogen permeation: critical for composite tanks due to micro-cracking

**Fig. 48:**
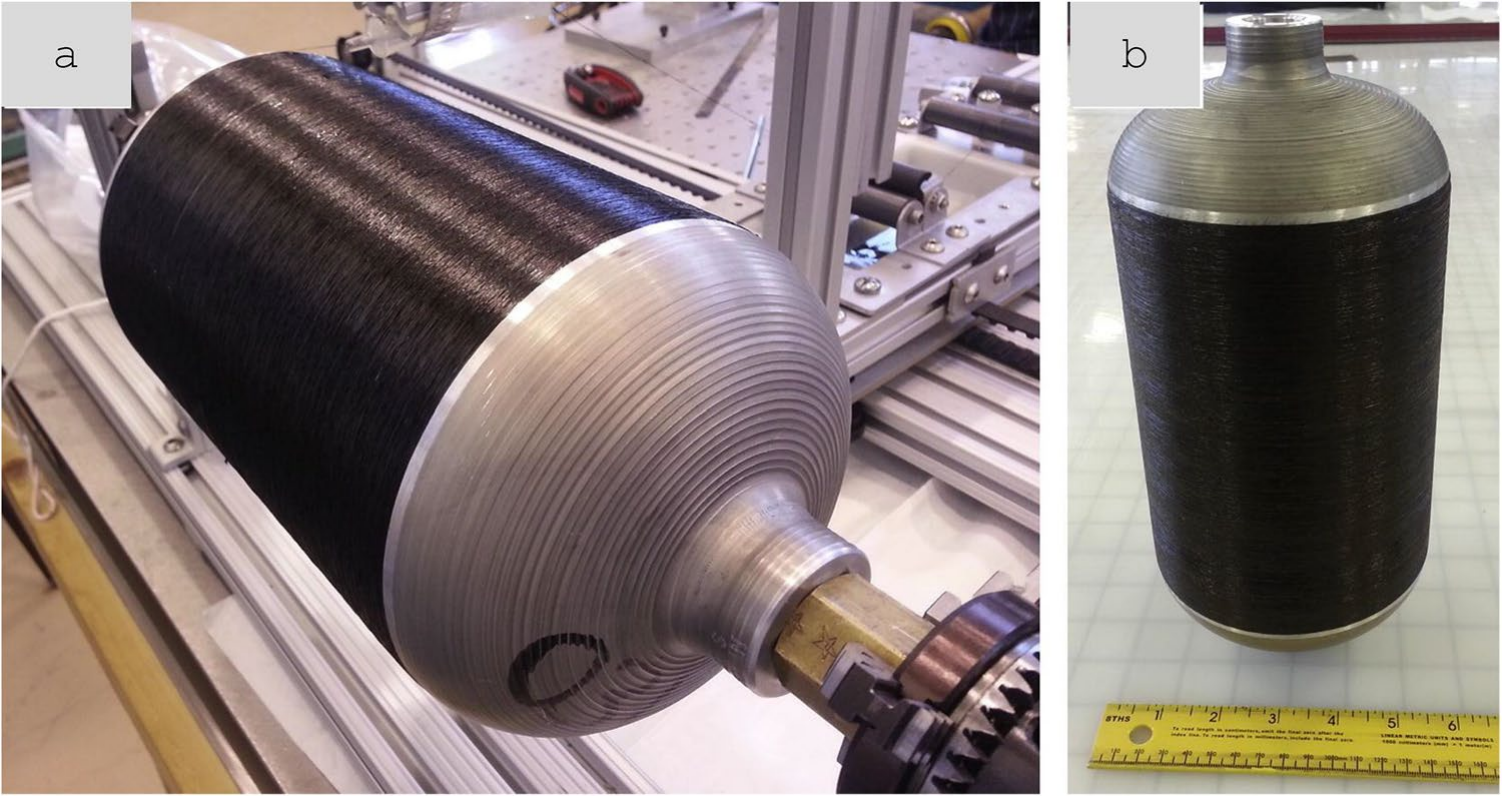
**a** CNT yarn-composite overwrapped pressure vessel for aerospace vehicles [358]. Adapted from [358] with permission. Copyright 2016 Elsevier

Of the several possible contributions of nanomaterials to this list of properties, the most promising is the improvement of polymer matrices to reduce matrix cracking and reduce H_2_ diffusion in linerless composite cryo tanks. Infinite Composites has successfully incorporated AGM’s (Applied Graphene Materials) graphene technology into two resin systems for cryogenic pressure vessels, which are being considered for use in multiple NASA spaceflight missions. The addition of graphene technology has enabled the tanks to complete, in 2019, their first liquid oxygen loading test at − 300ºF pressurized to 600 psi [357]. Analysis of the composite structure showed that the addition of graphene eliminated nearly all micro-fractures in resin samples after exposure to the extreme cryogenic environment versus the control samples. These results highlight the interest in using nanomaterials to increase fracture toughness and reduce micro-cracking of composite materials in cryogenic conditions to ensure long-term durability. On the other hand, NASA explores the usage of carbon nanotube-based materials as reinforcement in Composite Overwrapped Pressure Vessels (COVPs) for a range of potential applications in aerospace vehicles [358] in order to increase the strength-to-weight ratio and system performance. For this, a continuous stretcher and winder apparatus were developed to produce the CNT yarn/tape composite wrapped aluminum rings and a pressure vessel completely wound with a CNT yarn composite in the hoop direction (Fig. 48).

## Thermal management

### Overview of thermal properties of nanostructured materials

Much thermal management in transport components is done at the systems level; however, the materials within them play a key role in heat management performance [359]. In the case of nanostructure materials, applications may be divided into those that are individualized in a fluid, termed nanofluids, or as solid ensembles.

For nanofluids, the rationale is that nanoparticles can improve heat transfer because of a) the large SSA of nanoparticles and b) higher colloidal stability due to reduced sedimentation, amongst other factors [360]. However, the role of particle dynamics in the fluid and the formation of a large nano-scale interface with the fluid lead to interrelated effects of particle size and volume fraction and imply that the design and analysis of nanofluids is complex and difficult to reduce to simple properties of the nanoparticles and the base fluid [361]. A literature review shows improvements in thermal conductivity relative to the base fluid peaking at around 30%, irrespective of particle chemistry or shape [362]. An example of different nanofluids consisting of water and various nanoparticles is shown in Table 16. Relatively high thermal conductivity (2.39 W/mK) has been achieved by using boron nitride nanosheets, however, at a very high volume fraction (24 vol%) that thus leads to viscosity exceeding 1000 mPa⋅s [363].This suggests that there are fundamental limits on absolute conductivities below a few W/mK using nanofluids.

**Table 16:** Comparison of various thermally conductive materials for nanofluids [363]

Particle	Concentration (vol%)	Thermal conductivity (W/mK)
CuNW	0.25	0.854
BNNT	6.0	1.54
CNTs-Al_2_O_3_	1.0	0.696
MWCNTs	1.0	0.78
TiO_2_	1.0	0.648
SiO_2_	8.0	0.654
Fe_2_O_3_	5.03	0.675
BNNT nanosheets	24.0	2.39

**Fig. 49:**
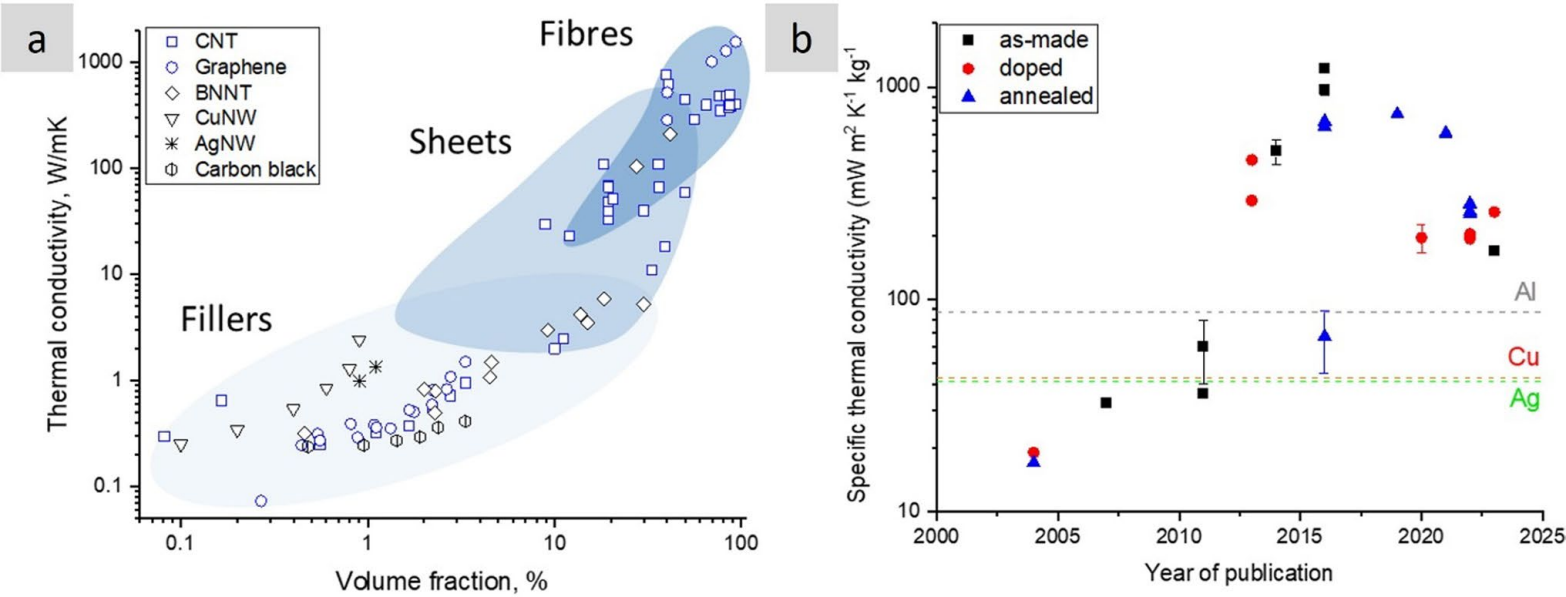
Thermal conductivity of macroscopic nanomaterials. **a** Thermal conductivity for different nanobuilding blocks organised as randomly oriented fillers, sheets or aligned fibres. **b** Progression on fibres of aligned CNTs and graphene at the high end of specific thermal conductivity. Copyright 2024 IMDEA Materials

Alternative forms of thermal management relevant to transport applications use high thermal conductivity solids. As observed for electrical properties, there are distinct property regimes based on the organisation of nanobuilding blocks. For nanocomposites with randomly distributed high-aspect ratio nanoparticles and polymer matrices, thermal conductivity peaks at a few W/mk (typically, under 10 W/mK; Fig. 49a). Nanoparticles organised in sheets with volume fractions of up to 40% provide values of 1–100 W/mK with a few exemptions of higher thermal conductivity observed for BNNT- [364] and graphene-based [365] sheets. Aligned fibres, so far only produced from nanocarbons, routinely lead to thermal conductivities above 400 W/mK, and as high 970 W/mK achieved for as-produced FCCVD CNT fibres [133], 1290 W/mK for graphene fibres annealed at 2850 °C [138], and 1575 W/mK for well-packed graphene sheets annealed at 2500 °C. [366]. To put the values at the high end into perspective, in Fig. 49b, we present the progression of specific thermal conductivity of aligned nanocarbon fibres over years of development. The data show several examples above the mark for metals.

### Thermal management systems in aviation

**Fig. 50:**
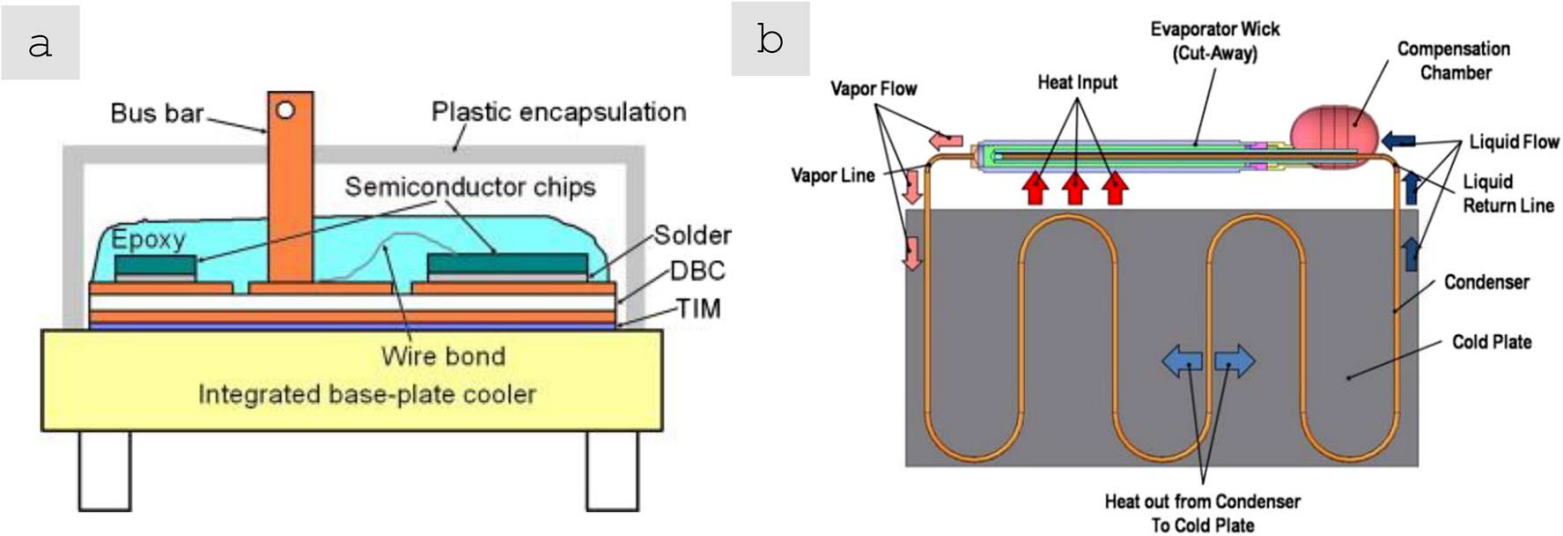
Examples of thermal management systems relevant for aircraft. **a** Thermal interface material and the integrated base-plate cooler in a typical power electronics package [367]. **b** Loop Heat Pipe. **a** Reprinted from [367] with permission. Copyright 2013 Elsevier. **b** Reprinted from [368] with permission. Copyright 2009 ASME

Common examples of solid materials used for thermal management are thermal interface materials (TIMs) and loop heat pipes (LHPs) (Fig. 50). TIMs consist of solids that transfer heat from a source, such as an electronic device and a heat sink. They may be thin layers, viscous filler-containing polymers, tapes, or another form of solids (Fig. 50a). Although the interfacial thermal resistance in their embodiment is of importance, as a key property for analysis, we may take specific thermal conductivity. Other solid materials used for thermal management may also be considered, such as phase-changing materials combined with an embedded conducting material such as graphite.

LHPs are used for the passive removal of heat from a source by capillary action (Fig. 50b). They are generally made of Ni or stainless steel and have the dual role of driving the capillary action, promoting the operating fluid’s phase change, and transferring heat to the fluid.

LHPs and general heat exchangers benefit from high thermal conductivity and high SSA. As mentioned earlier, an aspect that sets nanomaterials apart is the possibility of producing macroscopic solids in which porosity is attained through the “imperfect” assembly of nanobuilding blocks, but without necessarily introducing defects into the constituents. Effectively, such a bottom-up approach leads to combinations of SSA and thermal conductivity that are not attainable with traditional top-down processing of metals.

All materials and systems involved in thermal management in medium aircraft are grouped into what is termed the Aircraft Thermal Management System (TMS). It includes heat removal to protect equipment, systems and structures, and maintain passenger thermal comfort [369]. In conventional turbojet and turboprop-powered aircraft, the TMS essentially consists of a network of ducts, valves and regulators to conduct medium to high-pressure hot air, "bled" from the compressor section of the engine(s) and APU, to various locations within the aircraft [370]. The TMS utilises engine fluids to transfer excess heat from the engine heat sinks to heat exchangers. Taking such a system as a starting point, the first opportunity for improvements in heat transfer is the use of a nanofluid and a nanostructured heat exchanger.

However, the next generation of more electric aircraft requires an altogether different TMS. This is not only because current thermal management systems are already working to their limits but also because using more electrical motors requires TMS to be “bleedless”. LHPs have been widely researched in aerospace [371] and will gain further adoption. They do not need any mechanical parts and require less maintenance than traditional pump-based thermal control systems. Hence, they are already used as cooling devices for electronic components in space vehicles and satellites (and expected to keep steady growth at a CAGR of 9% [372]).

**Fig. 51:**
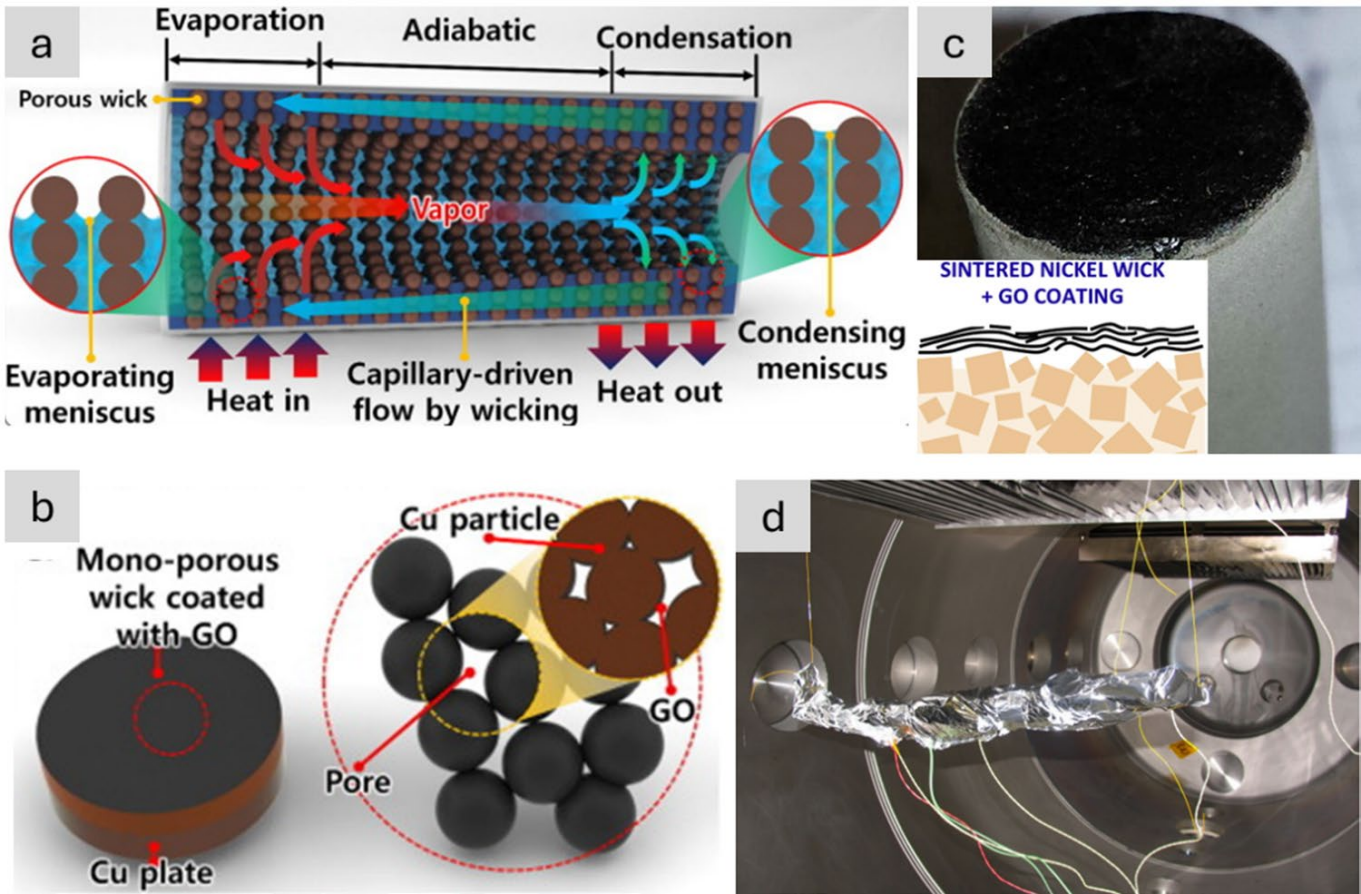
**a** Schematic of the two-phase flow loop in heat pipes, **b** Porous copper wick with a nano-layer of GO [375], **c** nickel wick coupon coated with graphene slurries [376], **d** graphene-coated loop heat tested in a vacuum chamber[373]. **a,b** Adapted from [375] with permission. Copyright 2018 Elsevier. **c** Adapted from [376] with permission. Copyright 2016 Elsevier. **d** Reprinted from [373] under the Texas Tech University Repository Submission License

LHP with graphene-coated wicks were recently subjected to zero gravity conditions and tested on-ground in a space simulator (Fig. 51). The results showed that graphene improved performance with respect to the wicking capacity and vapour production [373]. The authors also noted enhanced reliability of the full LHPs [374]. These results under operational conditions demonstrate the rapid implementation of nanomaterials in thermal management [375–378].

To summarise potential improvements at the materials level, in Table 17, we compare basic properties for thermal management of currently used materials and nanomaterials. We identify specific thermal conductivity and specific surface area as entry performance indicators. Nanofluids are a promising avenue for improved thermal management. However, the benefits of the addition of nanomaterials will depend strongly on the system parameters of the cooling systems and are, thus, not included in the Table. On the other hand, the combination of high SSA and thermal conductivity of nanostructured networks for solid elements of heat exchangers is inherently above metallic meshes and CF. Indeed, CF are increasingly used for power electronic devices in electric vehicles and as TIM in portable electronics (e.g., Samsung Galaxy Note 9 [379]) as a lighter alternative to metals. Nanocarbon ensembles with higher thermal conductivity and larger SSA could be quick replacements. We note, though, that whereas a high SSA is beneficial for heat transfer, fluid flow is dependent on permeability and thus pore size distribution, highlighting that other materials’ properties are relevant for a full assessment of heat transfer systems and that harnessing the full potential of ultra porous thermal conductors may require changes in heat exchange design.

**Table 17:** Thermal conductivity and SSA of nanostructured solids

Material	Specific thermal conductivity W/mK/g/cm^3^	Specific surface area (m^2^/g)
CNT sheet (random, dispersion of CNTs, 5–15 µm length)	0.26–0.40 [380]	~ 250
CNT sheet (aligned, MWCNT forest, 300 µm length)	83 [381]	
BNNT sheet	102 [364]	N/A
CNT fibre	1220 [133]	~ 250 [382]
CF K1100^a^	611	< 0.2
CF K13D2U^b^	367	
Commercial graphite sheet TIM [383]	642	< 0.01

^a^Amoco 1998

^b^Pitch-based, Mitsubishi Chemical Carbon Fiber and Composites, Inc

Finally, we note that in the future, electric/hybrid aircraft thermal management systems will also have to address heat supply. A possible location for electrochemical energy storing systems is non-pressurized areas, such as the rear-end fuselage area, where the APU (Auxiliary Power Unit) is placed. This area can reach − 55 ºC, which is far beyond the operation limits of conventional electrolytes. Even back cargo compartments can routinely be at 5 ºC. Energy storage systems will likely require a substantial heat supply to operate, thus highlighting the importance of thermal management materials in future aircraft.

## Other areas of development

There are multiple other envisaged applications of nanomaterials in critical components of future aircraft. Some are more indirect applications of nanomaterials, and others are related to emerging systems that are still under constant changes. For brevity, only a brief summary of selected topics is provided below.

### Low CO_2_ footprint materials

**Fig. 52:**
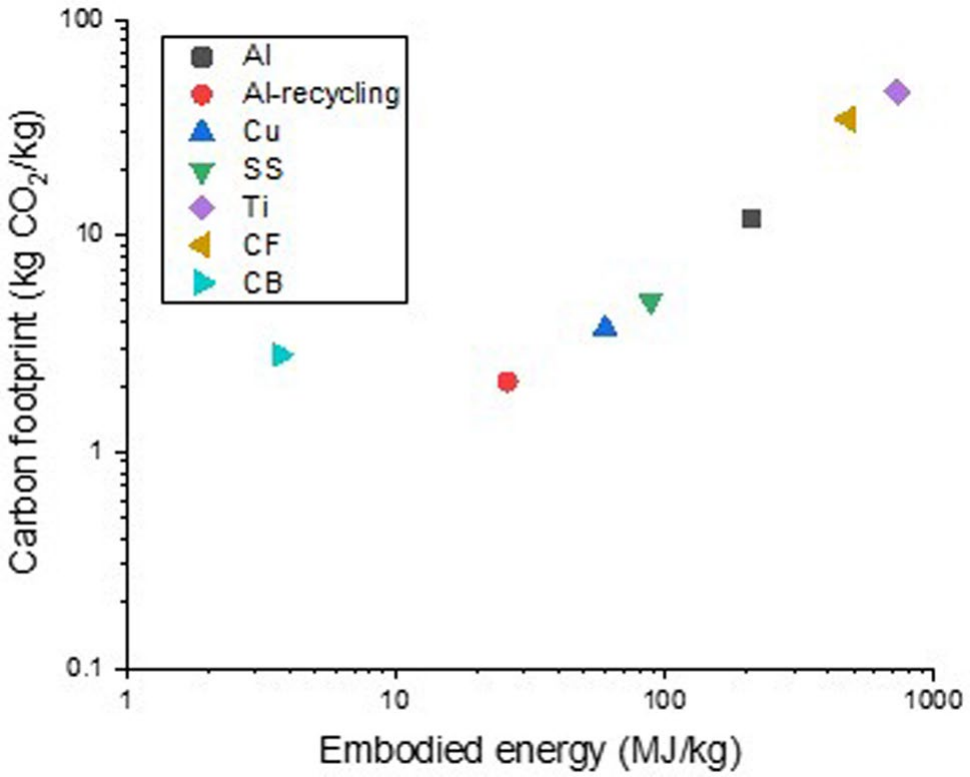
Approximate carbon footprint of selected aviation materials. Sources: Cu, Ti, Al, CF [384], CB [385, 386]. Copyright 2024 IMDEA Materials

As defined in Sect. 2.2, most aviation CO_2_ footprint stems from using kerosene fuel during operation, with a minor contribution from aircraft manufacture and production of raw materials. However, as progress is made towards carbon–neutral emissions aircraft by 2035, the focus will shift to reducing embodied emissions. The dominant materials in today’s aircraft are CF, Ti, Al, and Cu. Of these, both CFRPs and Ti have increased in last generation aircraft (e.g., A350 and/or Boeing 787) and constitute about 66% of the weight of aircraft structure. A rough comparison of the carbon footprint of these materials shows that CFRPs and Ti are about a factor of 5—10 more intensive than other metals, with around ~ 30 kg and 40 kg CO_2_/kg, respectively (Fig. 52) [384].

Several large-scale initiatives pursue the reduction of emissions in metallurgical processes and will contribute to reducing embodied CO_2_ in aircraft structures. With respect to nanomaterials, two avenues hold promise. The first is the direct conversion of CO_2_ into nanocarbons. Examples of promising routes are the thermocatalytic conversion of CO_2_ into graphene [387], molten electrolysis [388] and revalorisation of waste in metallurgical processes to produce CNTs [389]. The second avenue is the synthesis of nanocarbons as by-products in the production of Hydrogen from natural gas, pioneered by the Carbon Hub [390], an international initiative in large-scale deployment with support for leading companies in the transport, energy and wiring sector. This strategy relies on the thermocatalytic decomposition of natural gas (mainly CH_4_) into H_2_ fuel and high-performance C nanomaterials [385]. Conceptually, the vision is that both the fuel (H_2_) and materials (structural applications and conductors) could be produced from the same low CO_2_ intensity process. Naturally, since aircraft uses several times their weight in fuel per year, emissions would need to be further offset by the replacement of metals in other industrial sectors. The key to these developments is to increase the efficiency of CVD processes. Preliminary studies suggest embodied emissions of 20 kg CO_2_/kg for fluidised bed CVD after scale up [391], below some CF. More recent studies have analysed the effect of carbon sources and sources of electrical power [392]. They suggest possible avenues for embodied emissions approaching those of CB (< 3 CO_2_/kg) [385, 386], a solid form of C also produced by the thermal decomposition of hydrocarbons such as natural gas.

### Ceramic materials

Ceramic materials offer attractive properties, such as high-temperature stability, high hardness, and high corrosion resistance, suitable for high-temperature applications in aircraft [393]. Carbon nanotubes, graphene [394, 395], and graphene nanoplatelets [396] can be used to improve the fracture toughness of ceramic matrix composites. Anti-corrosion aviation coatings are developed using a wide range of polymeric systems (acrylic and epoxy resins [397], polyetherimide [398], fluorine-based [399], etc*.*), with addition of nanocarbon (fullerenes, carbon black, carbon nanotubes, graphene, graphene oxide, and carbon dots) [400] or ceramic nanoparticles [401].

The polymer-derived ceramic (PDC) is an advanced approach to control the structure of ceramic fibres [402, 403], coatings, and composites at the molecular level [404]. PDC ceramic coatings can be manufactured on large complex surfaces at energy-efficient pyrolysis temperatures as low as 800 °C [405]. Ceramic fibres and MEMS devices have shown the outstanding thermal stability and are used in high-temperature stress, pressure, and temperature sensors in harsh conditions [406–408].

### Nanocellulose materials

Cellulose is a linear biopolymer found naturally in plant cells such as wood and cotton. By mechanical or chemical treatment, cellulose fibres can be converted into nanocelluloses (NC) or cellulose nanofibres (CNFs). A potential application of nanocellulose is as a precursor to obtaining carbon fibres in combination with SoA carbon fibre precursor PAN (polyacrylonitrile). Nanocellulose whiskers have been used to obtain PAN/NC hybrid precursor, which effectively increases the crystallite size and tensile properties of carbon fibres [409]. These bio‐based precursors offer the advantage of using renewable resources to produce carbon fibres; however, the resulting fibres currently have very inferior mechanical properties compared to CF.

### Advanced composite part manufacturing

**Fig. 53:**
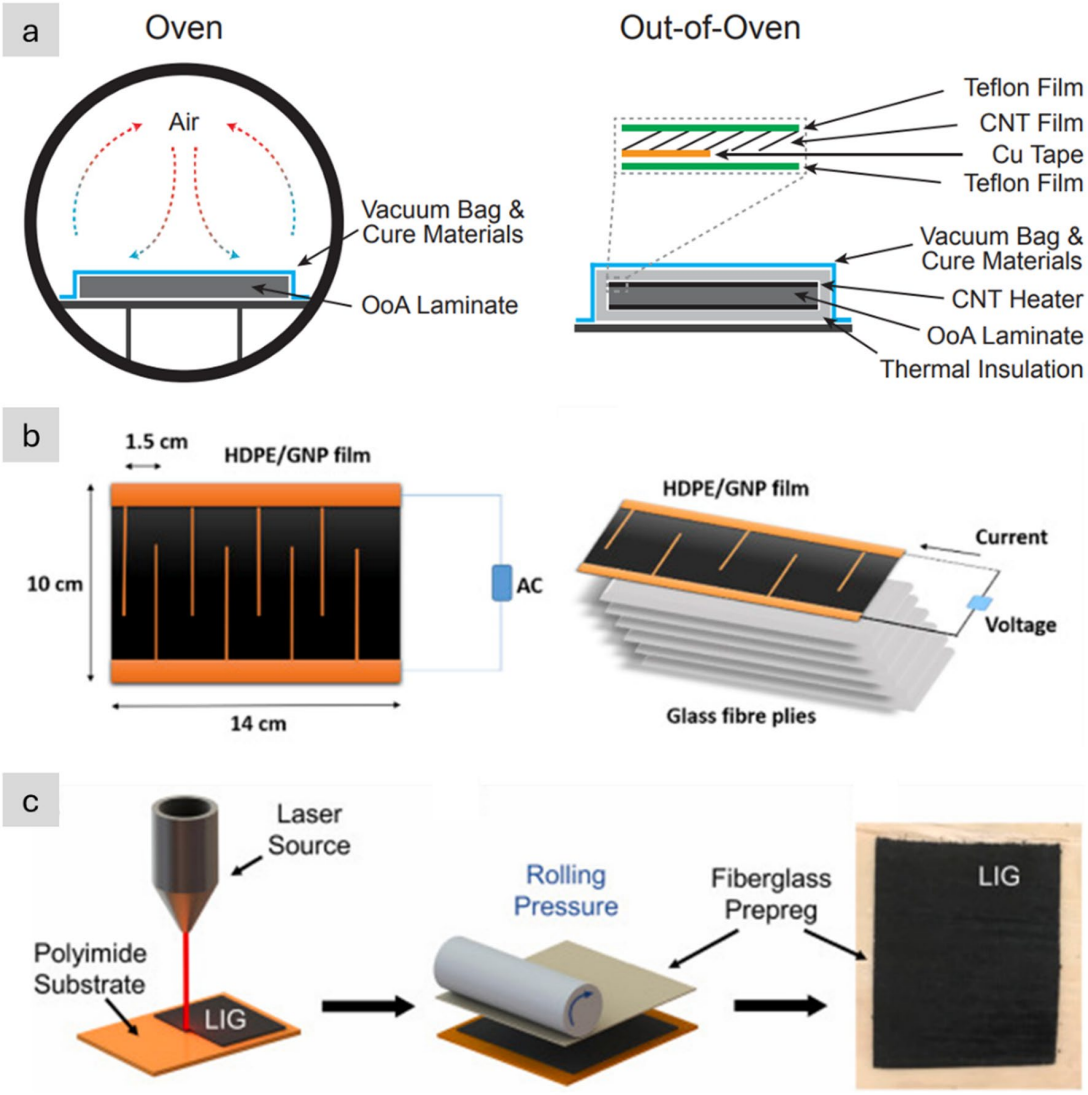
**a** Comparison of oven and out-of-oven manufacturing process for laminate curing [412]; **b** HDPE/GNP heating film for out-of-oven curing of glass fibre reinforced thermoset laminate [411]; **c** manufacturing of laser-induced graphene heating elements [174]. **a** Adapted from [412] with permission. Copyright 2018 Elsevier. **b** Adapted from [411] with permission. Copyright 2020 Elsevier. **c** Reprinted from [174] with permission. Copyright 2022 Elsevier

Next-generation composite component manufacturing processes are needed to overcome several limitations of conventional manufacturing techniques, most pressingly their high energy consumption. The use of nanomaterials in this field can also lead to several opportunities in terms of energy and cost saving, particularly in out-of-autoclave thermoset curing, bonding, and/or thermoplastic consolidation. Among these developments, one of the most advanced is the out-of-autoclave laminate manufacture via nanostructured out-of-oven (OoO) resistive curing [410]. A resistive heating element based on a thin CNT film (25 g/m^2^) can be directly integrated into the surface of a laminate so that curing does not require any heating vessel or convective medium (Fig. 53). In another example, nanocomposite heating film with intrinsic self-regulating heating capabilities was fabricated with polyethylene and graphene nanoplatelets of 24%wt loading for OoO curing of a glass fibre reinforced plastic [411]. Resistive elements for Joule heating curing and composite joints and repair can be made with laser-induced graphene layer. It is initially generated on polyimide substrates using CO_2_ laser engraving and then transfer-printed onto GF prepregs [174].

OoO curing enables highly efficient manufacturing of composites while preserving mechanical properties, particularly interlaminar strength, equivalent to the conventional oven method. When compared to oven curing of an aerospace-grade out-of-autoclave (OoA) carbon fibre prepreg advanced composite laminate, the OoO curing reduces energy consumption by over two orders of magnitude (14 vs. 0.1 MJ) [412]. In addition to energy savings, the OoO curing process can potentially reduce part-to-part variations through improved spatiotemporal temperature control. Most recently, OoO curing with carbon nanotube-based heaters and a polymer nanoporous network interlayer has proven the manufacturing of void-free autoclave-grade thermoset CFRP with a cure cycle accelerated by 35% compared to conventional autoclave fabrication [413].

**Fig. 54:**
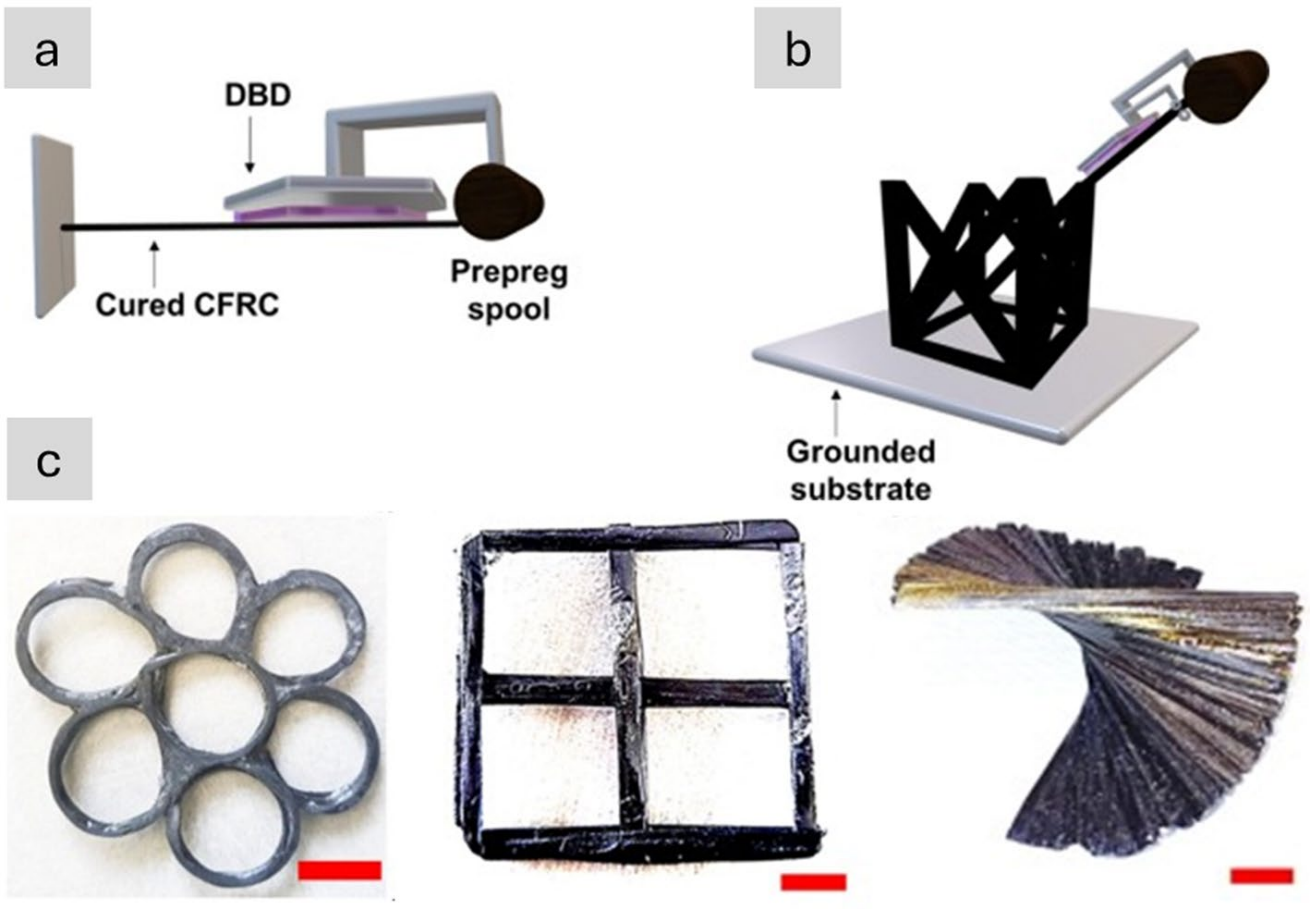
**a**, **b** The out-of-oven curing of CFRP composites using a dielectric barrier discharge applicator, **c** complex geometries of CF. **a-c** Adapted from [416] with permission. Copyright 2024 Elsevier

Out-of-oven heating can be achieved using electric fields generated by a radio frequency applicator, as has been demonstrated on CF prepregs [414]. Another novel approach of *in-situ* mold-less out-of-oven curing of continuous carbon fibre composites is developed using a Dielectric Barrier Discharge (DBD)-assisted Joule heating (Fig. 54), The DBD applicator ionizes the air, creating a plasma and allows on-the-fly printing and curing of CFRP in complex 2D and 3D geometries and multilayered structures [415]. The concept is also suitable for composite repair [416].

### Nanomaterials for structural health monitoring

Nondestructive characterisation (NDT) of the structural parts in aircraft (acoustic emission, ultrasonic, infrared thermography, shearography, Eddy Current testing, etc*.*) is crucial for safety and reliability of an aeroplane in operation [417]. The development of reliable NDT procedures and solutions for in-flight structural health monitoring (SHM) spans a multitude of aircraft applications, including the SHM of structural parts (fuselage frames, stringers, skin, wings ribs and spar, just to name a few), engine, and systems areas. The research efforts are primarily focused on novel sensor technology, system validation, and integration, but they must be supplemented with regulatory guidance, certification, and standardization of SHM solutions.

The optical technology and Optical Fibre Bragg Grating (FBG) are used for SHM [418] due to their high sensitivity and good electromagnetic interference resistance. They can be directly embedded into the CFRP laminates at discrete pre-defined locations (distributed sensing) and allow multiplexing [419]. Doping nanoparticles into the fibres’ core enhances the backscattered light, so the light signal for long distance sensing [420]. Yet, their known drawbacks, such as extreme fragility and the necessity of demodulator or interrogation systems to extract the measured information from the shift of Bragg wavelength, result in complexity and higher cost of specialized optical components.

**Fig. 55:**
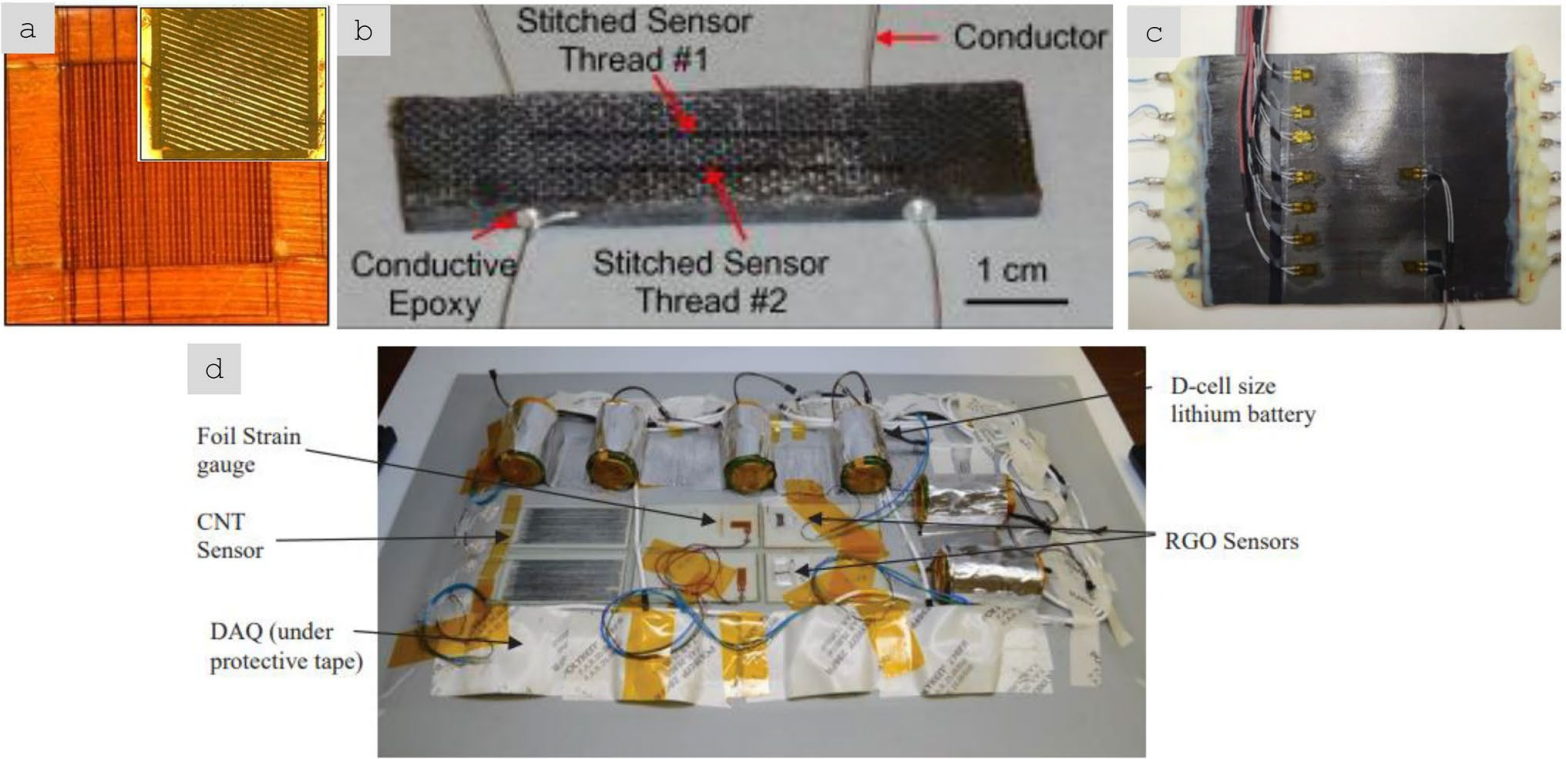
Examples of the piezoresistive strain sensors based on CNT yarns: **a** individually positioned in micro-channels in a polyimide film [423], **b** stitched to GF layup [424], **c** fully integrated in a CFRP panel as a sensor array tested for strain sensing and impact damage detection, **d** directly printed on the airframe part and subjected to real flight testing [425]. **a** Adapted from [423] under the Creative Commons CC-BY 4.0 license. **b** Adapted from [424] with permission. Copyright 2010 Elsevier. **c** Copyright 2024 IMDEA Materials. **d** Adapted from [425] with permission. Copyright 2019 AIP Publishing

Electrically-conductive carbon nanotube fibres and yarns exhibit piezoresistive behaviour with high sensitivity [421], and are used in various flexible devices and smart textiles [422]. Their key benefits, namely, high sensitivity to strain and stress, coupled with light density, superior flexibility and robustness, overcome the fragility and other limitations of FBG, as shown in Fig. 55. In one example, a strain gauge was developed with polyimide films, where piezoresistive CNT yarns were positioned by precision tweezers into micro-channels (grooves) created by a laser drilling technique [423]. Yet, owing to the superior fracture toughness, the CNT fibres and yarns can be bent through very tight radii without apparent permanent damage, so they can be easily introduced into fibre-reinforced laminates through various means: by stitching [424] or printing. Piezoresistive sensors based on continuous CNT yarns with polymeric coatings can be directly printed on airframe parts [425, 426] or fully embedded into CFRP as a continuous integrated array or network for strain sensing and damage detection.

The trend in smart sensing involves the development of the embedded sensors network with optimised sensor placement for real-time monitoring coupled with data processing by using machine learning and deep learning algorithms for automatic damage detection, localization, and classification [417]. As an example, DOMMINIO project^1^ develops continuous CNT yarns-based piezoresistive strain sensors suitable for integration into the structural CFRP laminates using the combination of robotized technologies, such as automated tape placement (ATL) and fused filament fabrication (FFF). The innovative methodology ensures cost-effective and sustainable manufacturing of multifunctional and intelligent airframe parts and SHM with data-driven fault detection. Simulation models and digital tools can be then implemented as a digital twin for a multiscale analysis based on the information gathered by sensors.

### Nanomaterials for interior safety and passengers’ health

The increasing use of composites and polymers in interior materials and non-structural parts and new fire risks in electric aircraft require a corresponding increase in the use of fire retardants. Multiple 1D and 2D nanomaterials have been effectively used as fire-retardant-fillers [427–430]. For their implementation in future aircraft, the main challenges are to ensure overall fire safety (heat release, smoke toxicity, etc.) at sufficiently low volume fractions to enable polymer processing and to preserve other properties of the host component. The novel graphene oxide-, semiconductor-, thermoelectric-based materials can be successfully applied to early-stage fire-warning systems for detecting fire situations in the pre-combustion processes [431]. The fire-warning films made of MXene/graphene can shorten the response time to 1 s with the ultrafast sensitivity at 250 °C [432] and combined with a luminosity sensor and signal transmission, as an example of the next-generation smart fire alarms.

Flight environment sensing, from temperature and humidity to pressure and health monitoring, can benefit from nanotechnology and novel materials. Silver, copper, and zinc nanoparticles hybridised with organic compounds are used in antimicrobial/antibacterial coatings and materials with antiviral efficacy [433]. Metal oxide nanoparticles introduced in air filters demonstrated antibacterial and antivirus effects with high reliability and safety [434]. Nanocarbon-based materials are studied for virus detection and protection measures in the post-COVID-19 era [435]. Air filters with filtration efficiencies up to 99.999% and self-sanitized capability by resistive heating were developed using ultra-thin electrically-conductive CNT mats with areal density of 0.1 g/m^2^, with proven deactivation of a betacoronavirus and an adeno-associated virus retained on their surfaces [436].

### Nanomaterials for magnets

Of the many materials science challenges specific to electric propulsion machines [437] one that stands out is the improvement of electric motors, generators, etc*.* This calls for the development and/or adoption of nanocrystalline permanent magnets [438, 439].

### Nanostructured superconductors

Superconducting properties can also benefit from nanostructuring. Compared to the bulk, nanowires of several metals (Pb, Sn) have shown an increase in critical field and/or the onset temperature of the superconducting transition [440, 441], two main figures of merit. This may reduce their cost, which can exceed €20,000/kg [143] due to high raw material and processing costs [442]. However, metallic nanowires have so far been assembled as microelectronic-scale arrays, mainly as transparent thin films and thin coatings for electromagnetic shielding or radio frequency communication in small devices [443].

### Thermal protection

There are additionally emerging materials for thermal protection in LIBs. One such example is porous ceramics, whose function is to act as insulation and a barrier in thermal runways. Given the brittle nature of monolithic ceramics, most solutions explored have a network structure, for example, foams, non-wovens or other fibrous embodiments. Nanostructuring network materials can increase the damage tolerance of the constituents, increase SSA and simultaneously increase porosity and contact between elements. These properties will likely result in improved processability and barrier properties after nanostructuring.

## Perspective for implementation of nanomaterials, engineering gaps and conclusions

**Fig. 56:**
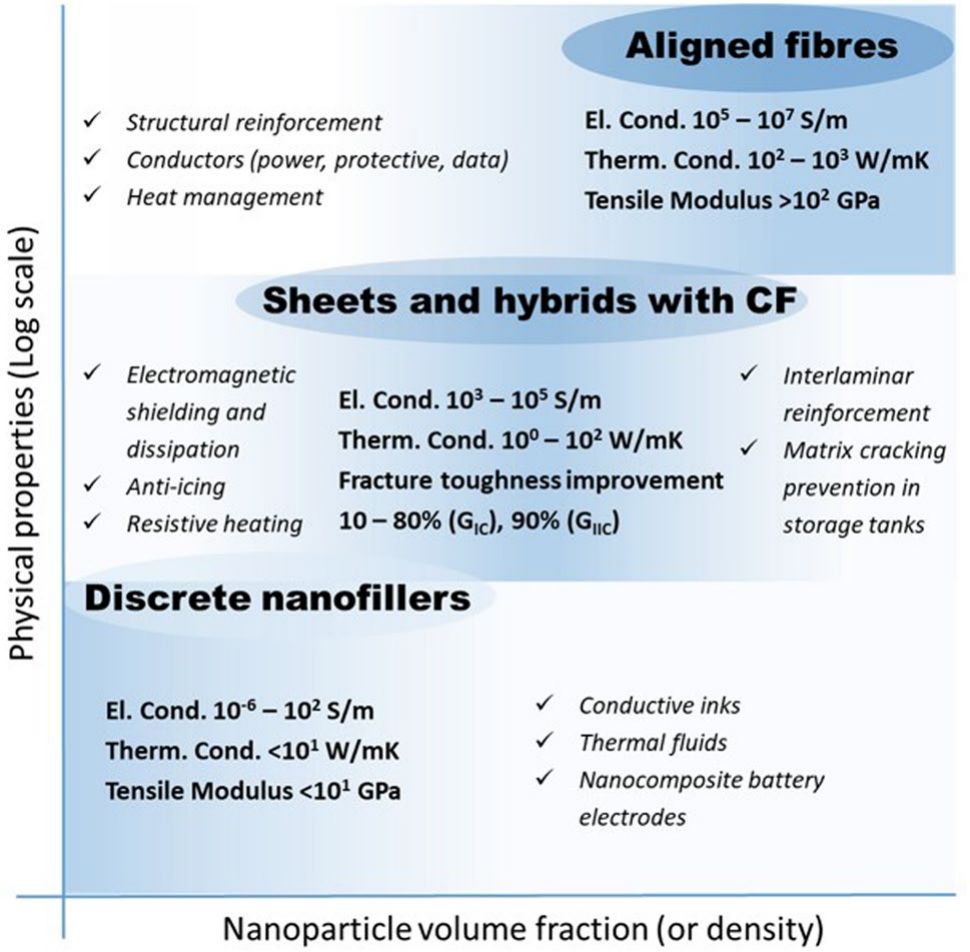
Map of properties of bulk materials of high-aspect ratio nanoparticles in different architectures. Increasing nanoparticle volume fraction towards dense solids produces increases in bulk properties from the level of polymers to above metals. Examples of the most promising applications in aircraft are listed. Copyright 2024 IMDEA Materials

By way of summary, in Fig. 56 we present a schematic plot of mechanical, electrical, and thermal properties (in log scale) for the different volume fractions (or density) achieved in the three common architectures of nanomaterials as randomly dispersed fillers in a matrix, as sheets, and as aligned fibres. We include promising applications specific to these embodiments, ranging from conductive inks to power cables.

Fully electric regional-jet aircraft or single-aisle hybrid electric aircraft with short/medium ranges are still viable without superconductor materials. In these and present aircraft, nanocarbon-based wires are a possible replacement for metals in power cables and other conductors. Nanostructured conductors can offer weight reductions of the order of 50% relative to metals, but so far, they have only been demonstrated in selected data transfer cables and protective systems. Pursuing similar reductions in power cables requires developing strategies for the stabilisation of dopants/intercalants in nanocarbon fibres under high current density conditions. Given the inherent dependence of cable performance on design and construction parameters, parallel efforts must be directed at scaling up fibre spinning and doping processes and increasing prototyping and testing of increasing gauge cables. Together with simulations of cable performance considering both electrical and thermal properties, these developments would accelerate the development of nanostructured cables.

In batteries, nanomaterials enable high energy density and high power density, improvements in integration factor, and increased safety. One strategy is to produce nanosized active materials, which reduces limitations from solid-state diffusion, thus increasing power density and eliminating electrochemical pulverisation under repeated charge/discharge cycles, increasing battery life. A complementary strategy is to form composite electrodes with a built-in network of percolated nanocarbons. This architecture can reduce the content of conductive agents, eliminating the need for polymeric binders and ultimately replacing metallic foil current collectors. For several high-energy density materials, most notably Li-metal batteries, the introduction of the nanostructured network also increases safety by eliminating dendrite formation. To impact the aerospace sector, though, these improvements need to be demonstrated on thicker electrodes at a multi-tonne scale and prove to be economically feasible to implement.

Supercapacitors will be increasingly integrated into future aircraft for powering new electronics, such as autonomous wireless sensor networks [285], high-power actuators for altitude control [286] and activation of electromechanical conduits, as well as their use to compensate the battery for power applications [287]. We envisage two main types of supercapacitors for aviation. The first are high power capacitors consisting of pseudocapacitive materials mixed with high aspect ratio nanocarbons, most likely processed through solution processing. Improvements in processing should focus on demonstrating the fabrication of thick electrodes and the reduction of nanocarbon functionalization to reduce electrochemical degradation after extended cycling. The other type is semi-structural supercapacitors that take load-bearing functions and thus reduce the weight of systems. These will be produced through the deposition of pseudocapacitive material on pre-formed C-based structures with high stiffness (nanocarbon fibres, electrospun CF or regular CF).

In fuel cells, the introduction of nanomaterials targets the catalyst, its support, and the membranes in the cell. In addition to enabling reductions in overall Pt catalyst content, nanostructured alloys can prevent catalyst poisoning. Nanocarbon catalyst supports are increasingly replacing granular C to increase corrosion resistance. In membranes, nanomaterials are introduced to modify the pore structure, transport properties, and mechanical and thermal properties. Polymeric membranes with a small fraction of nanocarbons, the most common embodiment, to consistently show increasingly selective gas permeability and reduce cross-over. Amongst the pressing needs for applying PEMFCs in aerospace is the development of key nanostructured materials (*i.e.*, the electrocatalysts, membranes, and corrosion-resistant bipolar plates) for high-temperature operation. For FCs, SOFC technology needs improvement in the reliability, durability, and efficiency of the hot recycle blower and steam reformer. Hydrogen fuel cells are constrained for higher power applications due to low specific energy, low specific power, thermal management techniques and airport infrastructure issues. The development of IT-SOFCs that can operate on hydrocarbon fuels (directly or with minimum fuel processing) is of great importance for high-energy applications.

To improve the mechanical properties of aeronautical structural components with nanomaterials, the dominant strategies are the combination of fillers or sheets with CF to produce hybrid composites or the replacement of CF with nanostructured fibres altogether. The first strategy is very advanced, with a demonstration of scalable fabrication of large panels using semi-industrial manufacturing methods and materials and reaching aerospace quality standards. Selective introduction of organised nanocarbons in these laminate composites produces increases in interlaminar mechanical properties and, in some cases, in compression after impact, the key entry properties for structures. Progress on nanocarbon fibres has been steady, with current tensile strength values above many CFs. Recent work suggests that the macromolecular assembly of nanocarbons can lead to combinations of strength and modulus beyond the current envelope of CF. Their progressive use as reinforcing fibres requires the production of much larger volumes of fibres and the demonstration of fabrication of composite panels (initially hybrid with CF) using established manufacturing routes. The increasing interest in hydrogen storage tanks, primarily designed for strength, offers a suitable ground for adopting nanostructured fibres and matrix reinforcement with nanofillers.

Thermal management is identified as a key area for the development of hybrid/electric aircraft. Nanofluids are likely to be used for thermal management of electronic and power systems. The range of properties attainable by dispersing nanofillers in fluids is reasonably established. Evaluating the improvements in performance requires tests in actual cooling systems. This will also enable a better assessment of the permissible increases in viscosity and help identify the optimal shape and size for nanofillers in thermal fluids. The other area of promise is LHP, which could exploit the unusual combination of high SSA and high thermal conductivity in nanostructured yarns of nanocarbons or BNNTs. Further work should be directed at studying the capillarity of these systems both experimentally and, given the small size of pores, through modelling.

Equipped with this analysis, in Table 18, we summarise selected applications of nanomaterials in aeronautical components.

**Table 18:** Selected applications of nanomaterials and related engineering hurdles

Application	Material	Current TRL	Main development required	Contribution to sustainability targets
Electrical conductors	Doped/intercalated nanocarbon fibres	3-4	Strategies for stabilisation of doped materials	Enabler of electric aircraft
			Demonstration of high ampacity in cable prototypes with the new generation CNT fibres	Up to 50 wt.% reduction in selected cables/mesh
Energy storage	Nanostructured composite electrodes for supercapacitors and batteries	8	Development of solid electrolytes for increased safety	Load levelling in electric/hybrid aircraft
			Improvements in capacity retention for high-power devices	~ 80% weight reduction in batteries
Thermal management: loop heat pipes	Sheets/fibres of BNNT or nanocarbons	2	Models for heat transfer and fluid flow in nanoporous materials	Enabler of electric aircraft
Structures: Cryogenic H_2_ storage tanks	Nanostructured fillers in CFRP	2	Modelling and experimental evidence of nanostructured fillers reducing matrix cracking	Enabler of electric aircraft
Structures: interlaminar reinforcement	Nanocarbon veils in CFRP	4	Demonstration of improvement in impact properties	~ 20% improvement of composite aircraft structure
				Annual reduction of > 100 tonnes of CO_2_ per aircraft

The table also includes our assessment of their current technology readiness level (TRL), the leading scientific/technical developments needed and the potential contributions to sustainability targets.

Finally, to highlight the urgent need to overcome these hurdles, we note that the qualification, certification, and implementation of new materials/processes for aircraft primary structure applications can take between 5 and 10 years, although this is now in the process of high acceleration thanks to digitalisation and out of cycle developments. Thus, applications envisaged for 2030 should be ready fairly soon, and thus, research and development in these areas must intensify immediately if the potential for nanomaterials in more sustainable aviation is to be realised.

## Data Availability

No datasets were generated or analysed during the current study.

## Footnotes

1 https://domminioproject.eu/
